# Exploring the roles of ribosomal peptides in prokaryote-phage interactions through deep learning-enabled metagenome mining

**DOI:** 10.1186/s40168-024-01807-y

**Published:** 2024-05-24

**Authors:** Ying Gao, Zheng Zhong, Dengwei Zhang, Jian Zhang, Yong-Xin Li

**Affiliations:** CYM305, Department of Chemistry and The Swire Institute of Marine Science, The University of Hong Kong, Pokfulam Road, Hong Kong Special Administrative Region, 999077 China

**Keywords:** Ribosomal peptide, RiPPs, Metagenome mining, Biosynthetic genes, Ocean microbiome, Ecological function of metabolites, Prokaryote-phage interaction

## Abstract

**Background:**

Microbial secondary metabolites play a crucial role in the intricate interactions within the natural environment. Among these metabolites, ribosomally synthesized and post-translationally modified peptides (RiPPs) are becoming a promising source of therapeutic agents due to their structural diversity and functional versatility. However, their biosynthetic capacity and ecological functions remain largely underexplored.

**Results:**

Here, we aim to explore the biosynthetic profile of RiPPs and their potential roles in the interactions between microbes and viruses in the ocean, which encompasses a vast diversity of unique biomes that are rich in interactions and remains chemically underexplored. We first developed TrRiPP to identify RiPPs from ocean metagenomes, a deep learning method that detects RiPP precursors in a hallmark gene-independent manner to overcome the limitations of classic methods in processing highly fragmented metagenomic data. Applying this method to metagenomes from the global ocean microbiome, we uncover a diverse array of previously uncharacterized putative RiPP families with great novelty and diversity. Through correlation analysis based on metatranscriptomic data, we observed a high prevalence of antiphage defense-related and phage-related protein families that were co-expressed with RiPP families. Based on this putative association between RiPPs and phage infection, we constructed an Ocean Virus Database (OVD) and established a RiPP-involving host-phage interaction network through host prediction and co-expression analysis, revealing complex connectivities linking RiPP-encoding prokaryotes, RiPP families, viral protein families, and phages. These findings highlight the potential of RiPP families involved in prokaryote-phage interactions and coevolution, providing insights into their ecological functions in the ocean microbiome.

**Conclusions:**

This study provides a systematic investigation of the biosynthetic potential of RiPPs from the ocean microbiome at a global scale, shedding light on the essential insights into the ecological functions of RiPPs in prokaryote-phage interactions through the integration of deep learning approaches, metatranscriptomic data, and host-phage connectivity. This study serves as a valuable example of exploring the ecological functions of bacterial secondary metabolites, particularly their associations with unexplored microbial interactions.

Video Abstract

**Supplementary Information:**

The online version contains supplementary material available at 10.1186/s40168-024-01807-y.

## Introduction

The microbial world is an abundant source of natural products, also known as secondary metabolites. These compounds have been extensively studied and served as therapeutic molecules in medical applications, particularly in the field of antimicrobial discovery. Natural products are believed to play a crucial role in numerous ecological processes, such as microbial defense systems, biofilm formation as signaling molecules, and long-distance mediation between microbes and their surrounding neighbors [1–3]. Among natural products, RiPPs have emerged as one of the largest and most promising sources of therapeutic agents due to their remarkable structural diversity and functional versatility [4]. Although there is growing interest in RiPPs due to their therapeutic potential, most of their ecological functions are not yet understood.

Recent reports have suggested that RiPPs provide microbes with various benefits, such as the ability to compete and communicate [2, 5, 6]. One noteworthy study by Sberro et al. [7] has proposed that small peptides with 50 or fewer amino acids in the human microbiome may act as antiphage defense peptides. Furthermore, four peptidic bacteriocins, including staphylococcin 188, enterocins AAR-71, AAR-74, and erwiniocin NA4, were reported to show antiphage activities against coliphage HAS [8]. In addition, recent studies have also found that anthracyclines and aminoglycosides antibiotics, secondary metabolites produced by *Streptomyces*, can provide broad chemical defense against phages [9, 10]. These findings suggest that RiPPs, as peptidic secondary metabolites, may function similarly to anthracyclines and aminoglycosides in prokaryote-phage interactions despite most known antiphage defense systems being protein- or RNA-based [11]. Recent global biosynthetic studies have shown that the ocean microbiota is equipped with a wide variety of biologically active peptidic secondary metabolites, which allow them to flourish in highly competitive environments [12, 13]. Particularly, the ocean microbiota is home to abundant viruses, making up the largest biological entities with a virus-to-microbial cell ratio of 10:1, leading to dynamic prokaryote-phage battles [14, 15]. This presents a unique opportunity to investigate the potential roles of RiPPs in the interactions between microbes and viruses.

Recent advances in metagenomic technologies have allowed scientists to explore the genomic and metabolic diversity of complex microbiota, waiving the limitation of studying solely cultivable microbes [13, 16, 17]. This innovative approach has opened up new avenues for investigating the biosynthetic landscape of RiPPs in complex microbiota and the social interactions in which RiPPs are involved [12, 15, 18]. Typically, the biosynthesis of RiPPs involves the ribosomal synthesis of a short precursor peptide, followed by extensive post-translational modifications (PTMs) by a series of PTM enzymes. The genetically encoded nature of RiPPs allows for the efficient expansion of their chemical space by diverse PTMs and makes their chemical structure predictable via genomic analysis. Consequently, significant efforts have been dedicated to genome mining-guided discovery of new RiPPs [19, 20]. Our recent research has focused on genomics-guided discovery of RiPPs, resulting in the identification of diverse antagonistic RiPPs from complex microbiome [21, 22]. By applying rule-based genome mining strategies, we have successfully revealed previously untapped post-translational modification (PTM) enzymes involved in RiPP biosynthesis [23, 24]. However, the identification of RiPP sequences from metagenomes is challenging due to the difficulties of recovering high-quality genome-resolved data. Previously, identifying RiPP biosynthetic gene clusters (BGCs) was primarily facilitated by querying genome-resolved data using traditional methods such as antiSMASH [25], which relied on discerning intact PTM enzymes. While accurate for high-quality genomes, this approach is inapplicable to highly fragmented contigs, which should be fully used to comprehensively explore the biosynthetic potential of ocean microbiome.

Herein, our research aimed to investigate the biosynthetic profile of RiPPs in the underexplored ocean microbiome via metagenome mining, linking their potential roles to prokaryote-phage interactions via correlation analysis. To overcome the obstacle of RiPP detection from highly fragmented metagenomes, we developed TrRiPP, a deep learning method that bypasses PTM enzyme detection and identifies RiPP precursors in a hallmark gene-independent manner. Using deep learning-aided metagenome mining, we discovered numerous previously unknown RiPP families with high novelty and diversity. Our analysis also revealed a potential link between RiPP families and phage-mediated competition and coevolution, as evidenced by the prevalence of antiphage defense-related and phage-related protein families that co-expressed with RiPPs. To further investigate this relationship, we constructed an Ocean Virus Database (OVD) to predict host-phage relationships and then established an interaction network connecting RiPP-encoding prokaryotes, RiPP families, viral protein families, and phages. Our findings suggest that RiPP families have the potential to play a significant role in prokaryote-phage competition and coevolution.

## Results

### A deep learning approach for metagenome mining of RiPPs

**Fig. 1:**
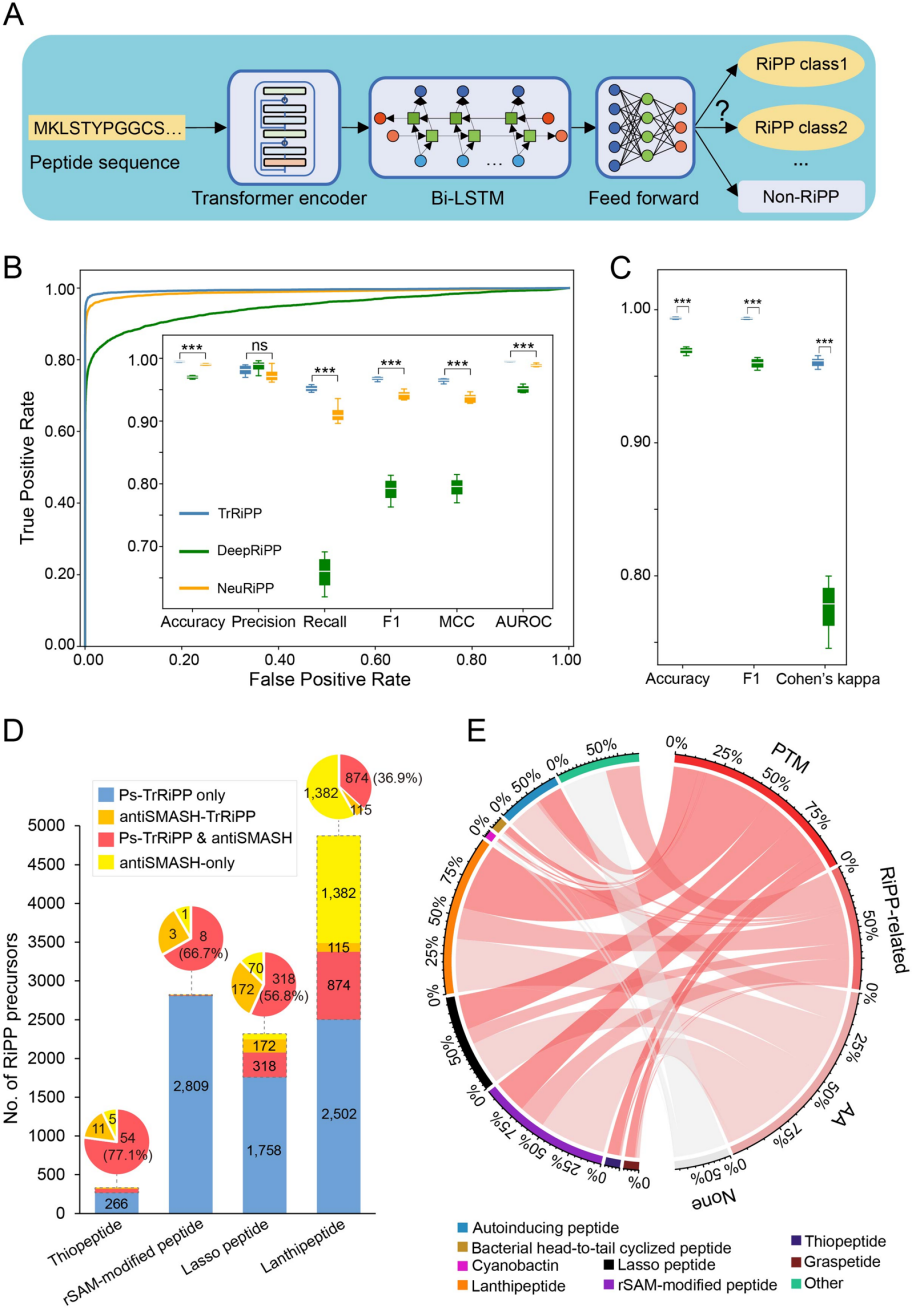
Development of deep learning approach to discover RiPP precursors. **A** The model combines a Transformer encoder with Bi-LSTM layers to discriminate RiPP precursors from non-RiPP short peptides and to classify RiPPs into subclasses. **B** Outer: receiver operating characteristic (ROC) curves of NeuRiPP (orange), DeepRiPP (green), and TrRiPP (blue). Inner: binary prediction performance (RiPP vs. non-RiPP) of NeuRiPP, DeepRiPP, and TrRiPP. **C** Multiclass classification performance (RiPP classification) of DeepRiPP and TrRiPP. NeuRiPP was not tested because it was designed solely to differentiate between RiPPs and non-RiPPs. **B**, **C**. Results of tenfold cross-validation are presented. In each box, the center bar represents the median, the box represents the upper and lower quartiles, and the whiskers represent 1.5 times the interquartile range. *P*-values are calculated by two-tailed *t*-tests (TrRiPP vs. NeuRiPP and TrRiPP vs. DeepRiPP). ***: *P* < 0.0005. **D** Comparing the performance of TrRiPP and antiSMASH on bacterial complete genomes. Orange regions indicate additional precursors predicted by TrRiPP when antiSMASH is used to annotate precursor ORFs instead of Prodigal-short (antiSMASH-TrRiPP, orange). For clarity, the proportions of RiPPs annotated by antiSMASH-TrRiPP, Ps-TrRiPP and antiSMASH, and antiSMASH-only are shown in the pie charts at the top. **E** Evaluation of RiPPs predicted by TrRiPP from bacterial complete genomes**.** PTM: The precursor contains the intrinsic amino acids and co-localizes with class-defining PTM enzymes. RiPP-related: The precursor contains the intrinsic amino acids and co-localizes with RiPP-related genes (such as transporters; Supplementary Data 2). AA, the precursor contains the intrinsic amino acids. None, no RiPP-related genes co-localize with the precursor. Autoinducing peptides, bacterial head-to-tail cyclized peptides, cyanobactins, and other RiPPs lack intrinsic amino acids, making them only eligible for assignment at the “RiPP-related” or “None” validity level

To explore the biosynthetic capacity and potential function of RiPPs in the global ocean microbiome, we first sought to detect the RiPP sequences from ocean metagenomes. To effectively classify RiPP precursors from highly fragmented metagenomes (Figure S1, S2) in a PTM-independent manner, we developed a deep learning model, TrRiPP, to accurately categorize RiPP precursors from peptide sequences ($\le$ 150 amino acids). The model combines Transformer and bidirectional long-short term memory (Bi-LSTM) [26], two common architectures in natural language processing that show promising capacity in processing biological data [27, 28] (Fig. 1A, S3). The model consists of a Transformer encoder, two Bi-LSTM layers, and a feed-forward neural network. The transformer encoder first encodes amino acid sequences to compute attention maps, which are then processed by the Bi-LSTM to obtain the sequence representations. The resulting sequence representations are then subjected to feed-forward layers for RiPPs classification. Ablation studies showed that both the Transformer encoder and the Bi-LSTM part of the model significantly impacted model performance (Figure S4). To train TrRiPP, we used a dataset derived from NeuRiPP [28] and DeepRiPP [27] training data, supplemented with manual curation from public databases and literature (Figure S5; Supplementary Data 1). To avoid overrepresenting similar RiPP sequences, we deduplicated the RiPP precursor sequences using CD-HIT [29] in the test set. Using tenfold cross-validation, we found that TrRiPP outperformed NeuRiPP and DeepRiPP in discriminating RiPP precursors from non-RiPP peptides on all metrics except for precision. Specifically, our model achieved 0.994 in accuracy, 0.953 in recall, 0.967 in F1 score (harmonic mean of the precision and recall), 0.964 in Matthews Correlation Coefficient (MCC), and 0.995 in area under receiver operating characteristic curve (AUROC), significantly improving upon NeuRiPP and DeepRiPP (*P* < 0.0005, two-tailed *t*-test) (Fig. 1B; Supplementary Table 1). For RiPP subclass classification, TrRiPP likewise achieved a superior performance than DeepRiPP (*P* < 0.0005, two-tailed* t*-test) in all three metrics, including accuracy (0.994), F1 score (0.993), and Cohen’s kappa coefficient (0.960) (Fig. 1C; Supplementary Table 2). Confusion matrices further showed that TrRiPP is substantially more accurate than DeepRiPP across all RiPP classes, indicating that TrRiPP is less biased towards prevalent data classes such as non-RiPP and lanthipeptides (Figure S6, S7). To demonstrate TrRiPP’s generalization capability for sequences distant from those in the training set, we present a benchmark that illustrates the model’s performance on proteins in the test set with varying degrees of similarity to the training data. The results indicated that TrRiPP performed well across the entire range of sequence similarities, from 10 to 100%, improving upon both NeuRiPP and DeepRiPP (Figure S8, S9). The final model, trained on the training set with no more than 90% similarity to the test set and provided with the tool, also performed well on sequences with identities as low as 30% to the training set (Figure S10).

### Validation of TrRiPP

To validate the generality of our model on external data, we applied TrRiPP to predict RiPP precursors in complete bacterial genomes. This allowed us to (1) compare the results with traditional genome mining methods such as antiSMASH and (2) identify PTM enzymes and other RiPP-related genes (such as transporters; Supplementary Data 2) that co-localize with precursor encoding genes, thereby validating our method. We applied TrRiPP to 22,789 bacterial complete genomes, predicting 33,474 putative RiPP precursors, including 12,421 unique sequences. To evaluate the performance of TrRiPP and antiSMASH, we analyzed four classes of RiPP precursors obtained from 22,789 bacterial complete genomes (RefSeq 2021.8), by the Prodigal-short/TrRiPP pipeline (Ps-TrRiPP; “Methods”), antiSMASH, or both. We observed that 36.9% (874/2,371, lanthipeptide) to 77.1% (54/70, thiopeptide) of putative precursors annotated by antiSMASH could be identified by TrRiPP (Fig. 1D). Since antiSMASH, which implements the RODEO algorithm to predict RiPP precursors [30], uses a different open reading frame (ORF) detection rule (“Methods”), we further applied TrRiPP to antiSMASH-annotated ORFs to avoid bias (Fig. 1D, antiSMASH-TrRiPP). This resulted in an additional 115 lanthipeptides, 172 lasso peptides, 11 thiopeptides, and three rSAM-modified peptides compared to the Prodigal-short/TrRiPP pipeline (Fig. 1D, Ps-TrRiPP). In total, 51.6% (1,555/3,013) of antiSMASH-annotated precursors were predicted by TrRiPP. Importantly, TrRiPP also identified hundreds to thousands of precursors that were not annotated by antiSMASH (RODEO algorithm) and enabled the prediction of the precursors of other RiPP classes, such as autoinducing peptides and graspetides (Fig. 1D; Supplementary Table 3). Next, we validated the improvements of TrRiPP over antiSMASH on fragmented genomes. We randomly selected 100 bacterial complete genomes from the pool of 22,789 and artificially fragmented their DNAs according to the contig length distribution derived from metagenomes. Then, we utilized both the Prodigal-short/TrRiPP pipeline and antiSMASH to predict RiPP precursors and compared the results to the predictions made on the unmodified complete genomes. When analyzing the 100 complete genomes, we found that antiSMASH identified 46 RiPP precursors. However, only 1 precursor was recovered from the fragmented versions of the genomes. In contrast, the Prodigal-short/TrRiPP pipeline predicted 217 RiPP precursors from these 100 complete genomes, and 109 of them could be recovered after the genomes were fragmented (Supplementary Data 3–6). Overall, our results demonstrate that TrRiPP is a powerful tool for identifying RiPP precursors independent of genetic context and can complement traditional genome mining methods to expand the scope of RiPP discovery.

We then evaluated the TrRiPP-predicted RiPP precursors by examining their sequence features and genomic neighborhoods. The chord diagram (Fig. 1E) shows all unique RiPP precursors predicted from 22,789 bacterial complete genomes, and their validity is determined by the genomic neighborhoods and the intrinsic amino acid residues (“Methods”). Specifically, we examined whether these precursors co-localized with the class-defining PTM enzymes (Fig. 1E, PTM), other RiPP-related genes, such as transporters (Fig. 1E, RiPP-related; Supplementary Data 2), and whether their sequences contained amino acids required for the PTM reaction to occur (Fig. 1E, AA). We found a high proportion of precursors co-localized with PTM enzymes or other RiPP-related genes on the genome, ranging from 43.0 to 91.9% (Fig. 1E). For putative precursors without surrounding PTM enzymes, we hypothesized that part of them might utilize more distant or substrate-tolerant enzymes like ProcM [31] for post-translational modifications. Our analysis showed that over 99% of putative precursors carried the intrinsic amino acids required for the post-translational modifications, such as cysteine and serine/threonine in lanthipeptide (Fig. 1E; Supplementary Table 4). These findings suggest that TrRiPP has learned the underlying sequence features of RiPP precursors. To further evaluate the predicted RiPPs, we constructed a sequence similarity network based on precursor sequences. We discovered that the majority of the large clusters in the network contained RiPP precursors that co-localized with corresponding PTM enzymes (Figure S11). For instance, the representative BGC from the largest cluster (graspetide) encodes an ATP-grasp enzyme alongside the corresponding precursor peptide (Figure S11).

Nevertheless, we also observed some ambiguously classified precursors. For example, the predicted rSAM-modified peptide cluster F1 contains precursors that co-localize with class I lanthipeptide-related PTM enzymes, which should be classified as lanthipeptides according to PTM-oriented genome mining methods. However, we noticed that the precursors from this cluster are cysteine-rich in their C-terminus (Figure S12), which is the common feature of rSAM-modified peptides and lanthipeptides, leading to ambiguous predictions. Despite the current ambiguity, we remain optimistic that TrRiPP will make less ambiguous predictions as more RiPPs are discovered and incorporated into its training data.

### Discovery of unknown RiPPs from underexplored ocean microbiomes

**Fig. 2:**
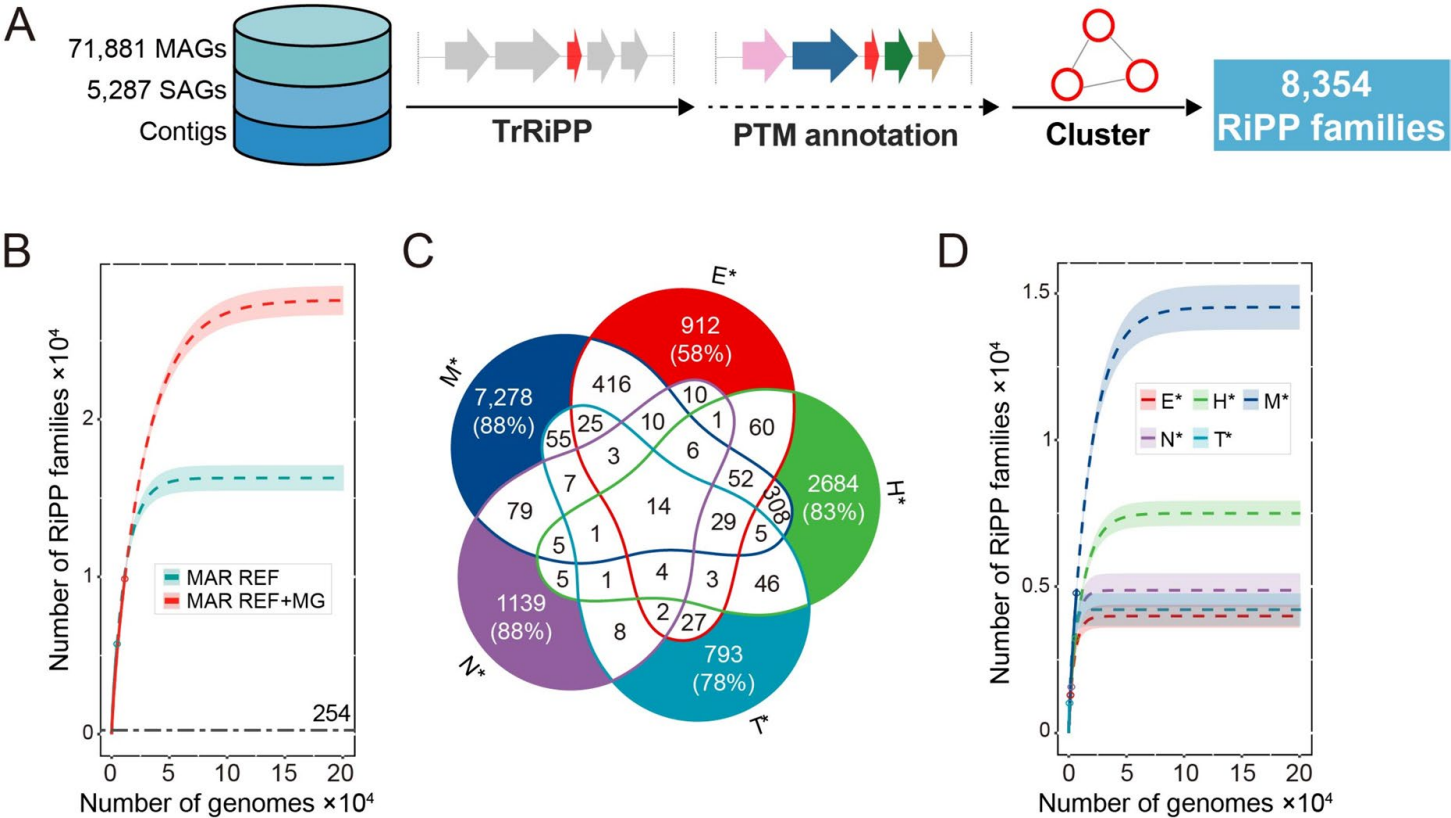
Over 8000 RiPP families were identified from ocean metagenomes. **A** The workflow of mining and annotating the nonredundant RiPP families. **B** Rarefaction curves of detected RiPP families from collected ocean metagenomes (MG) and the MAR database (MAR REF). The solid lines represent interpolated and actual data, while the dashed lines represent extrapolated data. The empty circles represent the observed values: 5732 (MAR REF) and 9868 (MAR REF + MG). The shadows represent the 95% confidence interval. The number of RiPP families documented in the MIBiG3 (grey dashed line, 254) corresponds to 0.9–1.6% of the predicted potential of the ocean microbiome (number of RiPP families at 0.2 million genomes). The *y*-axis values (number of extrapolated RiPP families) at the right end of the graph are 16,279.72 (MAR REF) and 27,563.25 (MAR REF + MG). **C** A five-set Venn diagram visualizes RiPP family intersections from five ecosystems (E* engineered, M* marine, H* host-associated, N* non-marine-aquatic, T* terrestrial). The values in the figure represent the number of shared or unique RiPP families among ecosystems. The niche-specific parts are highlighted with background color, and the uniqueness percentage is presented. **D** Rarefaction curves of detected RiPP families from five different ecosystems. The empty circles represent the observed values: 4777 (marine), 3224 (host-associated), 1574 (engineered), 1295 (non-marine-aquatic), and 1023 (terrestrial). The *y*-axis values (number of extrapolated RiPP families) at the right end of the graph are 14,530.50 (marine), 7,498.77 (host-associated), 3,989.90 (engineered), 4,873.13 (non-marine-aquatic), and 4,210.63 (terrestrial). In **B** and **D**, to reduce the biases from different genome types in the rarefaction analysis, we only use RiPP families from genomes, MAGs, and SAGs, without unbinned contigs. The MAGs from the host-associated, engineered, non-marine-aquatic, and terrestrial are collected from Earth’s microbiomes (GEM) catalog [35] (“Methods”)

Having developed a practical deep learning approach for RiPPs discovery independent of PTM enzymes, we next sought to investigate the biosynthetic repository of RiPPs encoded in the underexplored ocean microbiomes (Fig. 2A, **“**Methods”). We collected 71,811 metagenome-assembled genomes (MAGs) and 5287 single amplified genomes (SAGs) from 3035 seawater samples and unbinned contigs from 3562 metagenomes (“Methods”). These samples were mainly amassed from *Tara* Oceans [32], *Tara* Oceans Polar Circle [32], and Malaspina expeditions [33], which cover different stations, layers, and size fractions and thus represent the global ocean microbiome catalog. Initially, the genomic data collected (hereafter metagenomes) was analyzed using Prodigal-short/MetaProdigal-short to identify small ORFs. These small ORFs (*n* = 95,310,599) were then subjected to TrRiPP to predict RiPP precursors, resulting in the discovery of 19,864 nonredundant prokaryotic RiPP precursors. The Conserved Domain Database (CDD) was utilized to annotate the PTM enzymes located near the RiPP-encoding genes (up to 10 genes upstream/downstream). To facilitate comparative analysis across different data resources and genome types, the RiPP precursor sequences were clustered based on a 60% amino acid identity and 60% coverage, identifying 8354 RiPP families from metagenomes (Supplementary Data 7). The majority of the identified RiPP families were previously uncharacterized, with 4704 (56%) not represented in the MIBiG [34], a deposition database exclusively for experimentally validated BGCs. Our finding was consistent with a recent analysis of ~ 40,000 BGCs predicted by antiSMASH from 1038 metagenomes deposited in the Ocean Microbiomics Database (OMD), which showed a high novelty of diverse RiPP BGCs. We found that around 94% (7837/8354) of RiPP families from ocean metagenomes showed medium-to-low (≤ 60%) similarity with OMD (Figure S13; Supplementary Information), revealing the potential for discovering novel RiPPs from ocean microbiomes using our TrRiPP approach.

Next, we compared the RiPP families in ocean metagenomes with those in isolated marine bacterial genomes. We collected 13,050 genomes of isolated marine microbes from the MarRef (v1.6) and MarDB (v1.5) databases [36] (hereafter MAR REF). From these genomes, 5732 RiPP families (12,451 RiPPs precursors) were identified, of which only 894 families were represented in metagenomes **(**Figure S14**)**. The rarefaction analysis showed that the inclusion of metagenomes presented a steeper and highly unsaturated curve, indicating their potential to expand the biochemical space of novel RiPPs compared to isolated genomes **(**Fig. 2B; Supplementary Data 8). To further evaluate the chemical diversity of RiPPs, we applied the non-linear dimensionality reduction technique (densMAP [37]) to the chemical fingerprints [38] of RiPP precursor sequences. The RiPP precursor sequences from marine metagenomes were found to occupy a more discrete space in the manifold than those from MAR REF, indicating that marine metagenomes expanded the chemical space of the RiPPs occupied by isolated marine microbes (Figure S15). This was further supported by the lower average Tanimoto coefficient (a measure of chemical similarity [39, 40]) of RiPP precursor sequences in metagenomes (0.599) than that in isolated genomes (0.614). In addition, a high proportion (74%) of RiPP families from metagenomes showed medium-to-low similarity ($\le$ 60%) to those from isolated genomes (Figure S13, MAR REF; Supplementary Information). The findings highlight the significant untapped potential of novel RiPPs in the ocean microbiome, indicating the essential contribution of uncultured marine microbes to the unexplored RiPPs.

**Fig. 3:**
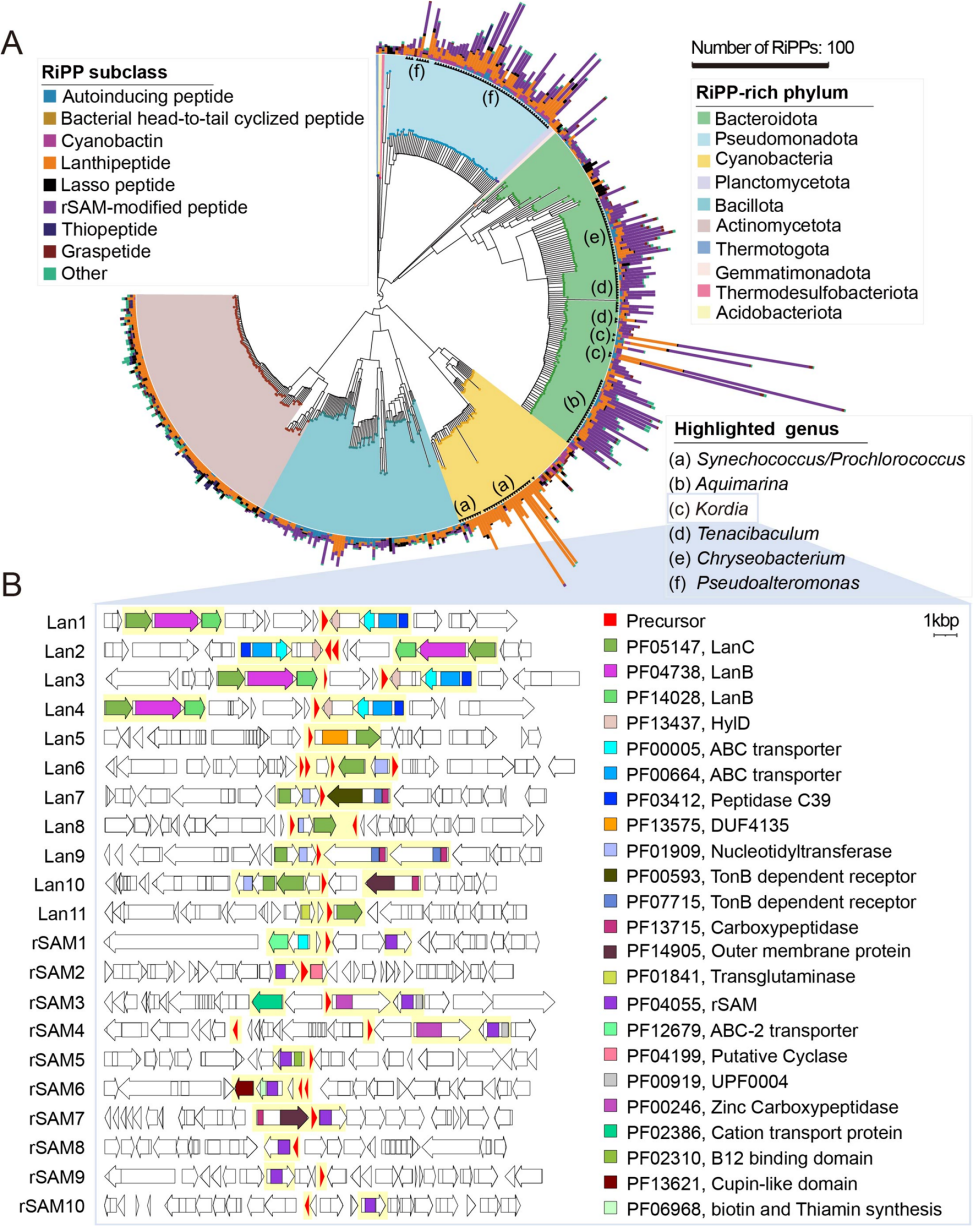
Ocean represents a largely untapped source of novel RiPPs. **A** The 428 RiPP-rich (the number of RiPPs on the genome $\ge$ 5) prokaryotic genomes (Figure S16, S17) were placed onto the phylogenomic trees and decorated with the number of RiPP subclasses using iTOL (v6) [45]. The stacked bar plots in the outer layer indicate the number of RiPP subclasses encoded by each genome. The different phyla and RiPP subclasses were rendered using different colors. The highlighted RiPP-rich genera were labeled with the alphabet. **B** The selected BGCs from *Kordia*. The core regions of BGCs were rendered using yellow backgrounds. The labels of BGCs, such as “Lan1” and “rSAM1,” represent the classified lanthipeptide and rSAM-modified peptide based on the class-defining enzymes

In order to expand our understanding of the biosynthetic capabilities of RiPPs found in the ocean microbiome, we analyzed their distribution across phylogenomic trees. To accomplish this, we defined RiPP capacity as the number and density (the number of RiPP precursor genes per kilobase pair of the genome) of RiPPs on the genomes. Our findings revealed that Bacteroidota was the most RiPP-rich phylum, with up to 176 RiPP precursors found on the genome, followed by Pseudomonadota and Cyanobacteria (Figure S16, S17), in agreement with a previous report [41]. Additionally, we observed a dense distribution of RiPPs from these three phyla, particularly in metagenomes, which suggests that there is still untapped biosynthetic potential in well-studied marine phyla (Figure S18). To gain further insight, we placed the genomes from the RiPP-rich phyla onto the phylogenomic tree and utilized the number of RiPPs per genome to embellish the tree (Fig. 3A). We identified several RiPP-rich genera, such as *Synechococcus* and *Prochlorococcus*, which are well-known genera encoding large numbers of lanthipeptides [42]. Notably, the Flavobacteriaceae family, well-known for its capability to produce terpenes, polyketides, and non-ribosomal peptides [41], exhibited noteworthy potential in encoding RiPPs, specifically rSAM-modified peptide, and lanthipeptide, exemplified by the genus *Kordia* (Fig. 3B, S19, S20; Supplementary Information). Furthermore, *Aquimarina* [43, 44] and *Tenacibaculum*, which have rarely been studied, also displayed a remarkable biosynthetic potential for RiPPs. Our analysis of metagenomes revealed a highly dense genomic distribution of RiPPs (Figure S18), probably due to the short length of metagenome assemblies. Nonetheless, the hidden potential of RiPP biosynthesis cannot be disregarded. In particular, Acidobacteriota, Planctomycetota, and Bacillita_A were RiPP-rich phyla in metagenomes and showed the dense genomic distribution of RiPPs, but similar patterns could not be found in isolated genomes (MAR REF).

We next sought to investigate the niche specificity of marine RiPPs. We found that marine RiPP families are highly niche-specific, with 7278 (88%) RiPP families uniquely detected in marine but not found in other ecosystems [35] (engineered, host-associated, non-marine-aquatic, and terrestrial; Fig. 2C). Interestingly, the ocean microbiome exhibited a greater biosynthetic potential for RiPPs, with tens of thousands of unknown RiPPs yet to be characterized (Fig. 2D; Supplementary Data 9), also represented by the higher ratio of RiPP-encoding operational taxonomic units (OTUs) (Figure S21B). Our analysis also revealed that RiPP-encoding OTUs showed specificity among different ecosystems (Figure S21A), with unique RiPP-encoding OTUs from host-associated ecosystems mainly belonging to the Bacillota phylum, while those from the ocean were predominantly from Pseudomonadota. The niche-specific RiPP families may provide a competitive advantage to the niche adaptation of their hosts, including mediating the inter-/intra-species and interkingdom interactions [7, 46, 47].

### RiPPs may involve prokaryote-phage interactions

**Fig. 4:**
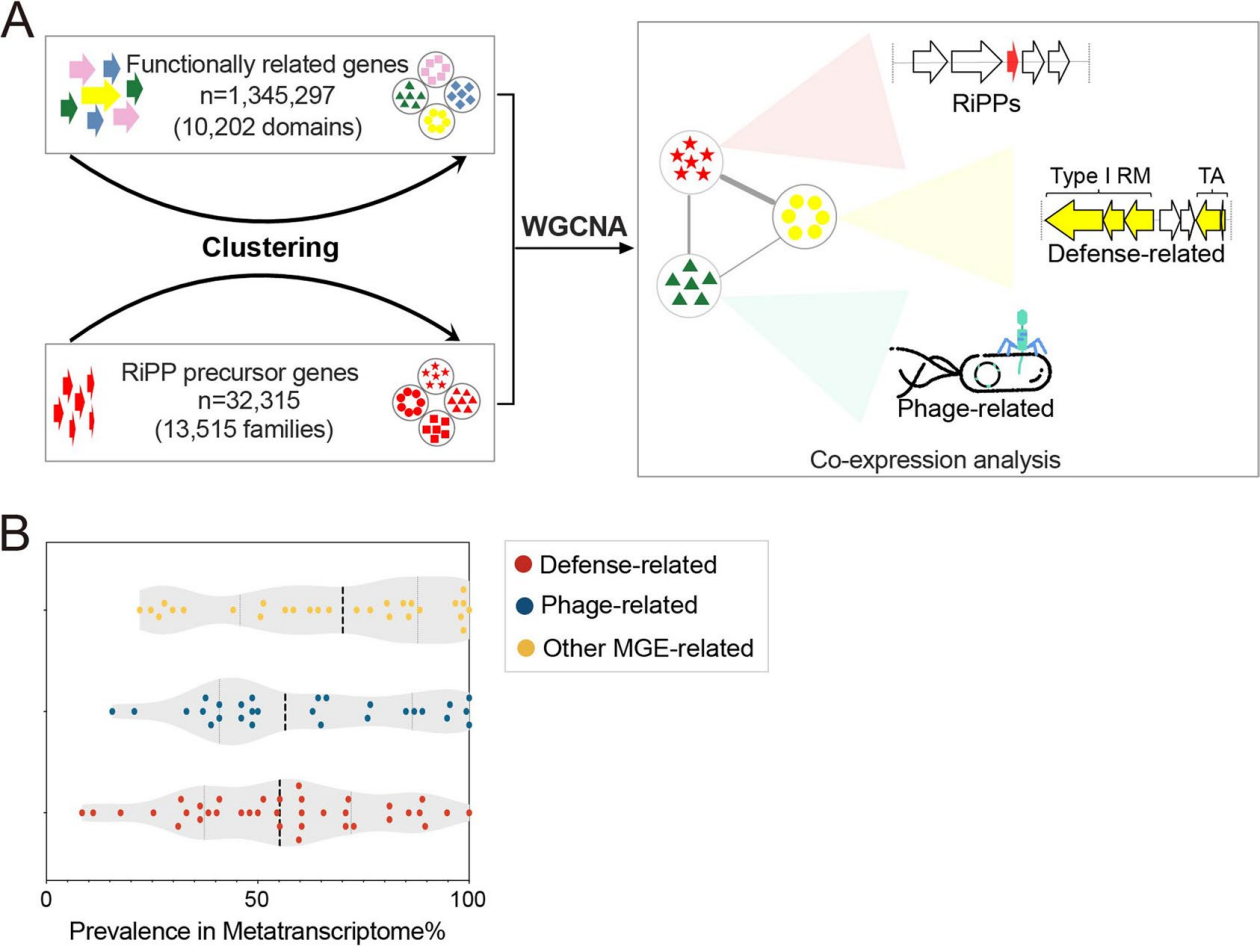
Correlation analysis suggests putative ecological functions of RiPPs in ocean microbiome. **A** The co-expression analysis between the RiPP families and protein families ($\le$ 50 kbp upstream/downstream). **B** The prevalence of 93 protein families co-expressed in modules with RiPP families). The corresponding abundance in metatranscriptomes is shown in Figure S24. The truncated violin plot was used, and the median and quartile values were shown as the dashed lines in dark and grey, respectively. Each dot represents one protein family

To gain a deeper understanding of the ecological roles of RiPPs in the ocean microbiome, we aimed to infer their potential functions based on the correlation with functionally related genes. To achieve this, we performed a co-expression analysis (employing the WGCNA package [48]) of RiPP families and protein families (≤ 50 kbp away from RiPPs) in metatranscriptomes (Fig. 4A). This approach has been successfully used in previous studies to infer the functions of unannotated genes from their annotated partners [47, 49, 50]. We revisited 154 metatranscriptomes from 154 globally distributed sampling sites (latitudinal range, − 64.4° ~ 79.3°) and multiple depth layers (from 5 to 800 m) (Figure S22**)**. We focused on the cellular size fraction [33, 51, 52] (> 0.2 μm) to ensure comprehensive ocean microbial communities coverage. Additionally, we integrated the ocean metagenomes and the MAR REF into a unified ocean genomic database (OGD) containing 21,171 RiPP-encoding genomes and contigs, which encode 13,515 RiPP families. To streamline the abundance inferences and reduce redundancy, we grouped the predicted RiPP neighboring genes from OGD into families based on Pfam [53] domain annotation (Fig. 4A).

Co-expression analysis revealed that 1933 protein families were positively correlated in 5 modules with 159 RiPP families (Supplementary Table 5, Supplementary Data 10). As expected, a considerable proportion of correlations within modules (37.1%) were associated with RiPP biosynthesis (Figure S23). Interestingly, we observed that numerous RiPP families (125) were correlated in modules with various mobile genetic elements (MGEs, 93 families), especially those associated with antiphage defense systems (“Phage-related” in Fig. 4A, e.g., phage genes that integrated or recombined into host genomes; Figure S23). These MGE-related genes are significantly enriched in 3 co-expression modules (modules 2, 3, and 7 in Supplementary Table 5) among the entire set of MGE-related genes in metatranscriptomes (hypergeometric test, *P* < 0.05), while the biosynthesis-related genes are significantly enriched in 5 co-expression modules (modules 1, 2, 3, 5 and 7 in Supplementary Table 6; hypergeometric test, *P* < 0.05). The high prevalence of mobile genetic elements that co-expressed with RiPPs, as depicted in Fig. 4B, implies a possible connection between RiPP families and phage-mediated competition and coevolution. The “Other MGE-related” protein families (mobile genetic elements, except “Defense-related” and “Phage-related”), such as “transposases” and “recombinases,” could also facilitate inter/intra-species transformation of RiPPs and defense-related genes for niche adaptation [46, 54].

**Fig. 5:**
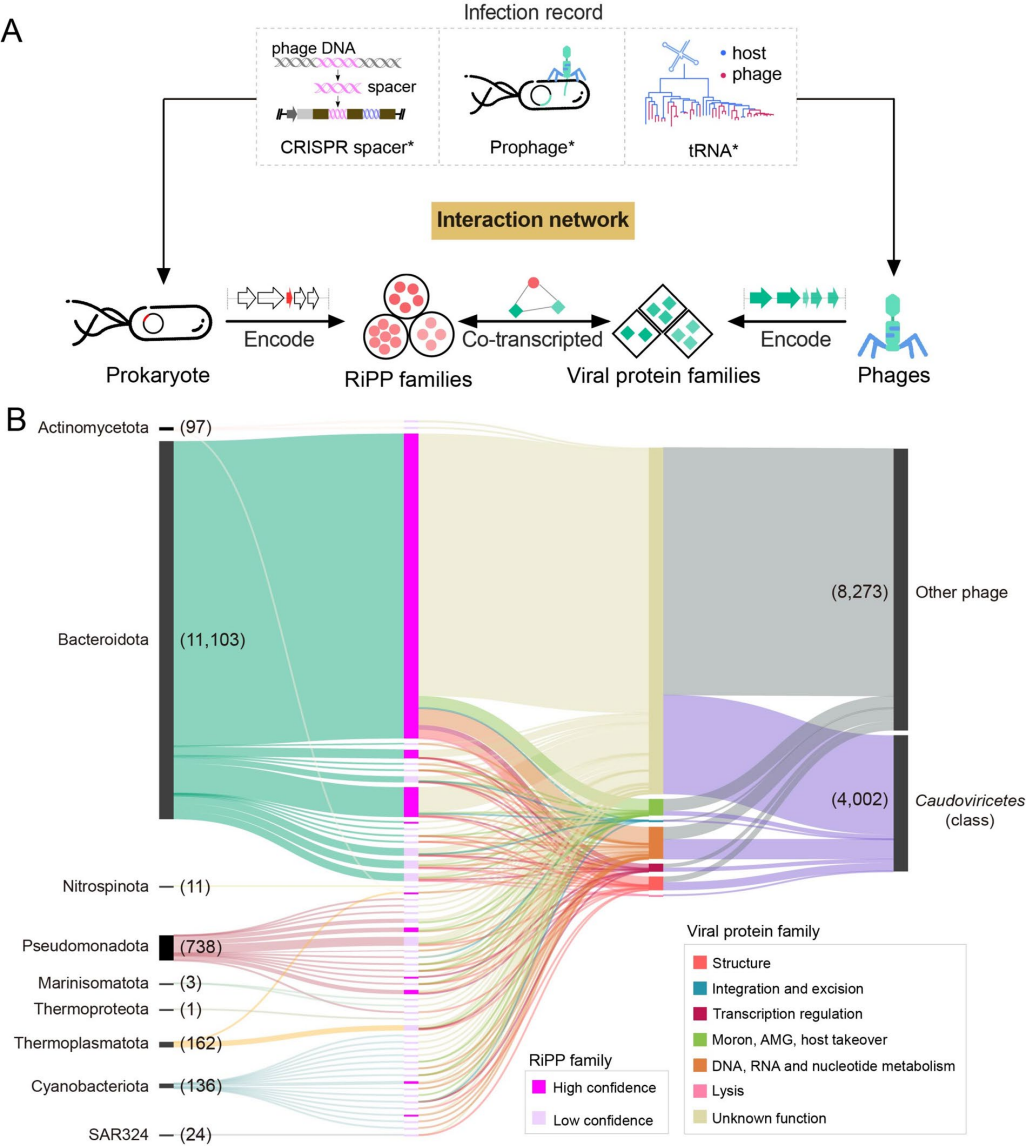
Uncovering the link between RiPPs and prokaryote-phage interactions through interaction networking. **A** The proposed RiPP-involving host-phage interaction network. **B** The prioritized RiPP-involving phage-host interaction network. For visualization, the prioritized network with WGCNA adjacency larger than 0.125 was selected and shown in the Sankey diagram, and the whole network (WGCNA adjacency > 0) was summarized in Figure S29. The node column from left to right denoted the prokaryotic phyla (host), RiPP families colored by confidence (at least one member of the RiPP family having neighboring PTMs within ten genes in the vicinity is defined as high confidence, and vice versa; “Methods”), functional categories of viral protein families, and phage taxon. The numbers in the brackets represented the flows (interactions) between nodes. For example, Bacteroidota was the source of 11,103 flows, and *Caudoviricetes* was the target of 4002 flows. The character “class” in the bracket represented the phage class

To further investigate the connectivity of RiPPs and phage infection, we sought to construct a phage-RiPPs interaction network, which was initially established from the relationships between the RiPP-encoding hosts and phages based on the infection records revealed from the host CRISPR spacers, shared tRNA, and host-integrated prophage (Fig. 5A). To accomplish this, we sought to (1) construct a comprehensive global ocean virus database and (2) predict the range of hosts the identified viruses infect. It is notoriously challenging to detect viral sequences from environments [55, 56], because viruses lack a universal marker gene, as opposed to bacterial 16S rRNA. Previous studies have established several databases for viruses [57–59]. However, our understanding of ocean viruses is still limited. In order to conduct a more comprehensive analysis, we constructed an Ocean Viruses Database (OVD), which consists of 1,647,134 non-redundant viral contigs (“Methods”) with Caudoviricetes emerging as the predominant phage class (> 80%, Figure S25). This led to the identification of 902,732 vOTUs (95% Average Nucleotide Identity, 85% Alignment Fraction), increasing species-rank viral populations by 3.1-fold and the number of viral gene groups by 2.3-fold compared to Global Oceans Viromes (GOV2) [58]. Among them, 99.9% vOTUs did not contain isolated viruses [60, 61], and 63.5% of viral proteins had no significant sequence similarity to proteins from publicly available protein databases (KEGG, Pfam-A, VOGDB) [53, 62]. The host prediction of phages also presents an intricate challenge, as the host information is often absent in the previous survey of viral metagenomes. Here, we leveraged the constructed OGD and OVD databases as the host-phage pairwise searching pool and built the host-phage connections based on the infection records revealed from the host CRISPR spacers, shared tRNA, and host-integrated prophage [57, 63, 64] (Fig. 5A, S26, S27; “Methods”). We obtained 249,691 nonredundant host-phage pairs spanning 58 prokaryotic phyla and 208 orders (Figure S28). Pseudomonadota was the dominant phylum involved in the prokaryote-phage interaction, followed by Bacteroidota, Cyanobacteria, and Thermoplasmatota. All of the prokaryotic phyla had predicted predators from the Caudoviricetes class (Figure S28), and the majority of these connections were identified for the first time, including 12 prokaryotic phyla for which no virus was previously reported [65] (https://genome.jgi.doe.gov/portal/IMG_VR).

**Fig. 6:**
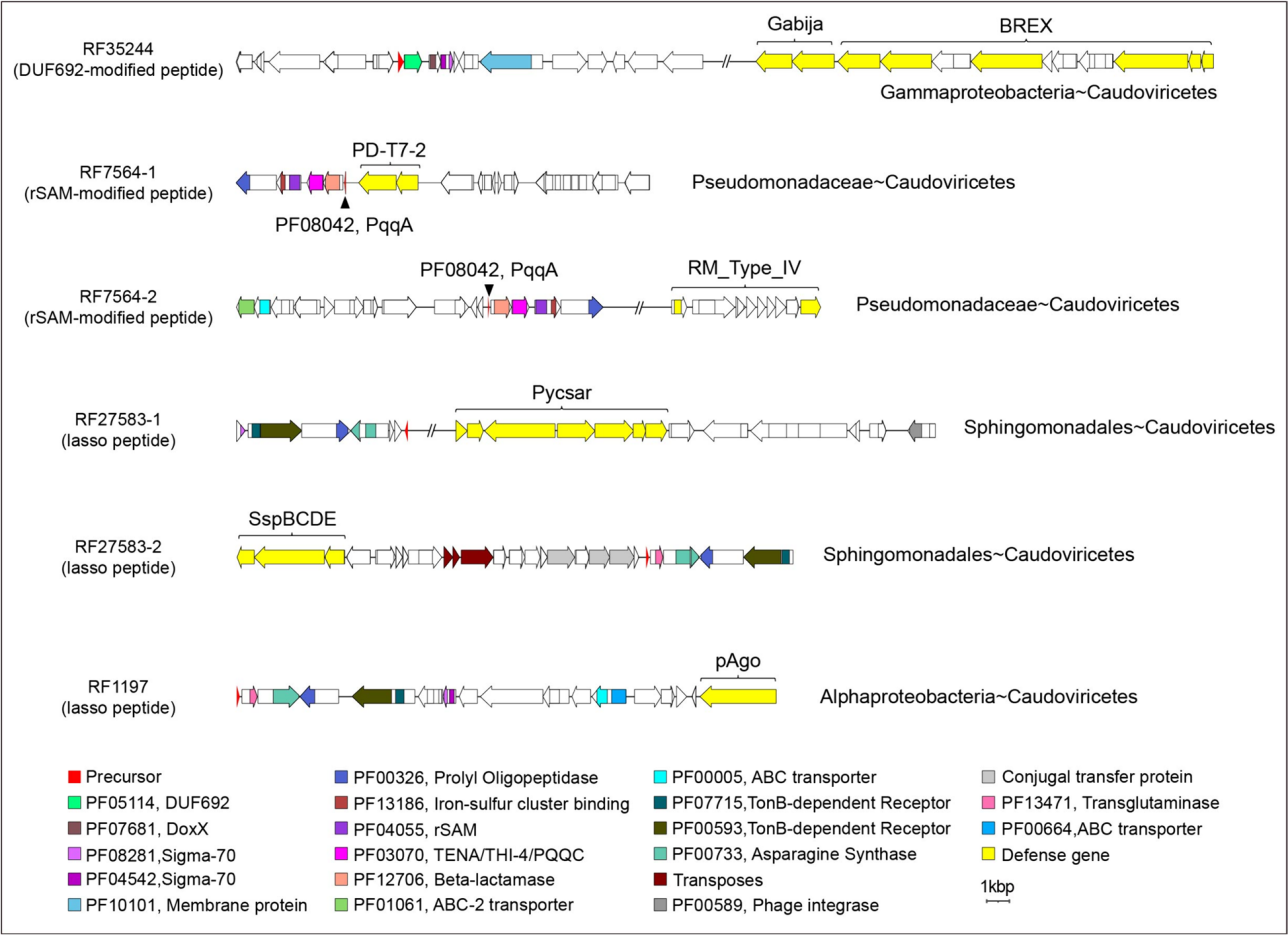
Representative RiPP families in the prokaryote-phage interaction network. The BGCs of the representative RiPP families (RF35244, RF7564, RF27583, RF1197) in the interaction network were plotted, and only the core regions were colored. The RiPP precursor of the known family was labeled using the corresponding Pfam domains. The defense systems co-expressed with the neighboring RiPP families ($\le$ 50 kbp upstream/downstream) were plotted and labeled. The RiPP subclasses in the bracket were assigned based on the class-defining PTMs. The bacteria-phage interactions that the corresponding RiPP families may mediate were labeled on the straight right side of the BGCs (except for RF35244, on the right bottom of the BGC)

Next, we examined the co-expressions between RiPP and viral protein families at the community level based on the established phage-host relationships. This allowed us to establish the network connecting RiPP-encoding prokaryotes, RiPP families, viral protein families, and phages, as shown in Fig. 5A. We performed a weighted gene co-expression network analysis (WGCNA) [48] based on the transcripts of 2953 RiPP families and 86,408 viral protein families detected in marine samples. This resulted in 97,698 pairs of co-expressed RiPP and viral protein families filtered by phage-host relationships. Notably, 544 RiPP families linked to prokaryote-phage interactions were mainly encoded by Pseudomonadota, Bacteroidota, and Cyanobacteria (Fig. 5B, S29A). Prominent examples of RiPP families that were represented in our analysis include putative DUF692-modified RiPP [66–68] (RF35244), rSAM-modified peptide (RF7564) and lasso peptide (RF27583, RF1197) (Fig. 6). We observed that these RiPP families co-expressed with diverse defense systems in their vicinity (≤ 50 kbp upstream/downstream), revealing their potential to defend against phages. As an example, we observed that RiPP family 7564, known as a rSAM-modified peptide family and annotated as the redox cofactor precursor PqqA, exhibited co-expression with two distinct defense systems, namely PD-T7-2 and type II RM system. This finding suggests a potential involvement of RiPP family 7564 in mediating or involving interactions between Pseudomonadaceae and Caudoviricetes within the ocean microbiome or vice versa. Similarly, the co-expression of the lasso peptide RF27583 with the Pycsar and SspBCDE defense systems suggests its potential role in involving interactions between Sphingomonadales and Caudoviricetes. Furthermore, this RiPP family exhibits colocalization with mobile genetic elements, including transposons, indicating the potential for transferring RiPPs and defense systems between individuals. These findings highlight these RiPPs may play a crucial role in environmental adaptation, particularly in prokaryote-phage competition.

## Discussion

The ocean microbiota boasts diverse biologically active peptidic secondary metabolites, enabling them to thrive in highly competitive environments. These metabolites play a crucial role in microbial interactions, including prokaryote-phage interactions, which are particularly important given the ocean’s high virus-to-microbial cell ratio. Despite this, the research on these peptides’ potential roles has been lacking. We discovered diverse RiPP families in the ocean microbiome using deep learning-based metagenome mining and revealed a potential link between RiPPs and phage-mediated competition and coevolution. Additionally, applying metatranscriptomic-based correlation analysis, we constructed an interaction network connecting RiPPs and prokaryote-phage, highlighting the significant role of RiPPs in prokaryote-phage interactions.

Metagenomic technologies have revolutionized our ability to explore the genomic and metabolic diversity of complex microbiota, overcoming the limitations of studying only cultivable microbes [13, 16, 17, 47]. However, recovering high-quality genome-resolved data is still challenging for metagenomes with high community diversity [69]. Rather than relying solely on genome-level data, in this study, we included genomic types of MAGs, SAGs, REFs, and unbinned contigs to better understand the biosynthetic repository of RiPPs in the global ocean microbiome. Previous metagenome mining of RiPPs has largely relied on identifying biosynthetic genes physically clustered on chromosomes or plasmids, focusing on enzymes critical for post-translational modifications but less on RiPP precursors [25, 70, 71]. While these approaches have successfully discovered new RiPPs from high-quality MAGs [12, 18, 35, 49], their application to most metagenomic data is challenging due to their features of high fragmentation. To overcome this challenge, we developed TrRiPP, a sequence-based RiPP classifier that can accurately identify RiPPs from fragmented metagenomic data independent of PTM enzymes. While precursor-based RiPP detection methods are crucial for studying RiPPs in highly fragmented metagenomes, their performance can still be improved due to limited training data. For instance, the percentage of putative RiPP precursors lacking neighboring PTM enzymes (Fig. 1E) can still be reduced. While some of these precursors may indeed be novel RiPPs, it is possible that some of them may be mispredictions. Besides, misclassification between rSAM-modified peptides and lanthipeptides remains an issue (Figure S11). However, we believe the increasing number of newly validated RiPPs can contribute to enlarging the training database and enhancing the performance of sequence-based RiPP classifiers.

The ocean is the largest ecosystem on earth, yet the biosynthetic potentials of ocean microbiomes remain largely uncharted [12, 35, 72, 73], as evidenced by recent studies Qin et al. [17] and Mukherjee et al. [31], who unearthed tens of thousands of BGCs from genome-resolved metagenomic data using antiSMASH to investigate the biosynthetic profile of ocean microbiomes. For instance, Paoli et al. [12] identified 6046 putatively novel RiPP BGCs from the Ocean Microbiome Database. Building upon this work, Wei et al. [43] analyzed high-quality genomes from an expanded metagenomic dataset and discovered 11,572 RiPP-encoding BGCs, with only 853 detectable precursor sequences. In our study, we identified a staggering 19,864 RiPP precursor sequences and over 8000 RiPP families from the global ocean microbiome, largely expanding the chemical space of RiPPs harbored in nature. Most of these families were previously uncharacterized, with over 90% showing medium-to-low similarity with OMD. Our findings underscore the remarkable diversity of RiPPs, characterized by highly variable nucleotide sequences in their precursors and numerous potentially novel biosynthetic pathways. For example, we observed the colocalization of LanC cyclase and nucleotidyltransferase around precursor peptides from the RiPP-rich genus *Kordia*. Moreover, our study underscores the significant impact of marine metagenomes, particularly those derived from uncultivated microbes, in broadening the chemical and biochemical diversity of RiPPs beyond what can be achieved through the cultivation of the marine microbiome alone. Interestingly, the RiPP families identified from marine metagenomes exhibit remarkable specificity towards specific ecological niches. However, it is crucial to acknowledge that the incomplete genetic context of contigs and the limited synthetic biology strategies for translating their genetic potential into chemical reality pose significant challenges for efficient metagenome mining-guided discovery of metabolites. Nevertheless, there is a reason for optimism as advancements in long-read technologies [74], and the emergence of new metagenome mining and synthetic biology approaches hold great promise in significantly improving the efficiency and accuracy of metagenomics-guided discovery of secondary metabolites [75].

Microbial secondary metabolites play a vital role in the intricate interactions within complex microbiota. However, previous studies on the ecological functions of secondary metabolites have focused primarily on individual cases, leaving a gap in our comprehensive understanding of their ecological roles in microbiota. While aminoglycosides and anthracyclines, known for their antiphage activities, have demonstrated their role in phage defense in vitro [9, 11], the ecological functions of secondary metabolites, particularly peptidic secondary metabolites, remain poorly understood [8]. Fortunately, with the accumulation of genomic sequencing data, large-scale analyses have facilitated our understanding of diverse biological processes [50]. In this study, we addressed this knowledge gap by conducting extensive correlation analyses to detect co-expressed gene pairs in metatranscriptomes. This approach allows us to infer the functions of unannotated genes based on their annotated partners, thereby shedding light on the potential ecological functions of secondary metabolites in microbiota. Our findings suggest that RiPPs may be linked to prokaryote-phage coevolution, as evidenced by the high prevalence of antiphage defense-related and phage-related protein families correlated with RiPP families in metatranscriptomes. This is consistent with the findings of Sberro et al. [7], which suggests that small peptides have the potential to colocalize with antiphage defense systems at the genomic level. However, there is a significant research gap when it comes to investigating peptides or molecules in the phage-host interaction network, especially at the community level, which would provide more comprehensive and direct evidence. In order to address this knowledge gap, we took the initiative to construct an Ocean Virus Database (OVD) and establish a host-phage interaction network involving RiPPs. This network enabled us to explore the connections between RiPP-encoding prokaryotes, RiPP families, viral protein families, and phages within complex communities. Our analysis identified 544 RiPP families that were involved in prokaryote-phage interactions. Notably, we observed the presence of putative DUF692-modified RiPPs, rSAM-modified peptides, and lasso peptides (Fig. 6), which also exhibited co-expression with diverse defense systems in their vicinity, indicating their potential role in defending against phages. It is important to note that while the correlation analysis provides insights into the potential functions of RiPPs in prokaryote-phage interactions, further experimental validations are necessary to confirm the causation within the network and elucidate the underlying interaction mechanisms.

Despite the aforementioned limitations, this study successfully provides a systematic elucidation of the biosynthetic potential of RiPPs from the ocean microbiome at a global scale, shedding light on the essential insights into the ecological functions of RiPPs in prokaryote-phage interactions. Notably, this is the first attempt to integrate deep learning-aided metagenome mining, metatranscriptomic data, and host-phage connectivity to investigate the involvement of RiPPs in prokaryote-phage coevolution. This study serves as a valuable example of exploring the ecological functions of bacterial secondary metabolites, particularly their associations with unexplored microbial interactions.

## Methods

### Training data for deep learning model

Known RiPP precursor sequences were obtained from publicly available databases including MIBiG [34], RiPPMiner [76], NeuRiPP [28], and DeepRiPP [27] training sets, and manual literature searches (Supplementary Data 1). Non-RiPP short peptide sequences were obtained from NeuRiPP and DeepRiPP training sets as well as from randomly selected bacterial short open reading frames (ORFs) annotated with non-RiPP domains by RPS-BLAST (v2.10.1 +) [77]. To avoid sequences in the test set being too similar to those in the training set, we further refined these data using CD-HIT [29] (v4.8.1). First, we clustered all sequences with a similarity threshold of 90% and retained one representative (determined by CD-HIT) in each cluster. In this process, we did not observe any misclustering of sequences among different RiPP classes (e.g., a lanthipeptide clustered with a lasso peptide), suggesting that 90% sequence similarity is an appropriate threshold. Second, we randomly selected 10% of these representatives as the test set while preserving the RiPP class distribution. Finally, we used the remaining 90% of representatives and their cluster members as the training set. This process ensured that sequences in the test set were less than 90% similar to those in the training set, while retaining as many sequences in the training set as possible. The final dataset consists of 43,620 non-RiPP short peptides and 6192 RiPP precursors, with 4689 sequences in the test set and 44,812 sequences in the training set.

### Development of deep learning model

TrRiPP is a deep learning model to identify RiPP precursor peptides for nine classes of RiPPs, including autoinducing peptide, bacterial head-to-tail cyclized peptide, cyanobactin, lanthipeptide, lasso peptide, graspetide, rSAM-modified peptide, thiopeptide, and other RiPPs. To facilitate the use of peptide sequences in deep learning models, tokenization was employed. Each of the 20 basic amino acids was encoded as a unique number, while any special or unknown amino acids were assigned a distinct number. The result of tokenization was a vector of numbers that represents the original peptide sequence, which can be utilized as the input for the deep learning model.

The architecture of the model started with a transformer encoder that takes tokenized peptide sequences as input. The transformer encoder has three steps: (1) the inputs are fed into two embedding layers. The first one further converts the indices of each amino acid into a fixed-size vector, which provides more information about the amino acid, while the second one encodes the positional information of each amino acid residue in the sequence, e.g., the same amino acid in different positions is treated differently; (2) the outputs of the embedding layers are passed through a self-attention layer. Briefly, in self-attention, the model computes a query, a key, and a value for each input token and computes a weighted sum of the values using the similarity scores between the query and key vectors. The resulting weighted sum is used as a new representation of the input token. This allows the model to selectively attend to different parts of the input sequence based on their relevance to the prediction task; (3) a feed-forward layer summarizes the attentions and outputs the encodings. Steps 2 and 3 are repeated several times in the transformer encoder to further empower the model.

In the subsequent step, the sequence encodings obtained from the transformer encoder were utilized as input for two layers of Bi-LSTM [26]. Long Short-Term Memory (LSTM), a recurrent neural network (RNN), consists of an LSTM cell that iteratively processes each amino acid in a sequence, storing and combining information with the subsequent amino acid. LSTM can generate the hidden states for that sequence by iterating through all amino acid residues. To capture a more comprehensive understanding, Bi-LSTM traverses the sequence in both forward and backward directions. In order to enhance the model’s performance, we employed two stacked Bi-LSTM layers, where the output of the first LSTM layer served as input to the second Bi-LSTM layer. Next, we concatenated the maximum, mean, and last outputs from the last Bi-LSTM layer and fed the concatenated vector into a feed-forward network. This final network consists of two dense layers with a rectified linear unit (ReLU) as the activation function. The output of the last dense layer was then converted to the probability of each RiPP class by a softmax function. A detailed scheme of the model is shown in Figure S3.

Dropout is considered as a beneficial approach to reduce overfitting in deep learning models by randomly omitting a portion of the parameters [78]. We applied this technique to all parts of our model, including the connection between two dense layers, the connection between two Bi-LSTM layers, and the calculation of the attention weights, etc.

The hyperparameters used to build the model are listed below:

- Batch size: 128
- No. of transformer encoder layers: 4
- No. of attention heads: 4
- Dimension of embeddings in the transformer encoder: 128
- Dimension of feed-forward networks in the transformer encoder: 256
- No. of Bi-LSTM layers: 2
- Hidden dimension of LSTM cell: 64
- Dimension of feed-forward network: 192
- Dropout: 0.4

The deep learning model was implemented in Python 3 using the PyTorch library (https://pytorch.org/). The Transformer encoder was built by the Hugging Face Transformers library [79].

### Model training and evaluation

To evaluate the performance of TrRiPP, we compared it to two other deep learning models that also classify RiPP precursors, namely DeepRiPP and NeuRiPP. Since NeuRiPP consists of five algorithms, we evaluated each one and found that “cnn-parallel-lstm” performed the best. Therefore, it was chosen for all downstream comparisons (Supplementary Table 1). Since the original models were trained on different datasets, we retrained them on the same dataset to ensure a fair comparison. The training of DeepRiPP and NeuRiPP was based on the standard procedures described in their respective papers and codes. For the training of TrRiPP, we used the AdamW optimizer [80] with a scheduled learning rate that gradually increased from 0 to 0.001 in the first 10,000 training steps before linearly decreasing to 0 in the remaining steps. The model was trained for a maximum of 200 epochs but stopped after 50 epochs with no improvement. To account for the highly unbalanced data, Focal Loss [81] was used as the loss function instead of the standard cross-entropy loss:

$${\text{Softmax}}({x}_{i})=\frac{{e}^{{x}_{i}}}{\sum_{j=1}^{C}{e}^{{x}_{j}}}$$

$$FL\left({x}_{c}\right)={-\left(1-{\text{Softmax}}({x}_{c})\right)}^{\gamma }{\text{log}}({\text{Softmax}}\left({x}_{c}\right))$$

where $C$ is the number of classes, ${\text{Softmax}}({x}_{c})$ is the model’s estimated probability for the class $c$, and $\gamma$ is the focusing parameter. We used $\gamma =1$ in our training process.

Binary classification performance (distinguishing RiPPs from non-RiPPs) was assessed using six metrics, accuracy, precision, recall, F1-score, Matthews Correlation Coefficient (MCC), and area under receiver operating characteristic (AUROC). Except for AUROC, all metrics were calculated by the number of true positive (TP), true negative (TN), false positive (FP), and false negative (FN) predictions:

$${\text{Accuracy}}=\frac{{\text{TP}}+{\text{TN}}}{{\text{TP}}+{\text{TN}}+{\text{FP}}+{\text{FN}}}$$

$${\text{Precision}}=\frac{{\text{TP}}}{{\text{TP}}+{\text{FP}}}$$

$${\text{Recall}}=\frac{{\text{TP}}}{{\text{TP}}+{\text{FN}}}$$

$${\text{F}}1=\frac{2{\text{TP}}}{2{\text{TP}}+{\text{FP}}+{\text{FN}}}$$

$${\text{MCC}}=\frac{{\text{TP}}\times {\text{TN}}-{\text{FP}}\times {\text{FN}}}{\sqrt{({\text{TP}}+{\text{FP}})({\text{TP}}+{\text{FN}})({\text{TN}}+{\text{FP}})({\text{TN}}+{\text{FN}})}}$$

The AUROC was calculated based on the TP and FP as well as their corresponding scores provided by the model. NeuRiPP and DeepRiPP calculate the probability scores for their predictions, so we directly took the scores for positively predicted outcomes and the negative scores for negatively predicted outcomes to plot the ROC curve. However, TrRiPP does not provide a score for its prediction. Instead, it presents the probability of the result in 10 classes (9 RiPP classes and 1 non-RiPP), which is calculated through a softmax function. Thus, we plotted the ROC curve using the score obtained by subtracting the probability of the peptide being predicted as non-RiPP from 1 (1—P(non-RiPP)).

The metrics used to evaluate the multi-class classification consisted of accuracy, weighted F1 score and Cohen’s kappa.

$${\text{Accuracy}}= \frac{1}{N}\sum\nolimits_{i=1}^{N}1({{\widehat{y}}_{i}=y}_{i})$$

where $N$ is the number of all samples, ${\widehat{y}}_{i}$ is the predicted value of the $i$-th sample, and ${y}_{i}$ is the corresponding true value.

$$\mathrm{Weighted\ F}1= \sum\nolimits_{c=1}^{C}\frac{2{{\text{TP}}}_{c}}{2{{\text{TP}}}_{c}+{{\text{FP}}}_{c}+{{\text{FN}}}_{c}}\cdot \frac{{N}_{c}}{N}$$

where $N$ is the number of all samples, $C$ is the number of classes, ${N}_{c}$ is the number of predictions in class $c$, and ${{\text{TP}}}_{c}$ is the number of true positives in class $c$, and so on.

$$\mathrm{Cohen^{\prime}}\mathrm{s\ kappa}= \frac{\mathrm{Accuracy }-{ p}_{e}}{1 - {p}_{e}}, {p}_{e}= \frac{1}{{N}^{2}}\sum\nolimits_{c=1}^{C}{n}_{c1}{n}_{c2}$$

where $N$ is the number of all samples, $C$ is the number of classes, and ${n}_{ci}$ is the number of times that rater $i$ predicted class $c$.

Cohen’s kappa is a statistic used to compare labeling by different annotators and is considered to be a more robust measure than accuracy. In this specific case, we compared the model to the ground truth.

We performed tenfold cross-validation to train and test all models. The metrics reported in this paper represent their tenfold average. All statistics and calculations for the ROC and confusion matrix were performed in Python 3 using the Scikit-learn library [82]. The ROC and confusion matrix figures were plotted using the Matplotlib library [83].

### Ablation study of deep learning model

We estimated the importance of different components of the model through training and testing a series of ablation models:

- Full model: the full TrRiPP model described in the “Methods” section.
- LSTM: model without the transformer encoder. In this model, an embedding layer with the same hidden dimension as the transformer encoder was used to encode the input tokens.
- Transformer: model without the Bi-LSTM layers. In this model, we used the *BertForSequenceClassification* model architecture in the Huggingface Transformers library, where the representations of the first token (the CLS token) in an input sequence were passed through a feed-forward neural network for RiPP classification.
- Transformer max: an updated version of the previous ablation model using a max pool layer to aggregate token representations. The pooled representations were then passed through a feed-forward neural network for RiPPs classification.

Hyperparameters of all ablation models were kept the same as the full model whenever possible. We trained all ablation models on the same dataset and performed the same tenfold cross-validation to assess model performance. Satistical significance of performance metrics was determined using two-tailed Student’s *t*-test.

### Model generalization capability assessment

All training data were clustered by MMseqs2 (v13.45111) [84] using a sequence identity threshold ranging from 10 to 100%. Next, the representative sequence in each cluster was randomly split into a training set, validation set, and test set with an 8:1:1 ratio while preserving the RiPP class distribution in each set. TrRiPP, NeuRiPP, and DeepRiPP were then retrained and tested on these datasets using their default parameters. Finally, the metrics described in the “Model training and evaluation” section were used to evaluate the testing results.

To assess the generalization ability of the final model (the model trained on the training set described in the “Training data for deep learning model” section), we partitioned the test set based on its sequence identity to the most similar sequence in the training set. The most similar sequence was defined as the top-hit sequence from a MMseqs2 search, and the global sequence identity was calculated using Biopython v1.79.

### Genomic and metagenomic data collection

Seventy-seven thousand one hundred sixty-eight metagenome-assembled genomes (MAGs) and single amplified genomes (SAGs) from 3035 seawater samples and unbinned contigs ($\ge$ 1kbp) from 3562 metagenomes were amassed from publicly available databases and literature [12, 33, 35, 52, 72, 85–87] (Supplementary Data 11). For the assembled metagenomic data from the paper by Nayfach et. al [35]. only 1289 marine samples were selected, among which 1284 (Supplementary Data 12) were successfully retrieved from the JGI-IMG/M database (https://img.jgi.doe.gov/) and used for downstream analysis. The MAGs and the corresponding OTUs from the paper by Nayfach et. al [35]. for the comparison of different ecosystems (engineered, host-associated, non-marine-aquatic, and terrestrial) were downloaded from https://portal.nersc.gov/GEM/ (Supplementary Data 13). For Marine, the MAGs, and SAGs from our collected genomic data were used for comparison.

Isolated marine microbial genomes were selected from the MARREF (v1.6) and MARDB (v1.5) databases [36]. This data was further filtered by removing entries that were present in the historical assembly summary in NCBI (retrieve date: 2022/01/04). In total, we obtained 13,050 valid entries that were deduplicated and retrievable from NCBI RefSeq or GenBank databases. Since the genomes in RefSeq database are better annotated, we chose RefSeq as the primary download option. We downloaded genomes from GenBank only if they were not present in RefSeq. Finally, we downloaded and analyzed 5445 genomes from RefSeq and 7605 genomes from GenBank (retrieve date: 2022/01/04, Supplementary Data 14).

One hundred fifty-four metatranscriptomes were collected from *Tara* Oceans ($0.22-3\mu m$, *n* = 154) [13, 52] (Supplementary Data 15). Sequencing reads from all metatranscriptomes were quality filtered using BBMap [88] (v.38.79) by removing sequencing adapters from the reads, removing reads that mapped to quality control sequences (PhiX genome), and discarding low-quality reads using the following parameters: minlen = 45, qtrim = rl, trimq = 20, ktrim = r, mink = 11, hdist = 1, k = 25, tbo, tpe. Metatranscriptomic reads mapped to sequences in a ribosomal sequence database were removed using SortMeRNA v4.2.0 (–ref smr_v4.3_sensitive_db_rfam_seeds.fasta) [89].

### ORFs detection and RiPPs prediction

We used Prodigal-short [90], a gene detection tool modified for detecting small ORFs in prokaryotes, on all genomic and metagenomic assemblies. Genetically encoded small ORFs were predicted by Prodigal-short (Prodigal (v2.6.3) with parameters adjusted to include ORFs ≥ 45 bp) in either meta mode or normal mode based on their sources. Small ORFs were filtered to include only those with a start and stop codon, a ribosomal binding site, and a length ≤ 450 bp. In particular, a ribosomal binding site was not required for ORFs in Cyanobacteria, Chlorobi, Chlamydiae, Ignavibacteriae, and Verrucomicrobia, where a large percentage of ORFs lacked the Shine-Dalgarno sequences [91, 92] (Figure S30). Finally, we applied TrRiPP to distinguish and classify RiPP precursors from valid small ORFs. This pipeline is referred to as Ps-TrRiPP in Fig. 1D.

AntiSMASH predicted RiPPs were obtained by analyzing all bacterial complete genomes in the RefSeq dataset (accessed in Aug. 2021, *n* = 22,789) using antiSMASH v6.0 with default parameters (Supplementary Data 16).

To compare the RiPP precursors predicted by TrRiPP and antiSMASH, we also applied TrRiPP to antiSMASH-annotated ORFs. This additional step was taken due to antiSMASH’s implementation of the RODEO algorithm, which has an aggressive small ORF detection strategy. It predicts ORFs from all six frames and does not account for ribosomal binding sites or stop codons. Therefore, we first extracted the ORFs of antiSMASH-annotated RiPP precursors and applied TrRiPP to them. This process would avoid the bias arising from different ORF detection strategies. This pipeline is referred to as antiSMASH-TrRiPP in Fig. 1D.

### Evaluation of predicted RiPP precursors

For RiPP precursors predicted from bacterial complete genomes, we defined four exclusive validity levels: PTM, RiPP-related, AA, and None. The top 2 levels, PTM and RiPP-related, consider neighboring genes, which are defined as genes found within ten genes upstream or downstream of the precursor gene.

Specifically, for cyanobactin, lanthipeptide, lasso peptide, rSAM-modified peptide, and thiopeptide, which have class-defining Pfam domains, we searched their neighboring genes against the Pfam database (v35.0) using hmmsearch (http://hmmer.org/, v3.3.1) with the default parameters. Domain hits were filtered by score > 0. For graspetide, we used InterProScan [93] (v5.54–87.0) with the default parameters and searched for the keyword “ATP-grasp” in its results to find graspetide PTM enzymes. These classes of RiPPs, except for cyanobactin, all require specific amino acids in their precursor sequences to enable modification (e.g., Cys and Ser/Thr are required to form lanthionine or labionin ring in lanthipeptide). Therefore, we defined RiPP precursors fulfilling the requirements of the class-defining PTM enzyme domains and the amino acids as validity level 1 (PTM). For a complete list of amino acid requirements, please refer to Supplementary Table 4.

For validity level 2, we annotated the RiPP-encoding genes and their neighboring genes against the Conserved Domain Database [94] (CDD, downloaded in June 2020) using RPS-BLAST (v2.10.1 +). Domain hits were considered significant if the value ≤ 0.001 (the default threshold) and the alignment length of the query was at least 80% of the length of PSSM. If any of the RiPP-related genes (Supplementary Data 2) matched the RiPP-encoding gene or appeared in its neighboring genes, the RiPP precursor was assigned to validity level 2.

Validity level 3 extended validity level 1 by removing the PTM enzyme domain requirements. Namely, precursors at this validity level contained the intrinsic amino acids in their sequence but lacked the corresponding PTM enzymes in their neighboring genes. Validity level 4 referred to all other precursors.

### Visualization of predicted RiPP precursors and their corresponding BGCs

For TrRiPP prediction results from bacterial complete genomes, we collected predicted RiPP precursors within the top 2 validity levels described in the “Methods” section. To fully demonstrate the power of TrRiPP, we filtered out precursors which can also be identified by antiSMASH, as well as precursors with more than 90% similar to sequences in the training data, calculated by MMseqs2. We then constructed a sequence similarity network for these filtered precursors using EFI-EST [95] with the default parameters (E-value = 5, alignment score = 10) and visualized it by Cytoscape [96]. Next, we selected nodes from each RiPP class in each cluster and illustrated their BGCs and Pfam domains by BiG-SCAPE [97]. Key Pfam domains for each RiPP class are defined in Supplementary Table 4.

### Construction of RiPP families and confidence assignment

Valid small open reading frames (ORFs) were subjected to TrRiPP for RiPP prediction. To allow for comparative analysis between different data resources and genome types (MAGs, SAGs, REFs and contigs), RiPP precursor sequences were clustered into RiPP families using MMseqs2 (v13.45111) [84]. To determine the suitable threshold for short sequence clustering, we compared MMseqs2 results on 193 RiPP precursor sequences from MIBiG [34] and literature versus the compound-based clustering provided by the NPAtlas database (Figure S31; Supplementary Data 17) [98]. Considering the shared motif of the RiPP core region, GLAM2 [99] was used to calculate the sequence alignment scores of RiPP families (Figure S32). A final optimized parameter of coverage mode 2 and 60% sequence identity and coverage (full command for clustering: mmseqs easy-cluster < input > < output > < tmp > -c 0.6 –min-seq-id 0.6 -e 10 –cluster-reassign 1 –cov-mode 2) was chosen based on the similarity of MMseqs2 clustering results to the NPAtlas compound clusters and minimizing the number of RiPP families with low sequence alignment scores (score < − 500). Unless otherwise stated, RiPPs precursor sequences were clustered by MMseqs2 with these optimized parameters.

ORFs within ten genes of predicted RiPP-encoding genes were then analyzed by RPS-BLAST (v2.10.1 +) [77] with default parameters against the Conserved Domain Database [100] (CDD, downloaded in June 2020) for domain annotation. Domain hits were considered significant if the evalue ≤ 0.001 and the alignment length of the query was at least 80% of the length of PSSM. If any of the RiPP-related genes matched the RiPP-encoding gene itself or appeared in its neighboring genes (≤ 10 genes upstream/downstream), the RiPP precursor was considered as the one with PTM. If any member of the RiPP family was the one with PTM, then this RiPP family was assigned as “high confidence” and vice versa.

### Sequence clustering and identity search

To calculate RiPP sequence identity, we used all nonredundant precursor sequences as queries to search against the database using BLASTP (v2.12.0 +) with default parameters. For each query sequence, the sequence identity of the target sequence with the lowest e-value was reported. When calculating the sequence similarity between RiPP families and the database, the all-to-all searches were conducted, and for each member of a RiPP family, the maximum sequence identity against the database was retained. All maximum sequence identities were averaged to define the mean maximum sequence identity of the RiPP family against the database.

### Taxonomic classification of RiPP-encoding contigs and genomes

The RiPP-encoding contigs and genomes (MAGs, SAGs, and MAR REF) were annotated using CAT (v5.1.2, default parameters) [101] and GTDB-TK (v2.1.1, GTDB release 207) [102, 103], respectively. The taxonomy database of CAT was downloaded in January 2021. The taxonomy of RiPPs was determined by the corresponding contigs and genomes. To remove putative viral, eukaryotic, and ambiguous contigs and genomes, the workflow described in the “Identification of viral sequences” section (“Methods”) was firstly run through all RiPP-encoding contigs and genomes to estimate the potential of viral source. Besides, a voting approach that considered the nucleotide acid sequence on a query with hits to the NT database using MEGABLAST (v2.12.0 + ; Figure S33) [60, 77] was used to estimate the ratio of different kingdoms in a given contig or genome. Only if the prokaryote dominates the taxonomic classification, or the putative viral, eukaryotic, and ambiguous fragments constitute less than 50% of the length, the contig or genome will be retained for further analysis. For the unassigned contigs and genomes, the MEGABLAST results with more than 50% voting were employed (Supplementary Data 18).

### Rarefaction analysis

Extrapolation of the potential number of RiPP families was achieved by conducting rarefaction analyses using the iNEXT R package [104]. A RiPP family presence/absence table (RiPP family-by-genome/contig matrix) was constructed for each group considered and was then used as “incidence-raw” data in the iNEXT main function, where 2,000 points were interpolated or extrapolated with an endpoint of 1.2 million for marine metagenomes and isolated genomes (MAR REF). By default, the number of bootstrap replications is 50.

### RiPPs chemical space comparison

We first clustered RiPPs precursors predicted from isolated marine microbes and marine metagenomes with 60% sequence identity and coverage using MMseqs2 with the following parameters: easy-cluster < input > < output > < tmp > -c 0.6 –min-seq-id 0.6 -e 10 –cluster-reassign 1 –cov-mode 2. Then we calculated the ECFP6 chemical fingerprints [38] of the representative precursors by The Chemistry Development Kit [105]. Finally, we computed the Jaccard distance matrix based on the chemical fingerprints, and performed dimension reduction by densMAP [37] with n_neighbors = 15 using the UMAP Python package.

In addition, we calculated the pairwise Tanimoto coefficient of each precursor using NumPy and scikit-learn and averaged all these Tanimoto coefficients to obtain the average Tanimoto coefficient.

### Identification of MGE-related genes

Mobile genetic elements (MGEs) are protein-encoding DNA that mediate DNA movement within genomes or between prokaryotic cells [106]. MGEs could be phages, plasmids, integrative, transposable, and conjugative elements with more than 50 subcategories [107]. Based on our interest, we only focus on the three classes of “Defense-related,” “Phage-related,” and “Other MGE-related”. To identify defense-related neighboring genes, the domains identified within 50 kbp from the RiPP-encoding genes were queried against the list of Pfams downloaded from Table S1 in Doron et al. (2018) [108] as well as against the words “CRISPR,” “BREX,” and so on. To identify phage-related genes, the words “phage,” “virus,” “viral,” and “integrase” were queried. To identify other MGE-related genes, the words “transposase,” “transposon,” and “recombinase” were queried. To supplement the identification of MGEs, DefenseFinder (–db-type gembase) [109] and MobileOG-db (recommended protocol) [107] were used.

### Identification of viral sequences

Due to the significantly underestimated viral abundance and diversity, numerous tools have been developed to detect viral signals in metagenomes. The combination of different types of tools has been widely reported as an effective approach to distinguish viral sequences [57, 58]. In this study, we sought to integrate the results of similarity-based VirSorter2 [56] and k-mer-based VirFinder [55], benchmarking universal single-copy orthologs (BUSCO) [110] and curated viral protein family modules (VPFs) [111] to reduce false positives (Figure S34, S35). We collected contigs ≥ 5 kbp from Earth’s Microbiomes (GEM) catalog [35], Ocean Microbiomics Database (OMD) [12], and *Tara* Oceans project [52], which were not included in GOV2 (Figure S34) [58]. Only dsDNAphage, RNA and ssDNA phage (-include-groups “dsDNAphage,RNA,ssDNA”) were included in the Virsorter search to enrich the phage sequence. Contigs identified by VirSorter2 were pulled to calculate the BUSCO ratio (the percentage of hits to BUSCO, using BUSCO v5.3.2 against lineage datasets of bacteria_odb10 and archaea_odb10) [110] and the number of VPF hits (hmmsearch with evalue < 0.05), similar to the methods described previously [63]. Of these contigs, those with a BUSCO ratio < 0.067 or at least 3 VPF hits were classified as putative viral [63]. Otherwise, those with a VirFinder score > 0.9 and *p* < 0.05 were also kept. The retained contigs were then run through CAT (v.5.1.2) [101] and the classified kingdoms were rendered with different colors. The purple points (“Virus”) tended to be located in the bottom right region (Figure S34), revealing the performance of our method. We utilized CheckV [112] (v1.0.1, end-to-end mode with default parameters) to assess the quality of the identified viral sequences. As a control, 99.9% (excluding 132/488,131) of sequences from GOV2 dataset can be identified as viral sequences using our proposed methods. Most of the phages in GOV2 and this study are assigned as low-quality by CheckV (Figure S36). Similar results could be observed in the completeness-contamination plot (Figure S37), with most of the points located on the bottom left (low completeness and low contamination). To further reduce the false positives, the putative viral contigs with no hallmark genes predicted by VirSorter2 or geNomad [113], or with “Not-determined” completeness evaluated by CheckV were removed from the downstream analysis. The resulting 1,587,746 viral sequences identified in this study and 457,607 million from GOV2 were merged as the Ocean Virus Database (OVD). BBMap v.38.79 [88] was then used to remove sequences with 99% redundancy, resulting in a total of 1,647,134 viral sequences. We applied “virus operational taxonomic units” (vOTUs), proposed by MIUViG [114], to define species-rank viral groups with the parameter of 95% average nucleotide identity (ANI) and 85% alignment fraction (AF). In brief, all viral contigs were clustered into species-level vOTUs based on 95% ANI and 85% AF using pairwise ANI calculations from the CheckV repository’s rapid genome clustering supporting code (https://bitbucket.org/berkeleylab/checkv/src/master/). Firstly, all-vs-all local alignments were performed using BLASTN (v.2.12.0 +), with the parameters (-task megablast -max_target_seqs 10,000). Then, pairwise ANI values were calculated by combining local alignments between sequence pairs using anicalc.py script. Finally, UCLUST-like clustering was carried out with the aniclust.py script using the recommended-parameters (95% ANI + 85% AF) from MIUVIG.

### Taxonomic classification of viral sequences

Demovir (https://github.com/feargalr/Demovir, downloaded in February 2023, default parameters) and geNomad (v1.4.0, end-to-end mode with default parameters) [113] were used for the taxonomic classification of viral sequences. The contig assigned inconsistent results by different tools would be assigned the higher rank with consistent results. For example, the contig assigned as “Viruses; Duplodnaviria; Heunggongvirae; Uroviricota; Caudoviricetes; Myoviridae” and “Viruses; Duplodnaviria; Heunggongvirae; Uroviricota; Caudoviricetes” by Demovir and geNomad, respectively, was finally assigned as “Caudoviricetes”. If all ranks showed inconsistent results, the contig would be classified as “unassigned virus”. The contig that could not be assigned taxonomy by either tool would be classified as an “unassigned virus”. To unify the results from different tools, the International Committee on Taxonomy of Viruses (ICTV) lineages were used as a standard [115]. Therefore, the contig that was assigned the rank that ICTV no longer uses would be assigned the higher rank that ICTV still uses. For instance, “Myoviridae” is no longer used by ICTV; therefore, the contig mentioned above was assigned as “Caudoviricetes,” which is still used by ICTV. The viral lineages not associated with prokaryotes were removed from further analysis, such as the eukaryote infecting lineage “Viruses; Varidnaviria; Bamfordvirae; Nucleocytoviricota.”

### Viral gene calling

Due to the prevalence of stop codon reassignment in phage communities, Prodigal (v2.6.3, -p meta) was run using the standard code (11), *opal* code (25), *amber* code (15), and *ochre* code (90) [116, 117]. The *opal* code 25 (*opal* reassigned to Gly) and *amber* code 15 (*amber* reassigned to Gln) are the existing genetic codes of the Prodigal software (https://github.com/hyattpd/prodigal/wiki/Advice-by-Input-Type#alternate-genetic-codes), which were used to detect *opal* and *amber* reassignments, respectively. The modified version of Prodigal with *ochre* code 90 is available on the JGI public ftp site https://portal.nersc.gov/dna/microbial/prokpubs/recoding/. The output gbk file includes a coding potential score for each predicted gene, which could be used for the evaluation of genetic codes. Coding potential scores per contig were summed as the cumulative coding score of a contig. The alternative genetic code was used when the cumulative coding score of a contig was the highest and at least 20% greater than the standard genetic code.

### Viral protein annotations and clustering

Fourteen million six hundred sixty-six thousand seven hundred fifty-six nonredundant proteins were identified from 1,647,134 viral sequences and annotated based on HMM searches against protein family databases: KEGG (download: April 2022) [118], Pfam-A (v35) [119], and VOGDB (http://vogdb.org, March 2022). All searches were performed using the hmmsearch utility in the HMMER package v.3.3.1 with default parameters (http://hmmer.org/). Each gene was annotated by each database according to its top-scoring alignment with a bit-score ≥ 50, except for Pfam where trusted cutoffs were used. For the remote homology detection, all viral proteins were searched against PHROG, pdb70, CDD, COG_KOG, SCOPE70, and Uniprot_sprot_vir70 using HH-suite3 (v3.3.0) [100, 120–126]. The annotation was significant if evalue < 0.01 and the alignment length was at least 50% of the query and target sequence. The number of proteins annotated against different databases could be found in Figure S38.

Viral proteins were clustered into viral protein families using MCL (“-I 2.0”) [127] based on all-versus-all pairwise comparison using diamond [128] (v2.0.13.151; e-value < 0.0001). The viral protein family was labeled by the most abundant annotations of all members. The functional categories were classified based on PHROG. The “connector,” “tail,” and “head and packaging” categories in PHROG were combined as the “Structure” category. The “other” was merged into the “Unknown function.” Besides, the protein for which no homologs could be found in viral protein databases (PHROG, VOGDB, Uniprot_sprot_vir70) was labeled as “Unknown function.” In total, all viral protein families were classified into seven functional categories as shown in Fig. 5B.

### Metatranscriptomic profiling and co-expression analysis

Our objective was to explore the involvement of RiPPs in putative phage-host interactions. To achieve this, we calculated the abundance of RiPP families/protein families (≤ 50 kbp away from RiPPs)/viral protein families by aggregating the abundance of corresponding nonredundant genes in metatranscriptomes. To reduce the complexity of abundance inference, the nucleotide sequences (provided by “-d” option of prodigal-short) of the RiPP-encoding genes, genes within 50 kbp away from RiPPs, and viral genes were clustered separately into nonredundant gene clusters with 95% identity (-c 0.95) and 90% coverage (-aS 0.9) of the shorter gene using CD-HIT (v.4.8.1) [29]. The longest sequence was selected as the representative gene for each gene cluster. The metatranscriptomes were then mapped to the gene cluster representatives using LAST (v1418) [129], which is a local and general aligner similar to BLAST and Diamond, but it is much faster than the latter two. We opted to use LAST instead of kallisto-like tools such as bowtie2 [130] and BWA [131], which are mainly designed for mapping reads to genomes in most studies. We selected LAST as it is commonly used for mapping reads against genes [132]. The results were filtered to retain only alignments with a minimum alignment score $\ge$ 120 and a percentage identity of $\ge$ 95% and $\ge$ 45 bases aligned. The ambiguously mapped and single-end mapped (for paired sequencing data) inserts were removed from downstream analysis.

All reads mapped to members of a given protein family were added for metatranscriptomic profiles of protein families. Protein families’ metatranscriptomic profiles were normalized using the “varianceStabilizingTransformation” function of DESeq2 and then sent to WGCNA (v1.71) for co-expression analysis [48]. Metatranscriptomic reads detected in at least six samples were included in the analysis. After processing, the blockwiseModules function from the WGCNA library was called in R using default parameters except for networkType = “signed”, minModuleSize = 30, corType = “Bicor,” deepSplit = 3, maxBlockSize = 50,000. To best capture patterns of co-expression, a signed network was used. We used the pickSoftThreshold function from WGCNA to test values from 1 to 30 to pick an adequate power for each dataset. We empirically determined a soft threshold of 8 for the networks of RiPP families and protein families and 9 for the network of RiPP families and viral protein families in metatranscriptomes. Considering the influence of environmental factors, including temperature, depth, and latitude, on the transcriptions of RiPPs and viral genes (Figure S39, S40), module eigengenes were related back to environmental metadata using Pearson correlation, and the modules highly correlated with “Depth,” “Temperature,” and “Latitude” were removed from the subsequent analysis.

### Identification of phage-host relationships

Predicting the cellular hosts of phages is essential in understanding the associations among the RiPP families, hosts, and phages. Towards this goal, we leveraged the constructed databases OGD and OVD, containing 21,171 prokaryotic genomes/contigs and 1,894,072 viral contigs, respectively, as the host-phage pairwise searching pool. We first used the CRISPR-Cas prokaryotic immune system, which recorded the phage infection history on the host genomes [133]. The infection records in the form of spacers can be matched to phage genomes and build connections with their hosts [134]. We amassed 151,364 CRISPR spacers from OGD using CRISPRCasFinder (v4.2.18, default parameters) [135] and looked for near-exact matches ($\le$ 2 bases difference) to 1,894,072 viral contigs using BLASTN (v2.12.0 + , -task blastn-short -evalue 1e-5 -word_size 7) [77], resulting in 3816 host-phage pairs (Figure S27). Based on the hypothesis that viral transfer RNA (tRNA) genes originate from their hosts [136], we also extracted 407,425 host tRNAs and 152,464 viral tRNAs from the searching pool using tRNAscan-SE (v2.0.9; using the bacterial/archeal and general models, respectively) [137]. To remove the promiscuous viral tRNAs (the viral tRNA sequence that highly conserved across bacteria) from the identified viral tRNAs, all-vs-all local alignments against the “Promiscuous viral tRNA sequences” established by David Paez-Espino et al. [57] were performed using BLASTN (v.2.12.0 +), with the parameters (-task blastn, -evalue 1e-5). The near-exact matches ($\le$ 2 bases difference) between host and viral tRNAs were queried using BLASTN (v2.12.0 + , -task blastn -evalue 1e-5), detecting 298,269 host-phage pairs (Figure S27). To expand the host-phage network, we performed contig-to-contig alignments between the OGD and OVD using BLASTN (v2.12.0 + , -task megablast) (Figure S27). A microbial sequence with ≥ 2500bp regions of their sequence matching at ≥ 70% identity with a viral sequence were kept for further consideration. These matches were then further filtered by both viral contig coverage (requiring at least 30% viral coverage) and host contig coverage (requiring at least 30% of the host contig to be outside the prophage region alignment), resulting in the identification of 192,508 host-phage connections. The details of the principles used to identify host-phage relationships could be found in Supplementary Table 7.

### Supplementary Information

**Additional file 1. **Supplementary Information, Supplementary Figure S1-41, Supplementary Table 1-7.

**Additional file 2. **Supplementary Data 1-12.

**Additional file 3. **Supplementary Data 13-22.

## Data Availability

The metagenome-assembled genomes (MAGs), single amplified genomes (SAGs), and unbinned contigs are amassed from JGI-IMG/M database (https://img.jgi.doe.gov/), European Nucleotide Archive (https://www.ebi.ac.uk/ena/), http://www.genoscope.cns.fr/tara/ and https://portal.nersc.gov/GEM, accession numbers and links are included as Supplementary Data 11 in Additional file 2. The OTUs from the GEM database (marine, engineered, host-associated, non-marine-aquatic, and terrestrial) are downloaded from https://portal.nersc.gov/GEM/ and details are included as Supplementary Data 13 in Additional file 3. The isolated marine microbial genomes are selected from the MARREF (v1.6) and MARDB (v1.5) databases (https://mmp2.sfb.uit.no/databases/) and downloaded from NCBI Assembly RefSeq database (https://ftp.ncbi.nlm.nih.gov/genomes/refseq/) and Genbank database (https://ftp.ncbi.nlm.nih.gov/genomes/genbank/), accession numbers are included as Supplementary Data 14 in Additional file 3. The historical assembly summaries in NCBI were downloaded from https://ftp.ncbi.nlm.nih.gov/genomes/genbank/bacteria/assembly_summary_historical.txt for GenBank entries and https://ftp.ncbi.nlm.nih.gov/genomes/refseq/bacteria/assembly_summary_historical.txt for RefSeq entries. The accessions of the collected marine metatranscriptomes are included as Supplementary Data 15 in Additional file 3. The generated OVD database and the established prokaryote-phage interaction network in this study are available in the Zenodo repository: https://zenodo.org/records/10901853. The code used for the analyses performed in this study is accessible at GitHub (https://github.com/GAOYingHKU/OceanRiPPs). Source code for the TrRiPP model, training data, trained weight and inference script are available at https://github.com/zzhongzz/TrRiPP.
