# Supplementary material for: Exploring the roles of ribosomal peptides in prokaryote-phage interactions through deep learning-enabled metagenome mining
Source: Microbiome. 2024 May 24;12:94. doi: 10.1186/s40168-024-01807-y (PMC11118758; doi:10.1186/s40168-024-01807-y)
Supplement: Supplementary file 2 — Additional file 1. Supplementary Information, Supplementary Figure S1-41, Supplementary Table 1-7. [file 40168_2024_1807_MOESM1_ESM.docx]

Exploring the Roles of Ribosomal Peptides in Prokaryote-Phage Interactions through Deep Learning-Enabled Metagenome Mining

Ying Gao^1*^ , Zheng Zhong^1*^, Dengwei Zhang^1^, Jian Zhang^1^, and Yong-Xin Li^1#^

^1^ Department of Chemistry and The Swire Institute of Marine Science, The University of Hong Kong, Pokfulam Road, Hong Kong, China

^*^ These authors contributed equally to this work.

^#^To whom correspondence may be addressed.

Email: [yxpli@hku.hk](mailto:yxpli@hku.hk),

**Supplementary Information**

***The confidence level of TrRiPP-predicted RiPP families***

In accordance with the confidence assignment principle outlined in the **Methods** section, approximately 26% (2,202) of RiPP families from metagenomes were assigned the label of "high confidence". The low rate of "high confidence" in metagenomes is primarily attributed to the issue of fragmentation, with only one gene or 1-kbp region frequently found in the proximity to the RiPP family (~25%, **Figure S41B**). Due to the presence of incomplete flanking information and the possibility of discrete biosynthetic patterns, it is inevitable to omit the core post-translational modifications (PTMs). Furthermore, deep learning methods, which solely focus on capturing the features of precursor sequences, exhibit a higher tolerance for novel PTMs compared to domain-based PTM searching algorithms, such as antiSMASH [1]. This characteristic allows deep learning methods to identify and analyze PTMs that are not yet documented or understood in the existing body of knowledge.

***Comparison of TrRiPP-predicted RiPP families from Ocean metagenomes with publicly available databases***

We compared 8,354 RiPP families from ocean metagenomes with those from public databases (**Figure S13, Supplementary Data 19**): (1) RiPP precursors with high confidence predicted by TrRiPP from RefSeq bacterial genomes [2] (RefSeq REF), (2) RiPP precursors extracted from OMD [3] (predicted by antiSMASH), (3) RiPP precursors extracted from BIG-FAM [4] (predicted by antiSMASH) and (4) experimentally validated RiPPs from MIBiG [5]. RiPP families from ocean metagenomes (**Figure S13**, up and bottom row) showed similar identity distribution patterns with RiPPs from different public databases between the high- and low-confidence group, except for a slight shift towards higher identity in the high-confidence group (**Figure S13**, "RefSeq REF" and "BIG-FAM", mainly ranging from 60% to 100%). When compared with RiPPs from OMD [3], which was a recently published Ocean Microbiomics Database and contributed a large portion of microbial genomic data in this study (**Supplementary Data 19**), more than 85% of (1880/2202) RiPP families from ocean metagenomes with high confidence showed medium-to-low (≤60%) similarity with OMD, and around 26% (486/1880) of them could not find homologs in the OMD (**Figure S13**, "OMD"). This revealed the great performance of TrRiPP in mining RiPPs from metagenomic assemblies compared with antiSMASH [1], which only detected 1031 non-redundant RiPP precursor sequences (100% identity) from OMD. When compared with RiPPs from MIBiG (**Figure S13**, "MIBiG"), 56% (4704/8354) RiPP families from ocean metagenomes didn’t have any homologs in MIBiG, elucidating the great potential of discovering novel RiPPs from ocean microbiome.

***RiPP-encoding BGCs from Kordia***

We illustrated RiPP-encoding BGCs from Kordia by BiG-SCAPE [6] using the default parameters. The TrRiPP-predicted RiPP subclasses were dominant by rSAM-modified peptide and lanthipeptide. For the BGCs containing class-defining PTMs, we found some putative novel biosynthetic modules. For instance, LanC cyclase and Nucleotidyltransferase co-localized with the TrRiPP-predicted RiPP precursor (**Figure 3B**, Lan6-10). LanC cyclase is the core modification enzyme of lanthipeptide [7], while Nucleotidyltransferase was proposed to catalyze the glycosylation of the phosphorylated lasso peptide pseudomycoidin [8, 9]. Additionally, The bioinformatic analysis also reported the Nucleotidyltransferase-containing RaS-RiPP BGCs in *Parabacteroidetes* [10]. Therefore, LanC cyclase and Nucleotidyltransferase are probably to synergistically catalyze the RiPP precursor via a novel modification mechanism. The second attractive case is that multiple Subtilase-related Peptidase S8 and ABC transporter co-localize with the RiPP precursor (**Figure S19**, ABC5,12,28). Subtilase-related Peptidase S8 (PF00082) was reported to show serine-type peptidase activity. The well-known modification enzyme PatA/PatG of cyanobactin belongs to this peptidase family [11, 12]. In this study, multiple Subtilase-related Peptidases probably modify the RiPP precursors by themselves or assist other unknown enzymes. In addition, we also found several putative novel BGCs without class-defining PTMs. They contain enzymes or transporter associated with the biosynthesis of RiPPs, such as ABC transporter, Peptidase C39, Prolyl oligopeptidase, HylD, and Cupin-like domain (**Figure S19**), indicating their great potential for encoding novel PTMs.

***Normalization factors for the comparison of different ecosystems***

We normalized the RiPP-encoding OTUs by the average length of scaffolds (measured as the average number of bp from sequences) per habitat type (**Figure S21B**). The normalization factor used in each habitat type was: marine, 12.62kbp; non-marine-aquatic, 12.44kbp; terrestrial, 13.83kbp; host-associated, 20.01kbp; engineered, 19.35kbp.

**Supplementary Figures**


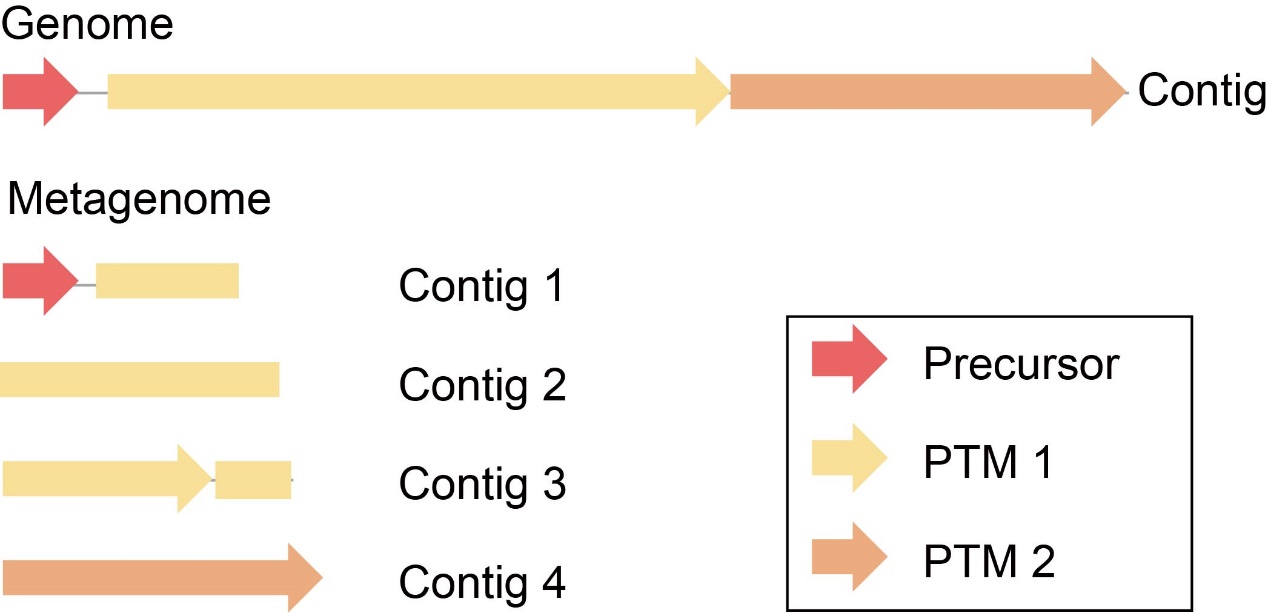


**Figure S1. RiPP BGC in genome and fragmented metagenome*.*** Due to their larger lengths, PTM enzymes are more likely to be fragmented in metagenomes. This makes RiPP BGCs more difficult to identify with traditional tools such as antiSMASH, which rely on annotation of PTM enzymes.


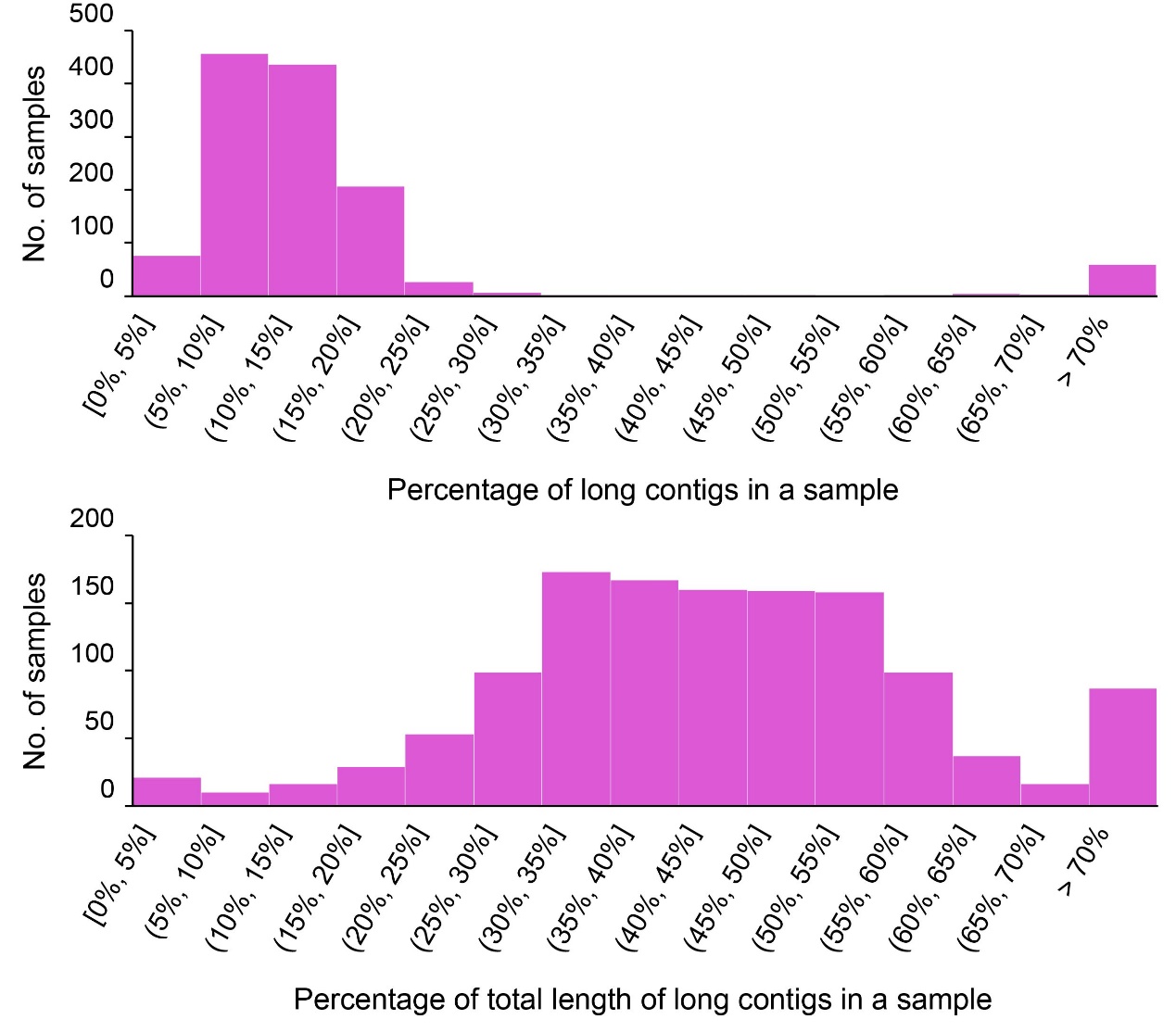


**Figure S2. Contig length distribution of metagenomic samples. A**: Histogram illustrating the percentage of long contigs (contigs ≥1000 bp) in a sample. The percentage shown on the x-axis means the number of long contigs over the number of all contigs. The majority of samples contain only <20% of long contigs. The mean value (weighted by the number of contigs in the samples) is 10.14%. **B**: Histogram illustrating the percentage of the total length of long contigs in a sample. The percentage shown on the x-axis means the total length of long contigs over the total length of all contigs. Most samples have long contigs contributing to 20%-60% of their total sequenced base pairs. The mean value (weighted by the total sequenced base pairs in the samples) is 40.32%.

**
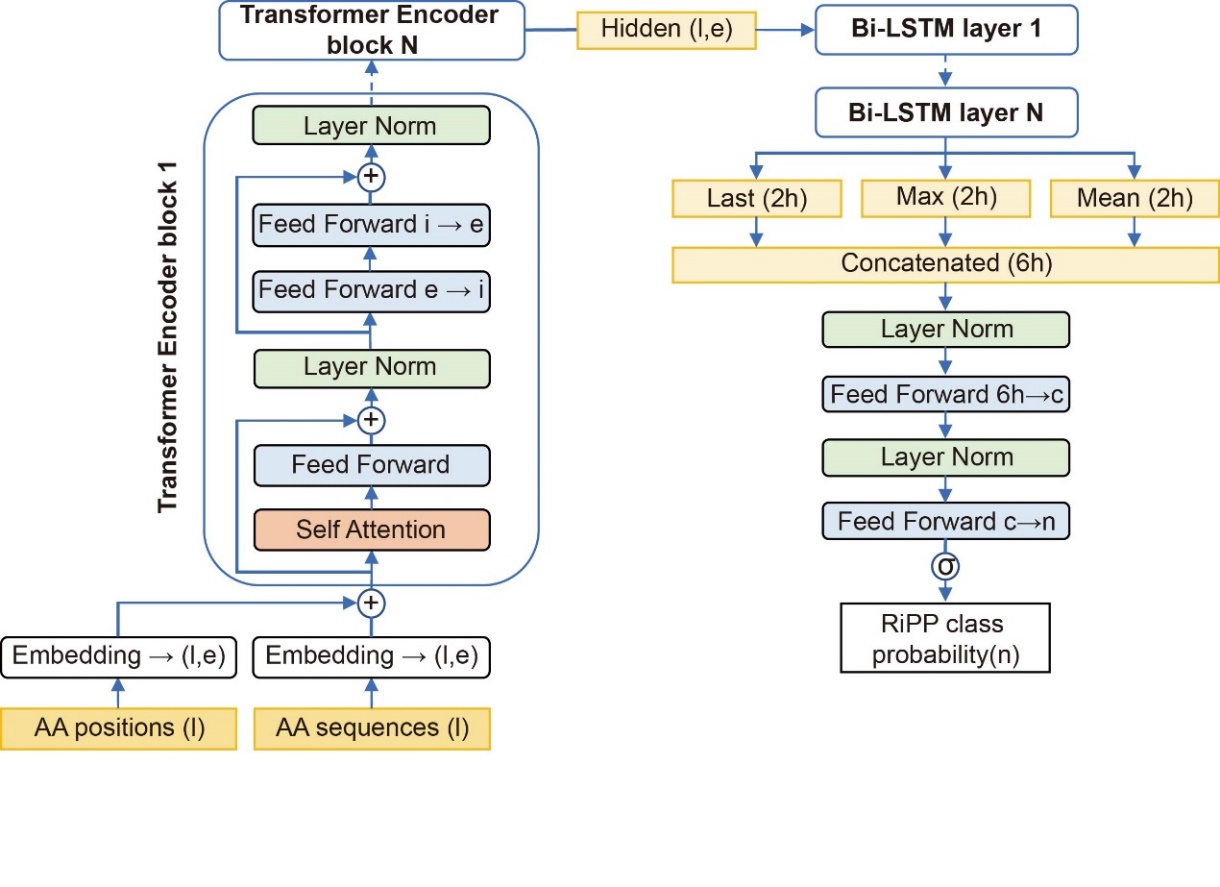
**

**Figure S3. Detailed model architecture of TrRiPP*.*** Amino acid sequence of length l is first converted into word embeddings (features of each amino acid) and positional embeddings (features of the position of each residue) by embedding layers, these two embeddings are summed and used as input of the transformer encoder. Each transformer encoder block contains a self-attention layer and several feedforward layers. Two residual connections present in each encoder block, each followed by a layer normalization. Multiple Transformer encoder blocks (N=4) are stacked together as the Transformer Encoder. The output from the Transformer encoder are then fed to multiple Bi-LSTM layers (N=2). Bi-LSTM layers process sequences in both forward and backward order and outputs the hidden state for each amino acid residue. These hidden states are pooled by concatenating the final, the maximum, and the mean values along the sequence length dimension and sent to a fully connected network with 2 layers, which reduces the dimension of hidden states from 6h (6x LSTM hidden size) to n (number of classes). The model then takes softmax of the final hidden state, and the output is used to calculate the loss against labels. l: sequence length, e: embedding size = 128, i: intermediate hidden size =256, h: LSTM hidden size =64, c: classification hidden size =192, n: number of classes = 10.


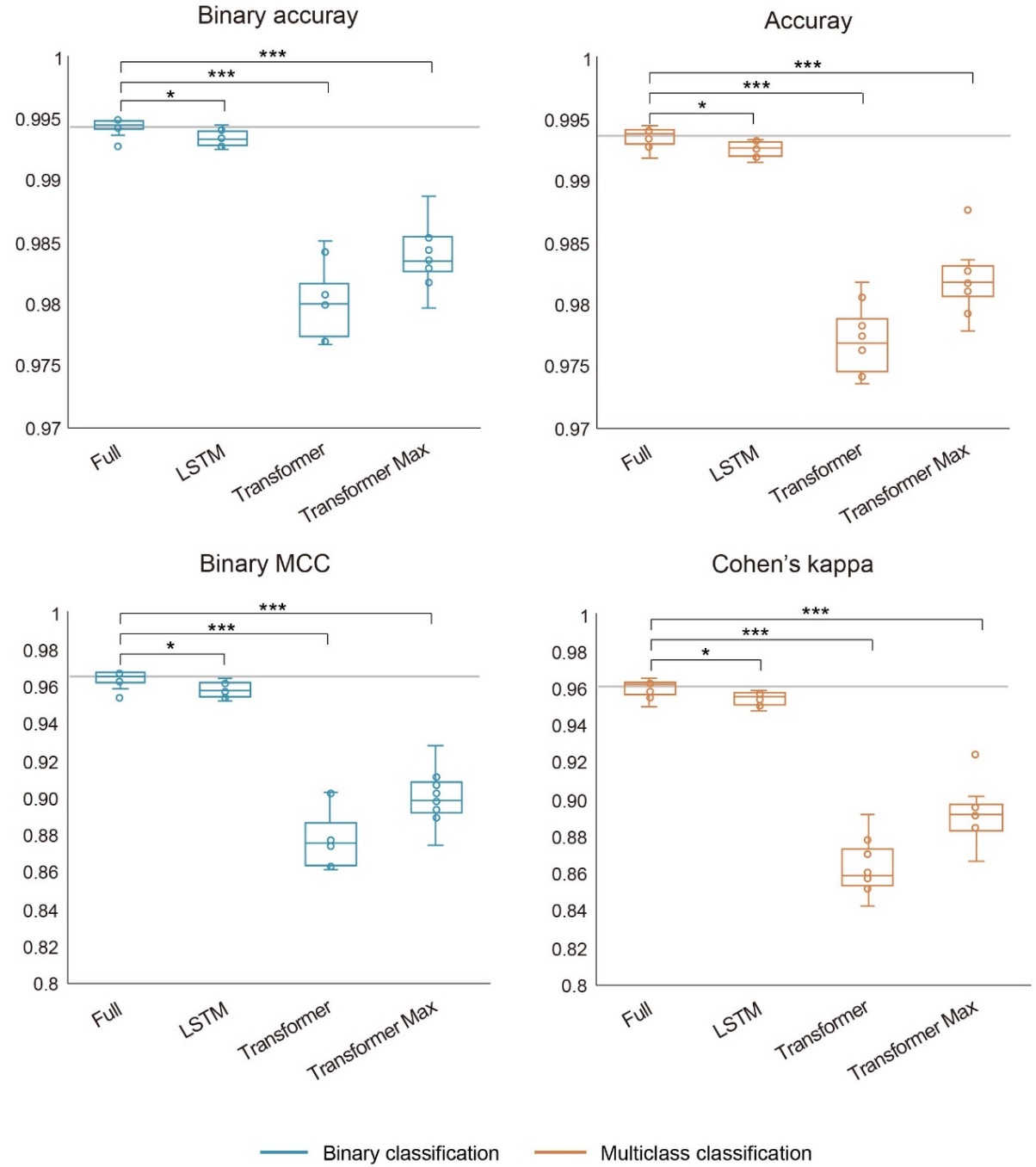


**Figure S4. Ablation study of TrRiPP.** Ablation results on the test set for two tasks: RiPP vs. non-RiPP peptide (binary) and RiPP classification (multiclass). Ablations are compared to the full model and reported as the average of the 10-fold cross-validation results. In each box, the center bar represents the median, the box represents the upper and lower quartiles, and the whiskers represent 1.5 times the interquartile range. Gray lines indicate the median of the full model. P-values are calculated by two-tailed Student’s t-test. *: *p*<0.05. ***: *p*<0.0005.


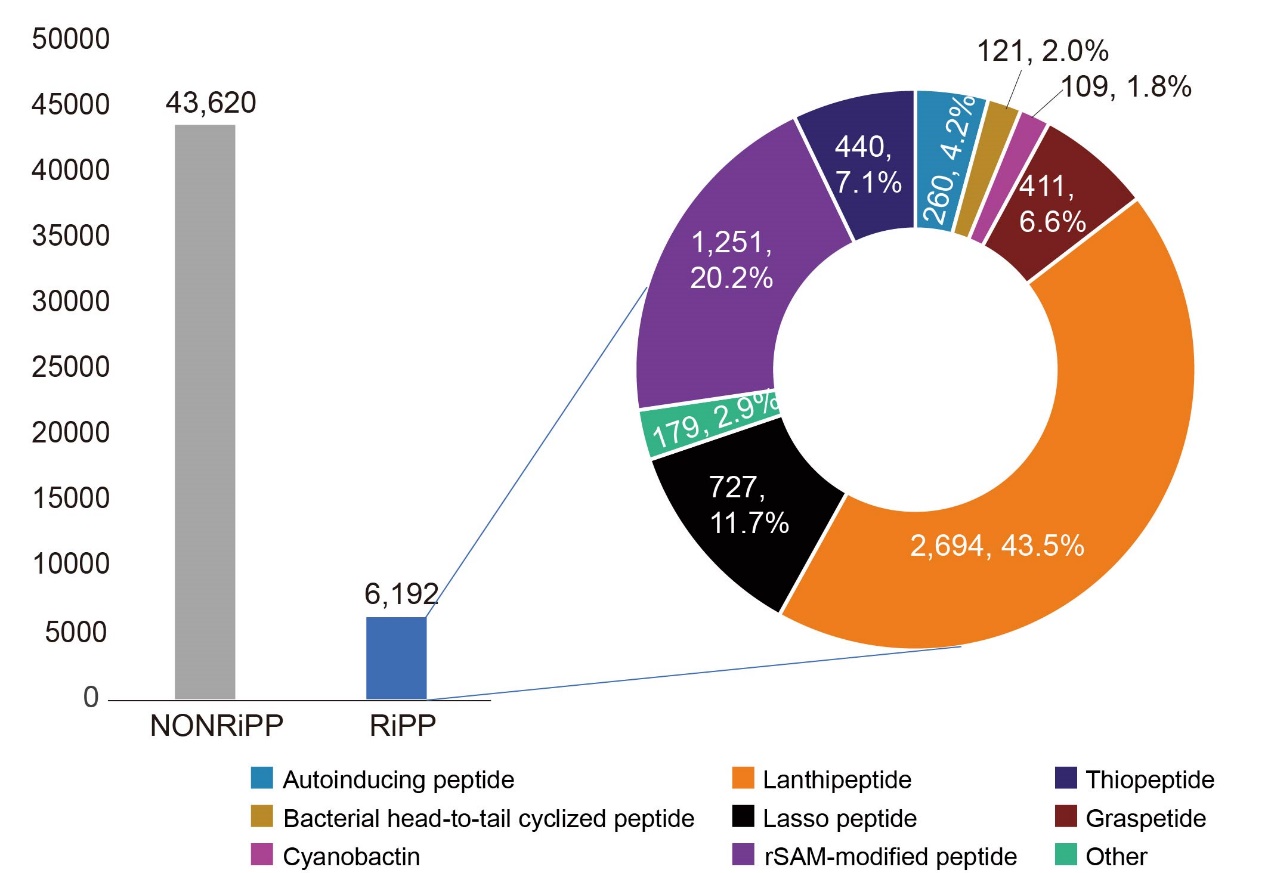


**Figure S5. Composition of training data.** Left: Number of RiPP and non-RiPP sequences in the final dataset. Right: Distribution of RiPP classes in the final dataset. The number and percentage of sequences in each class are shown.


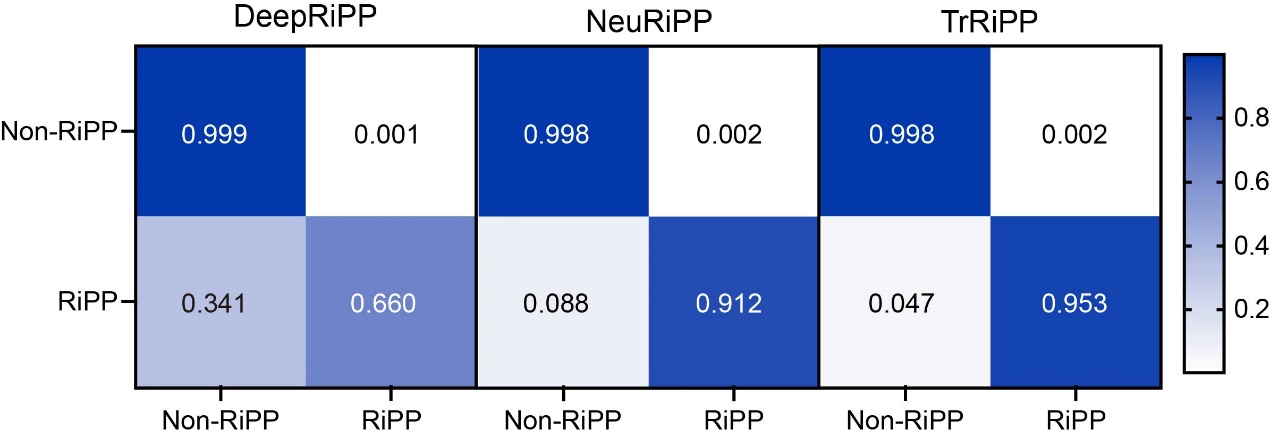


**Figure S6. Confusion matrix of TrRiPP, DeepRiPP, and NeuRiPP in binary prediction (RiPP vs. non-RiPP).** Confusion matrices depict the sum of the results of a 10-fold cross-validation (**Supplementary Data 20**). The x-axis is the predicted labels, while the y-axis is the true labels. Values in the diagonal indicate the true positive rate of RiPP and non-RiPP.


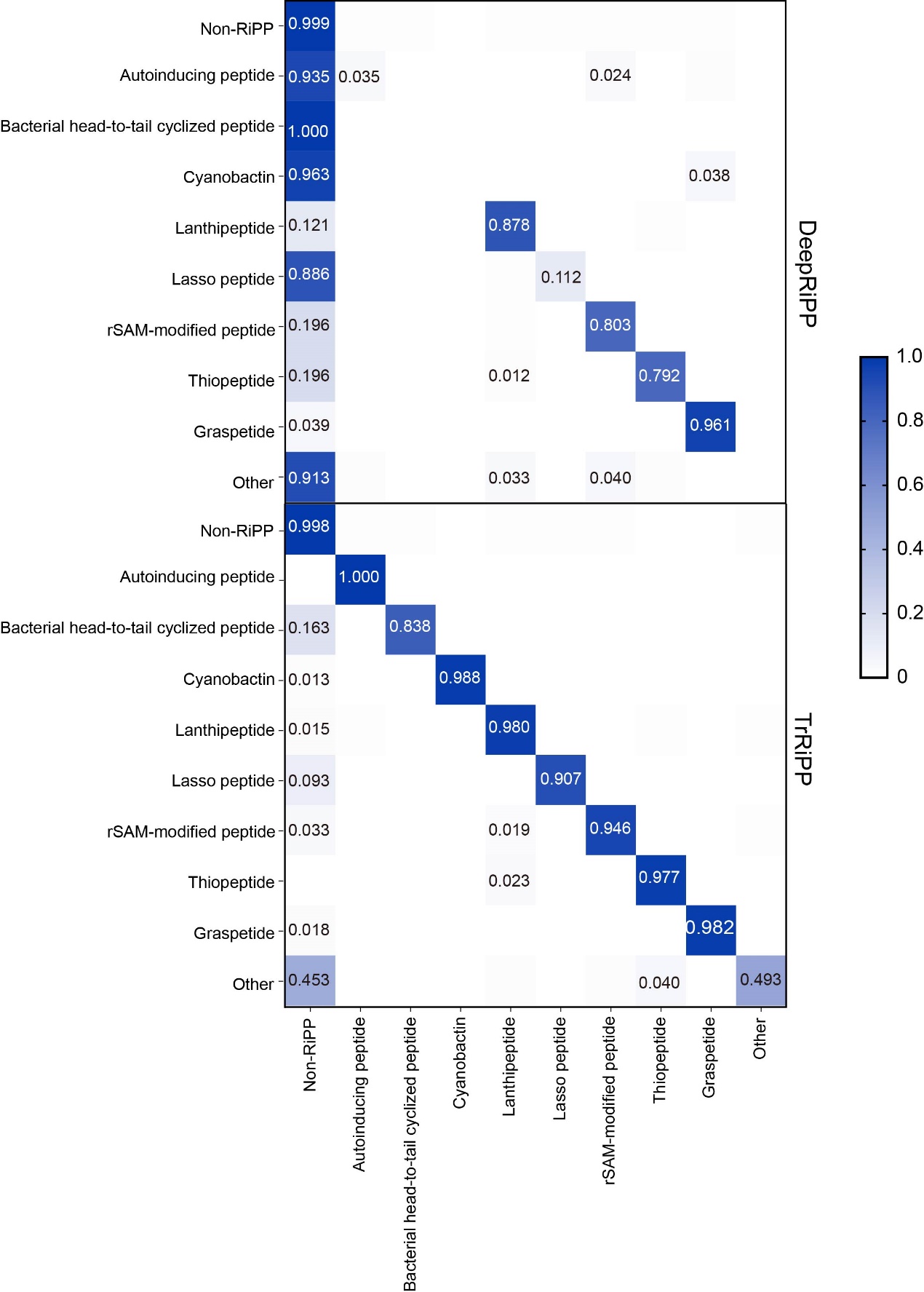


**Figure S7. Confusion matrix of TrRiPP and DeepRiPP in multiclass prediction (RiPP classification).** Confusion matrices depict the sum of results of a 10-fold cross-validation (**Supplementary Data 20**). The x-axis is the predicted labels, while the y-axis is the true labels. Values (>0.01) in the diagonal indicate the true positive rate of each RiPP family.

**
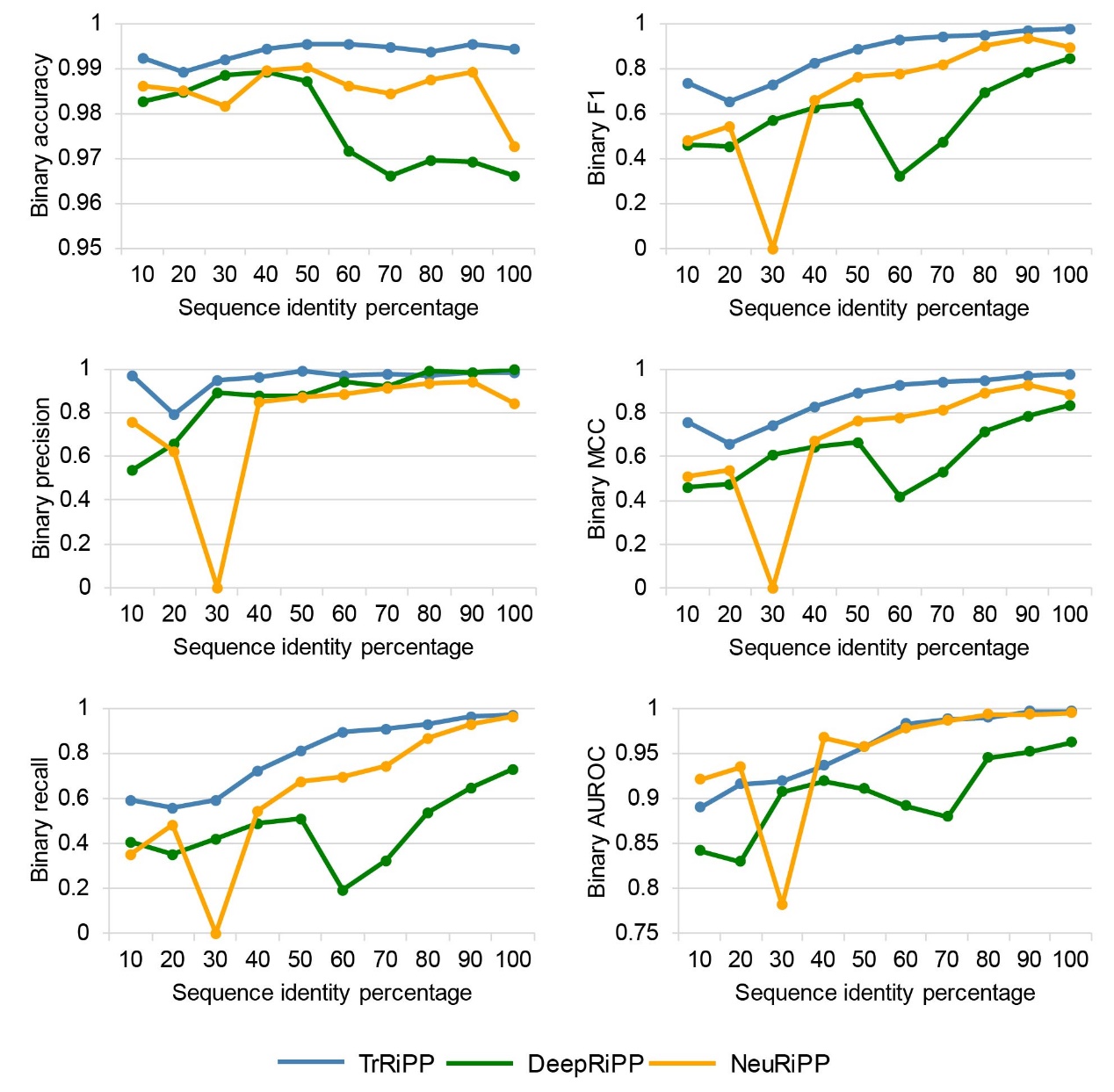
**

**Figure S8. Model generalization capabilities for binary prediction task.** The models were evaluated based on their ability to distinguish between RiPP precursors and non-RiPP peptides. The x-axis indicates the maximum identity between the test and training sets' sequences, while the y-axis displays the corresponding evaluation metrics.


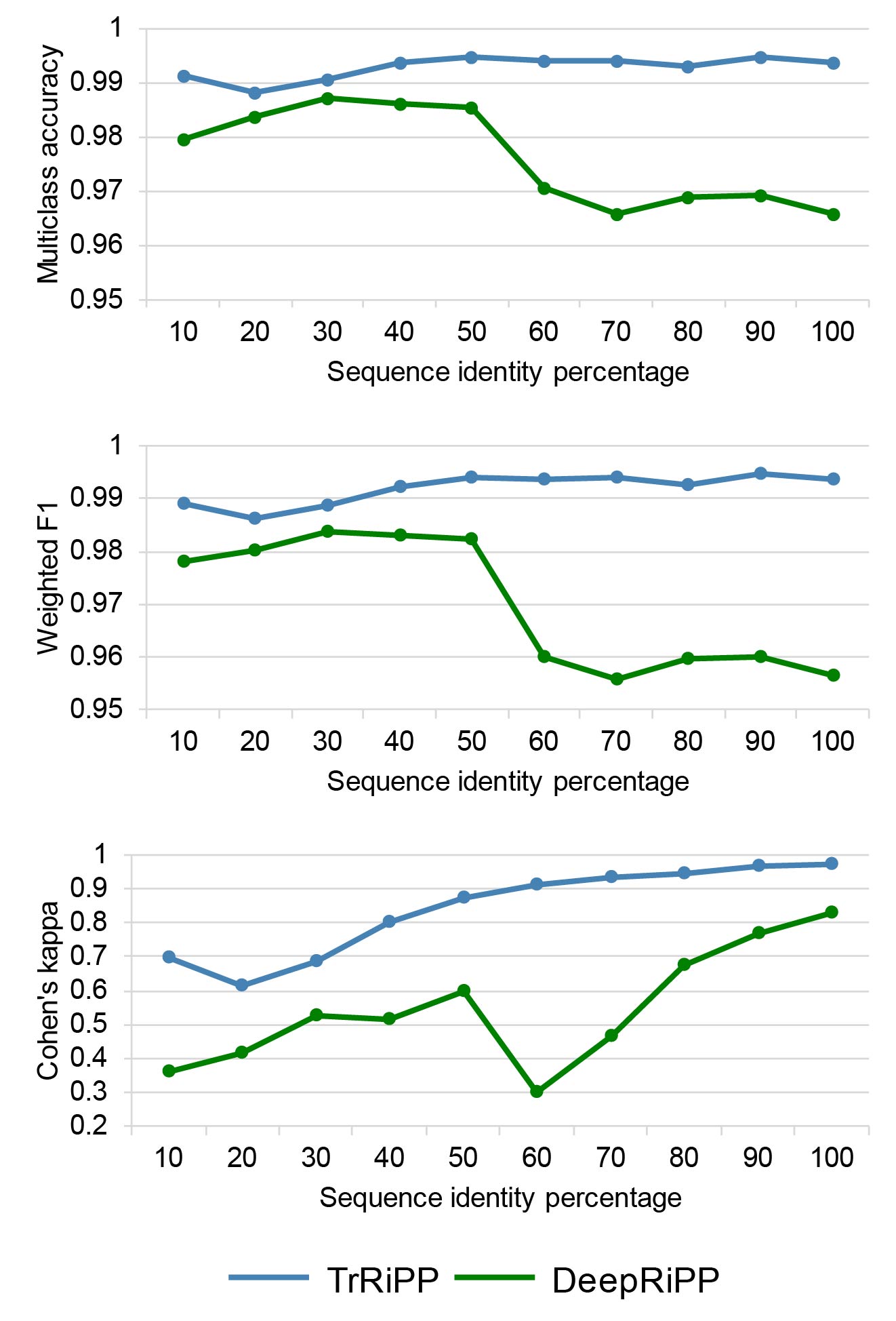


**Figure S9. Model generalization capabilities for multiclass classification task.** The models were evaluated based on their ability to classify RiPP precursors. The x-axis represents the maximum identity between test and training sequences, while the y-axis shows evaluation metrics.


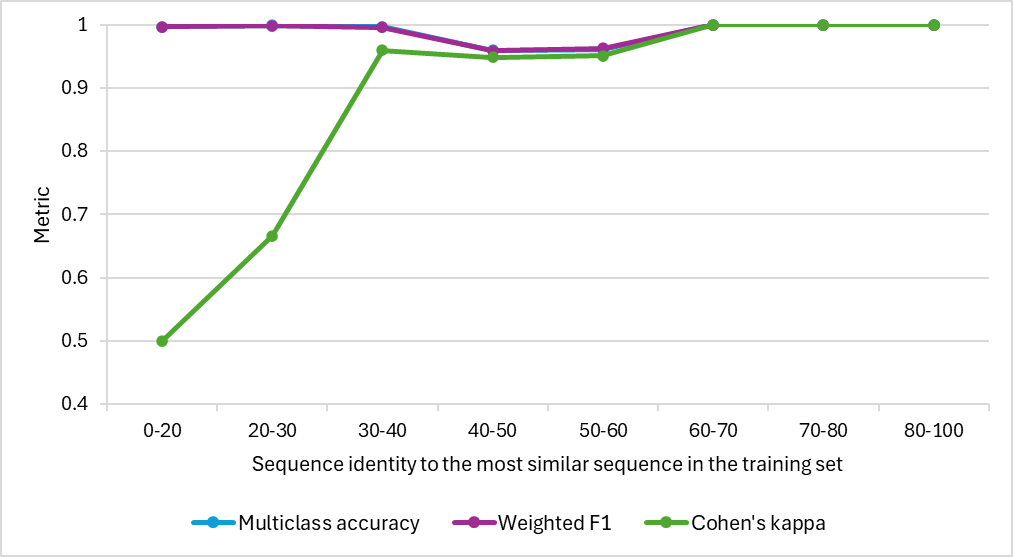


**Figure S10. Generalization capability of the final model** **provided with TrRiPP.** The x-axis represents the identity range between test and training sequences, while the y-axis shows evaluation metrics.


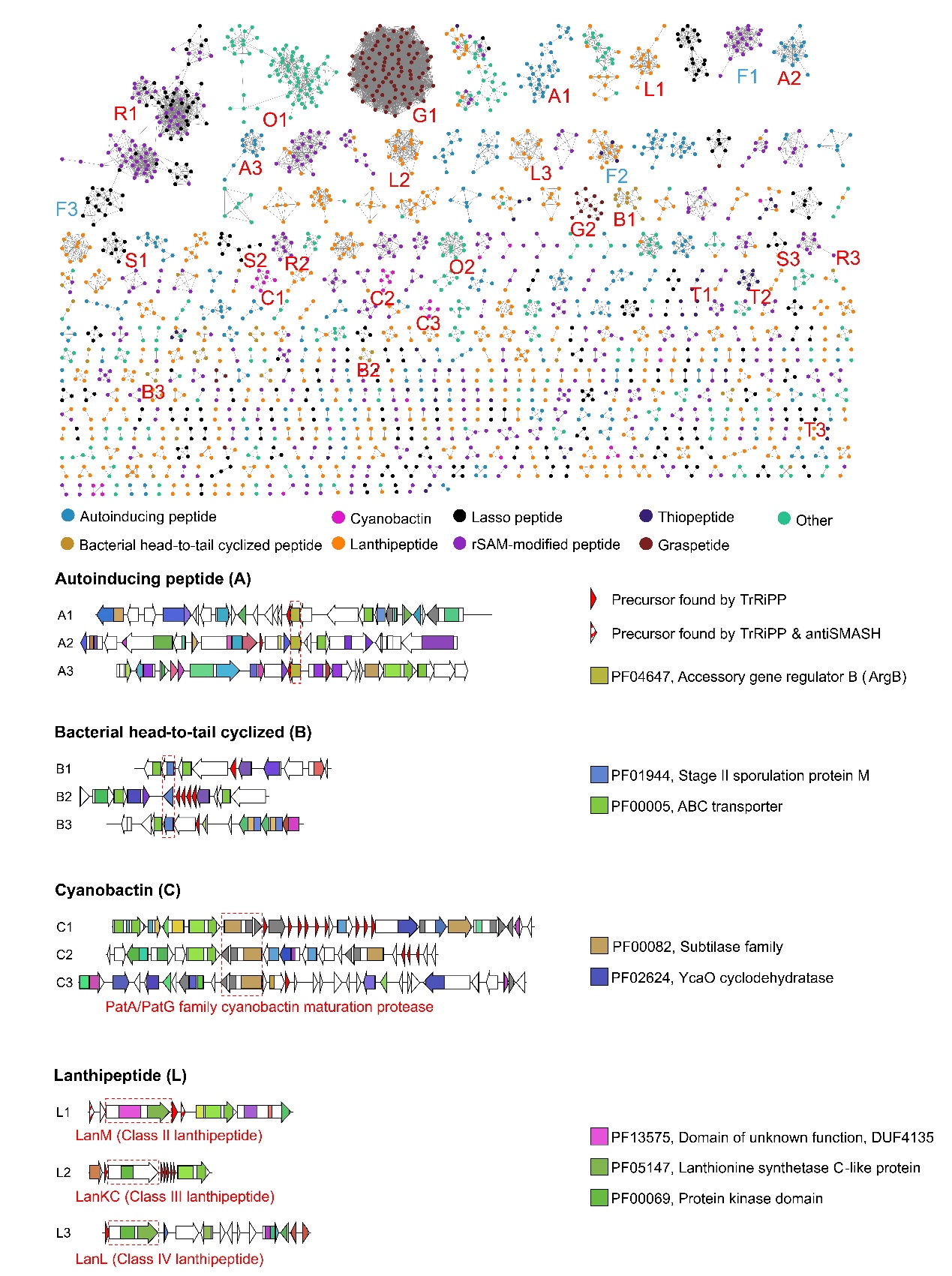


**Figure S11. Validation of high confidence predicted precursors via BGC visualization.** Top: Sequence similarity network of predicted RiPPs in “PTM” and “RiPP-related” validity levels (**Figure 1E**) from bacterial complete genomes. Each node represents a unique precursor. Selected clusters are labeled, where red labels are correctly predicted RiPPs, and blue labels are incorrectly predicted RiPPs. Bottom: Representative BGCs from selected RiPP precursor clusters. Key PTM enzymes are highlighted with red dashed lines. Important Pfam domains of each RiPP class are shown on the right.


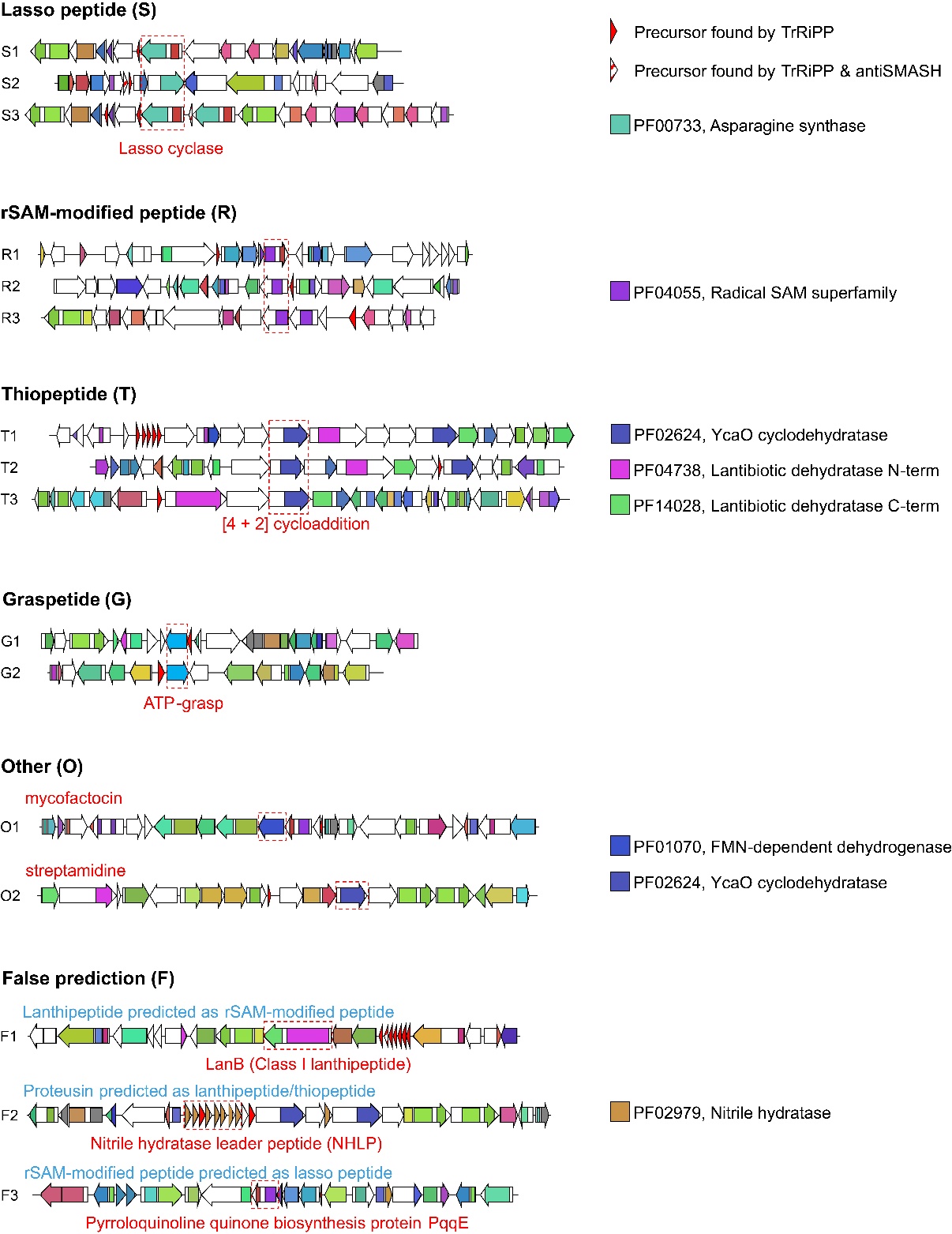


**Figure S11. Validation of high confidence predicted precursors via BGC visualization (continued).**


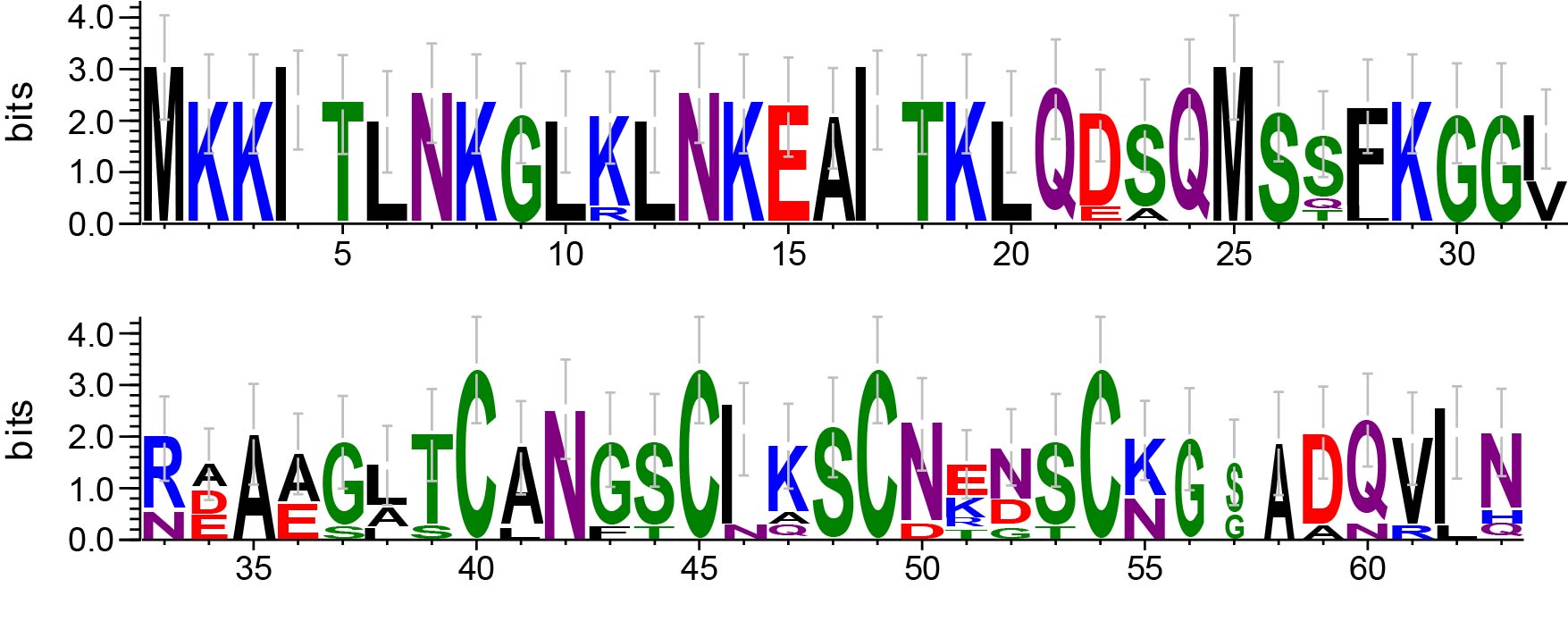


**Figure S12. Sequence logo of predicted precursors from cluster F1.** Sequences of the predicted precursors from cluster F1 in Figure S11 were used to create the sequence logo using Weblogo v3.7.4 (<http://weblogo.threeplusone.com>). Error bars indicate sample correction, and the total height of the error bar is twice this correction. Four cysteine residues show a highly conserved pattern in the C-terminus, which is a common feature of rSAM-modified peptides. We hypothesize that this is the reason why TrRiPP misclassified them as rSAM-modified peptides.


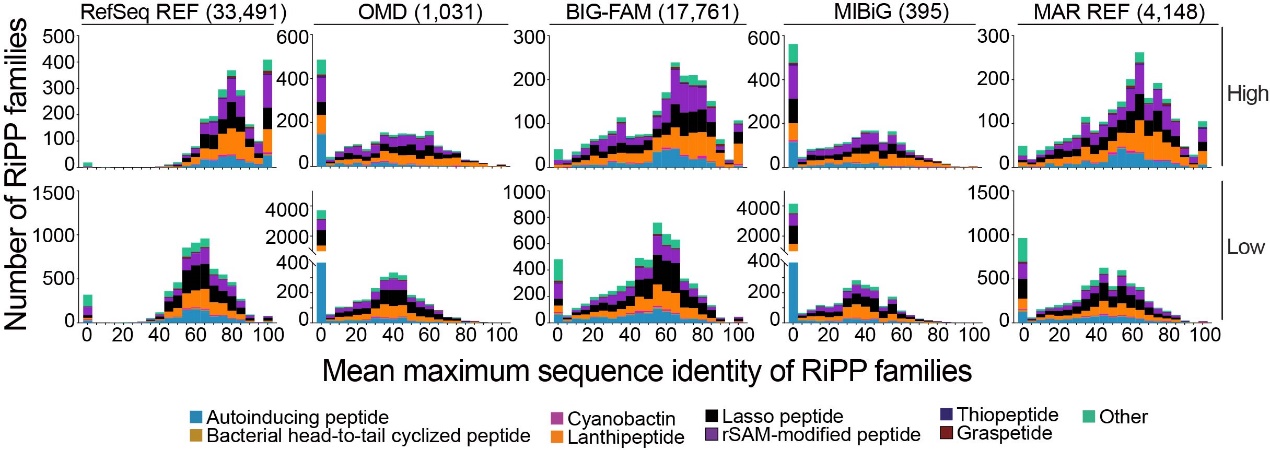


**Figure S13. Comparison of TrRiPP-predicted RiPP families from Ocean metagenome to reference RiPP precursor database, including “OMD”, “BiG-FAM”, “RefSeq”(TrRiPP-predicted RiPPs from RefSeq bacterial genomes with high confidence) and “MIBiG” (experimentally validated RiPPs).** The x-axis represents the average of the maximum sequence identity of the RiPP family against sequences in the reference database. The y-axis shows the distribution of the mean maximum sequence identity of RiPP families against different reference databases. The two rows represent the RiPP families from ocean metagenome with high and low confidence, respectively. The columns represent the reference databases, and the values in the brackets are the numbers of nonredundant RiPP precursor sequences from corresponding databases.


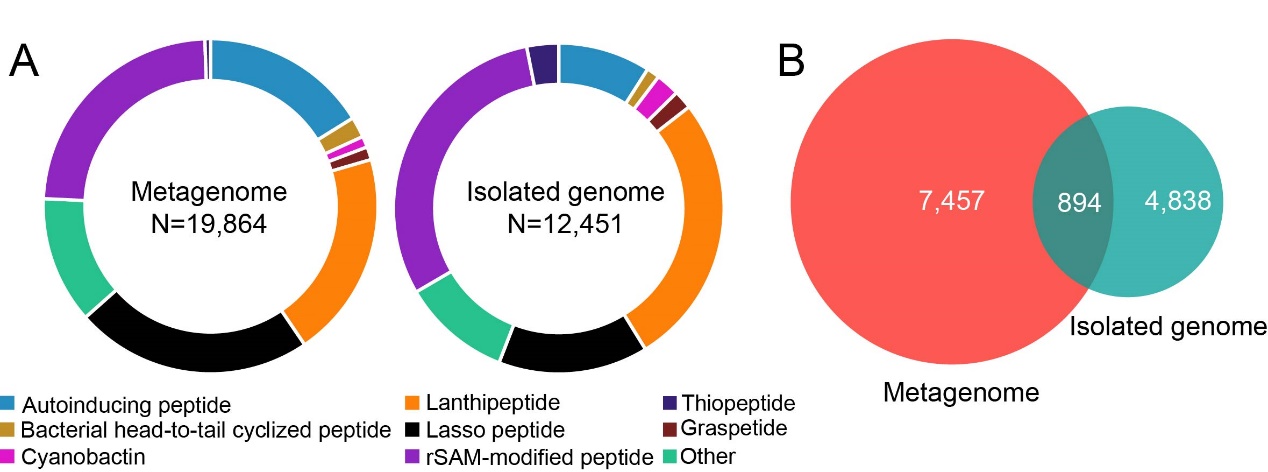


**Figure S14. RiPPs from marine metagenomes and isolated genomes. A.** Class distribution of predicted RiPPs from self-curated marine metagenome database and 13,050 isolated marine bacterial genomes. **B.** Venn diagram of predicted RiPP precursors in metagenomes and 13,050 isolated genomes. The number represents RiPP clusters with 60% sequence similarity.


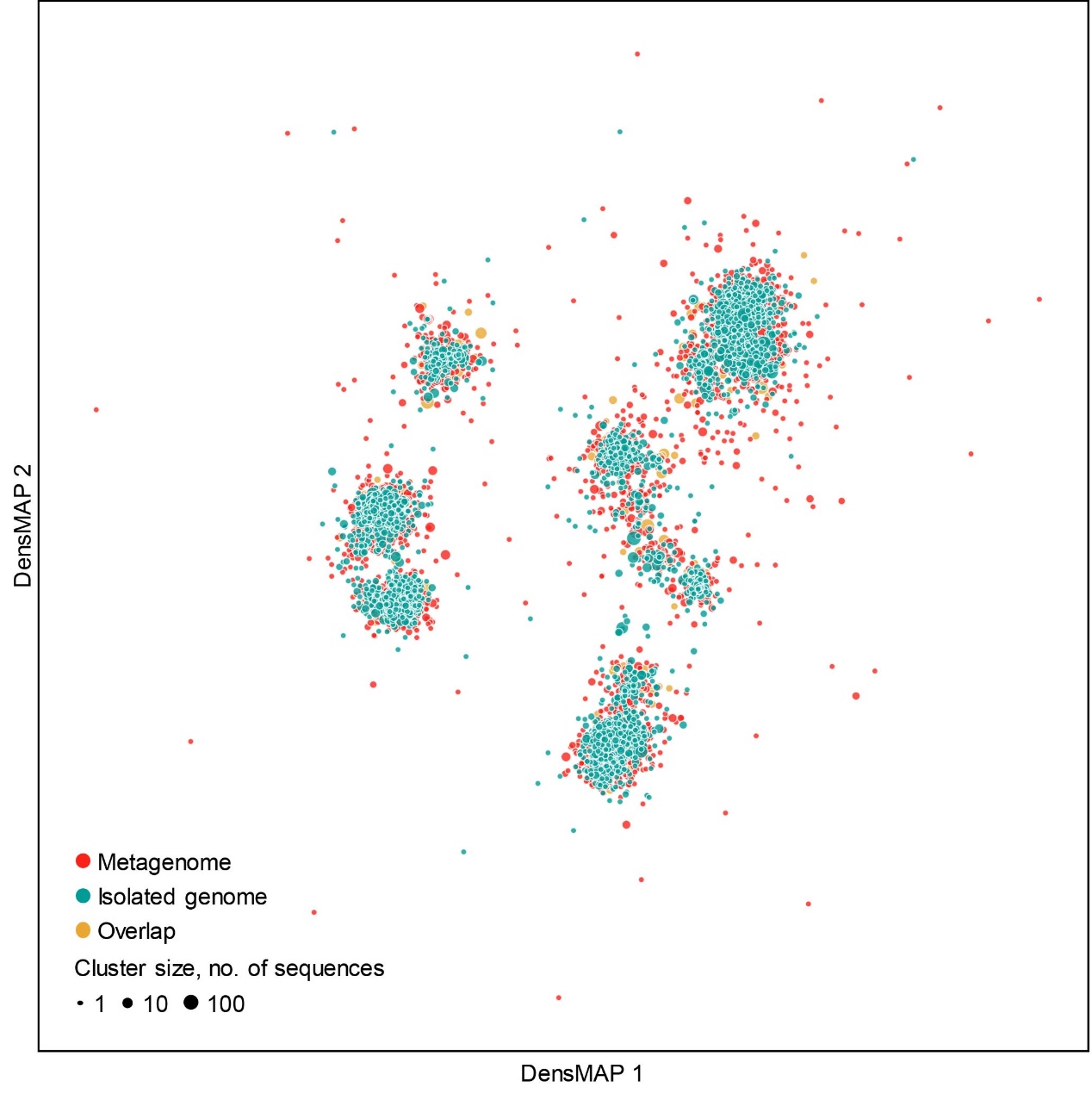


**Figure S15. Comparison of chemical space of RiPPs from marine metagenomes and isolated genomes.** Chemical space is visualized by the DensMAP plot of ECFP6 chemical fingerprints of RiPP clusters. Each node represents a RiPP cluster that is clustered with 60% sequence identity and coverage. Node size indicates the number of sequences in that cluster.


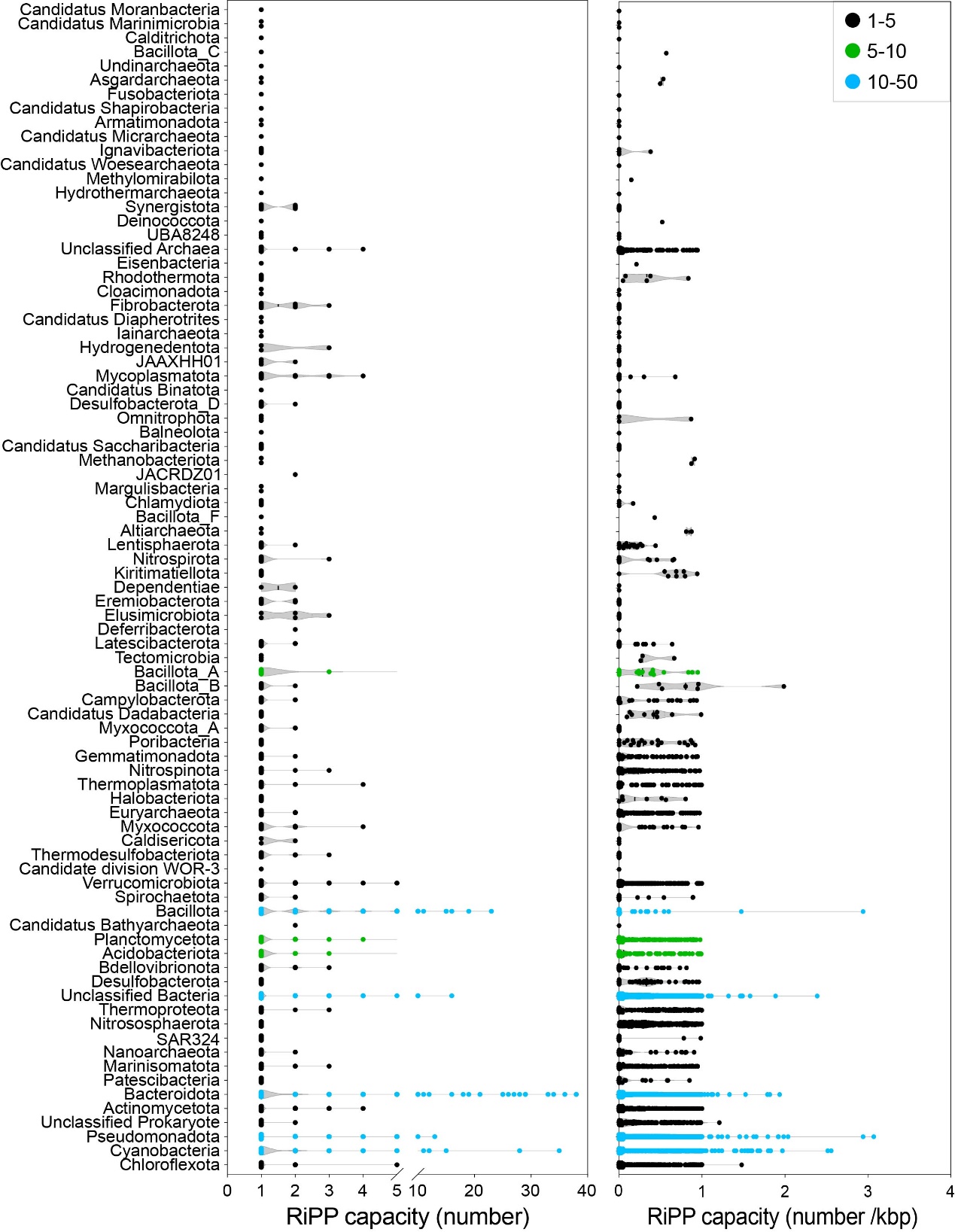


**Figure S16. The RiPP capacity of prokaryotic phylum from global ocean metagenomes.** The figure shows the RiPP capacity defined as the number of RiPPs encoded in the genomes/scaffolds (left) and the number of RiPPs encoded in the genomes/scaffolds per kilobase pair (right). The y-axis represents the prokaryotic phylum, and the color denotes the number of RiPPs. Source data can be found in **Supplementary Data 21, 22**.


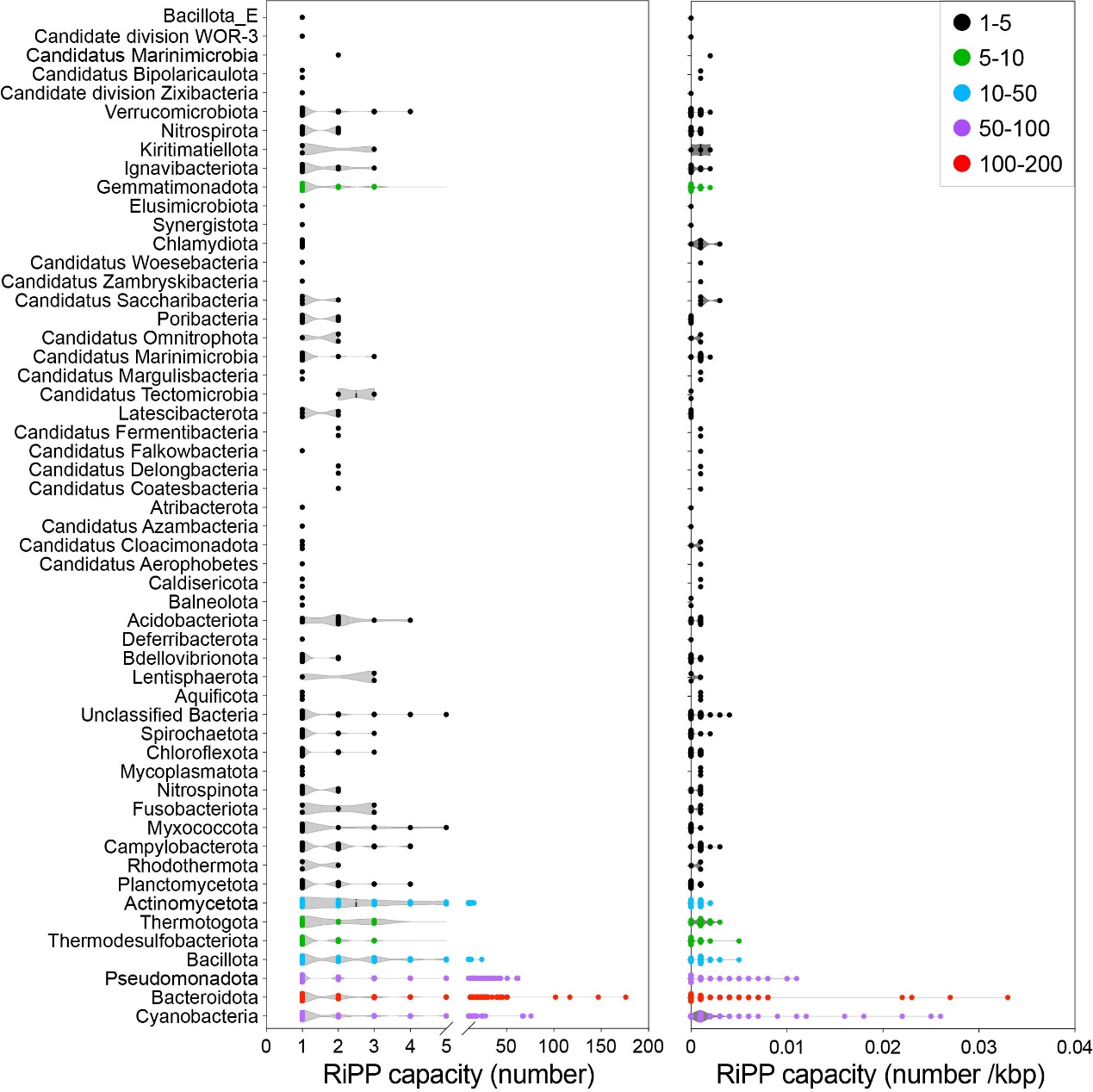


**Figure S17. The RiPP capacity of prokaryotic phylum from the MAR REF.** The figure shows the RiPP capacity defined as the number of RiPPs encoded in the genomes/scaffolds (left) and the number of RiPPs encoded in the genomes/scaffolds per kilobase pair (right). The y-axis represents the prokaryotic phylum and the color denotes the number of RiPPs. Source data can be found in **Supplementary Data 21, 22**.


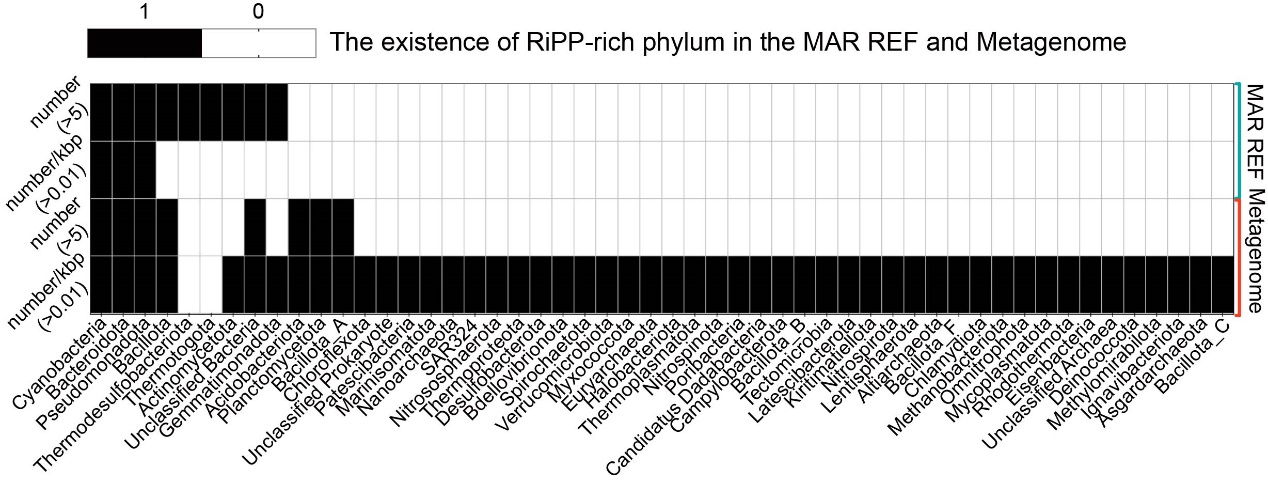


**Figure S18.** The existence of RiPP-rich phylum in the MAR REF and metagenomes. The “RiPP-rich” is defined as (1) the number of RiPP precursor sequences within genomes/contigs is larger than 5; (2) the number of RiPP precursor sequences within genomes/contigs per kilobase pair is larger than 0.01. The black and blank cells of the heatmap plot represent the existence of the RiPP-rich phylum in different sources. The phyla and the sources (MAR REF and Metagenome) were shown at the bottom and the right side, respectively.


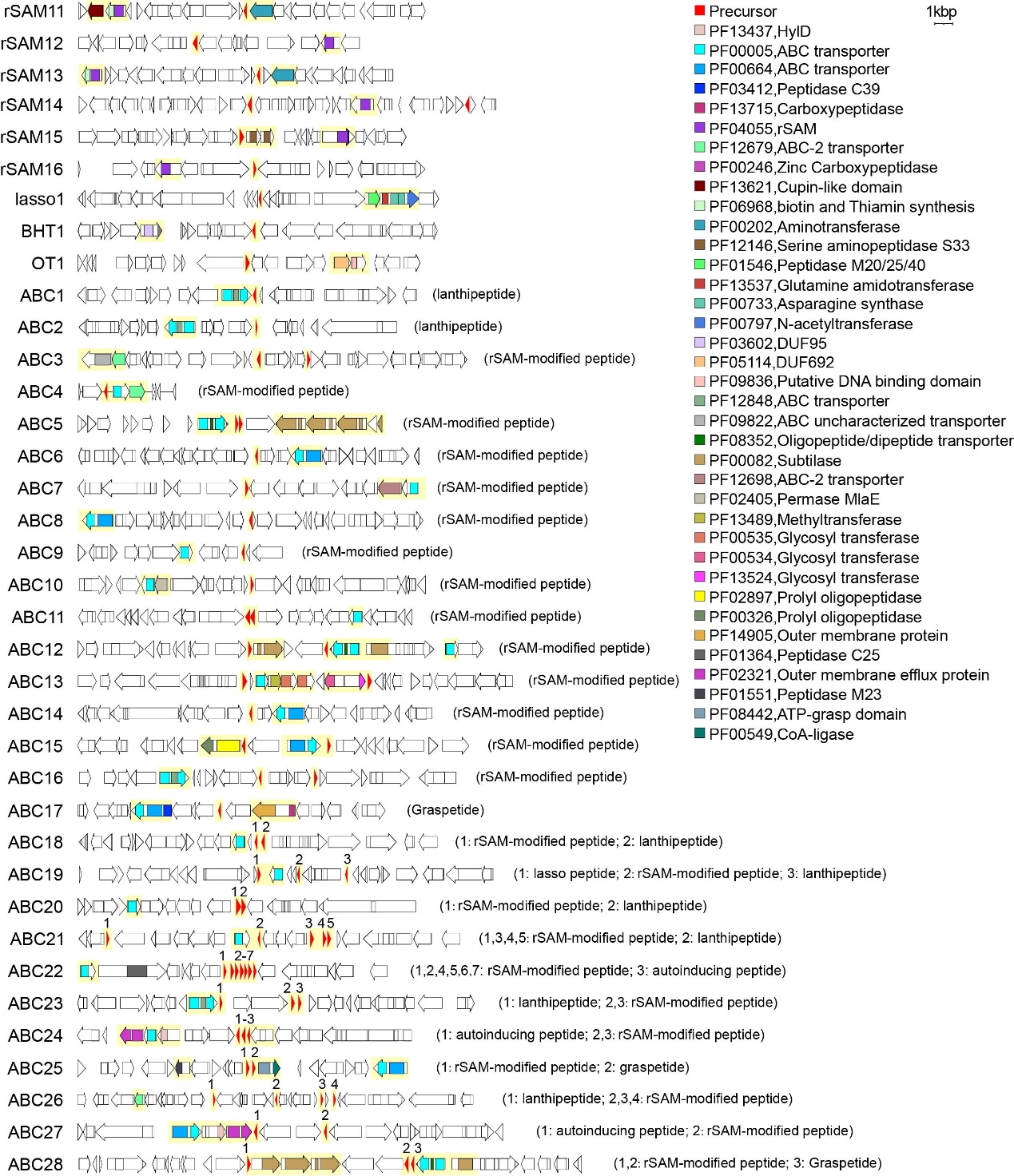


**Figure S19. The RiPP-encoding Biosynthetic Gene Clusters (BGCs) from Kordia (continued from Figure 3).** The core regions of BGCs were rendered with yellow backgrounds.For the BGCs not containing class-defining PTMs (ABC1-ABC28), the RiPP subclasses in the bracket represent the TrRiPP-predicted ones. For BGCs containing multiple precursors, the precursors were labeled separately using different numbers.


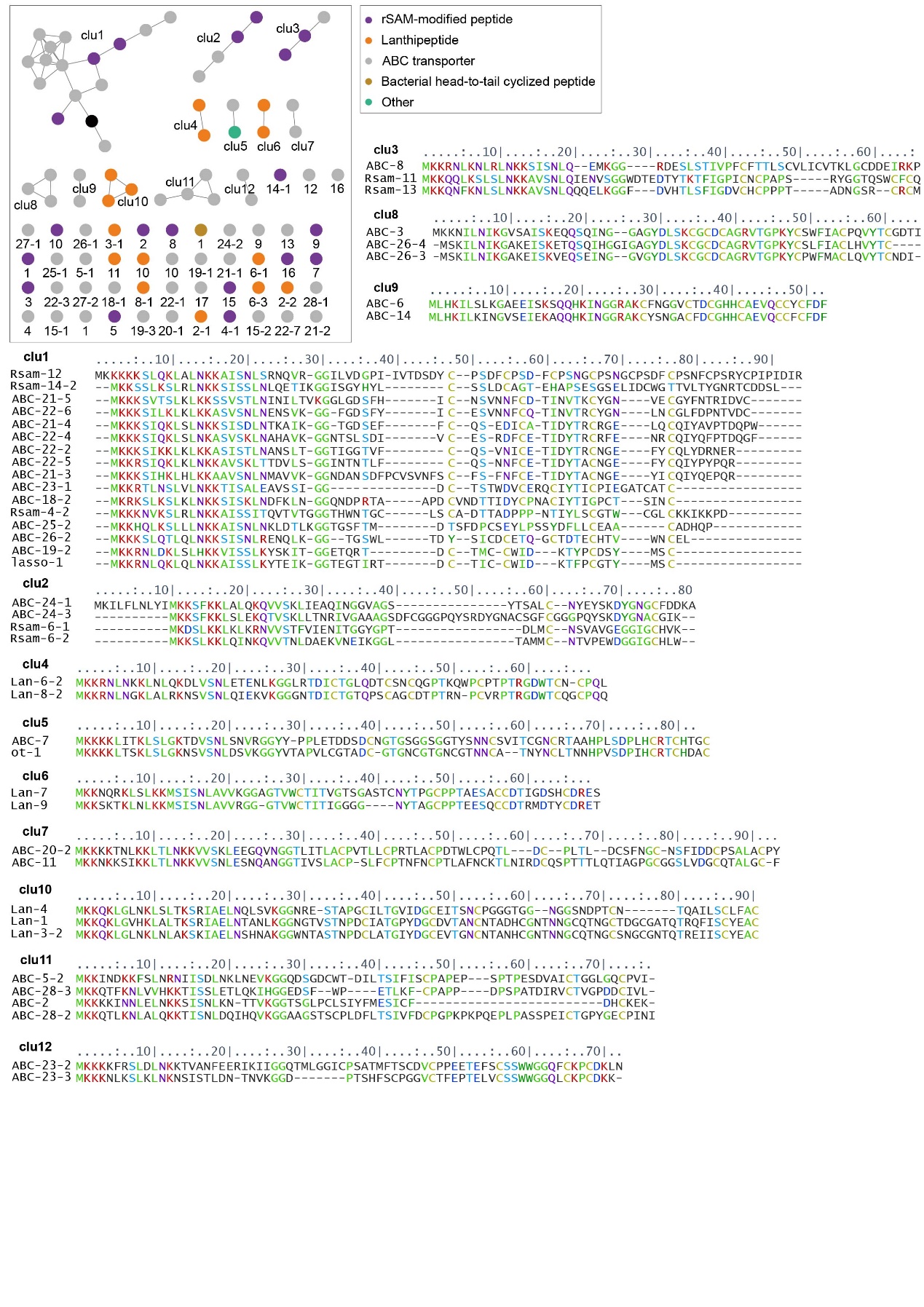


**Figure S20. The Sequence Similarity Network (SSN) and sequence logo of RiPP precursors from Kordia.** The SSN was constructed using EFI-EST (E-value=5, alignment score=8) and visualized by Cytoscape [13] and colored by RiPP subclasses (except ABC transporter). The sequence alignment of each cluster in SSN was generated using alignmentviewer (https://alignmentviewer.org/).


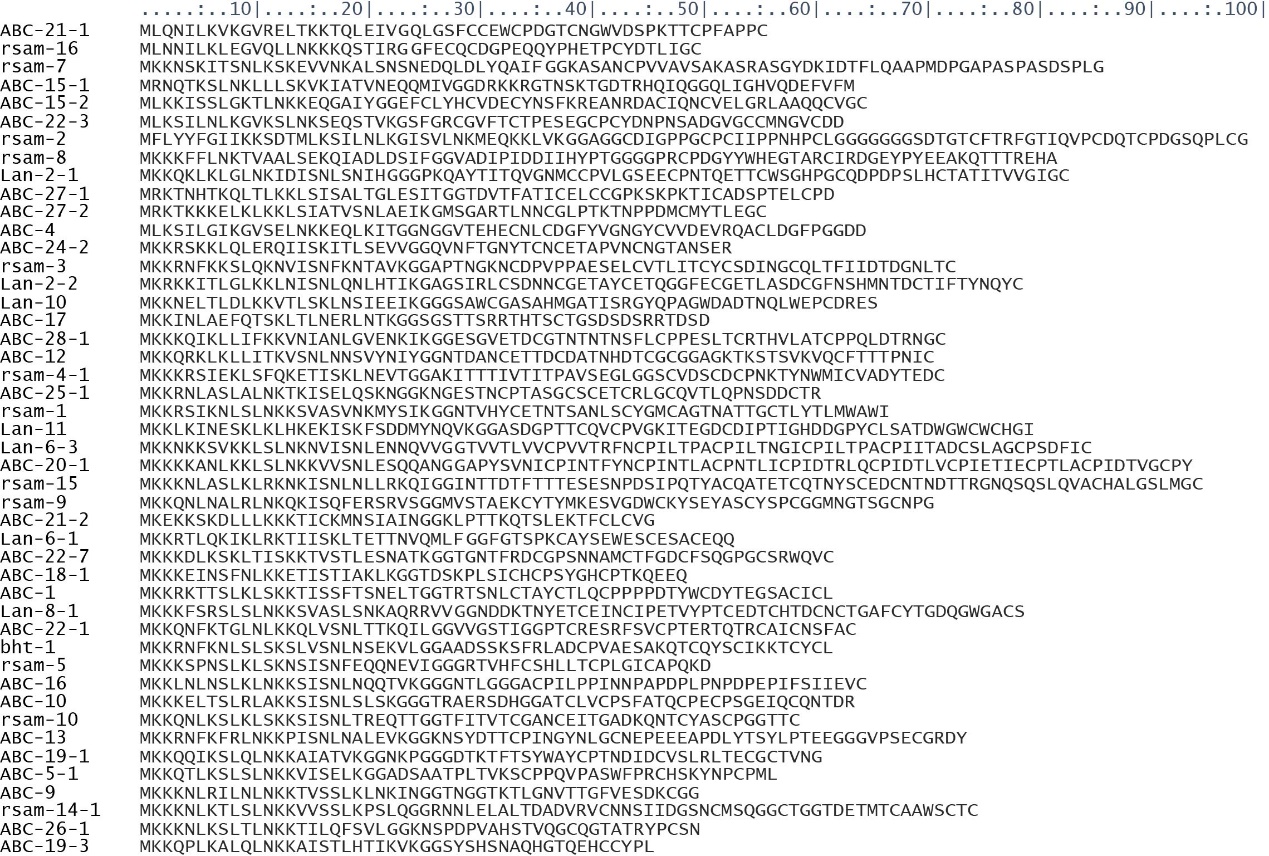


**Figure S20 (continued). The sequence of selected RiPP precursors from Kordia.** The RiPP precursor sequences that couldn’t be clustered with others were shown here.


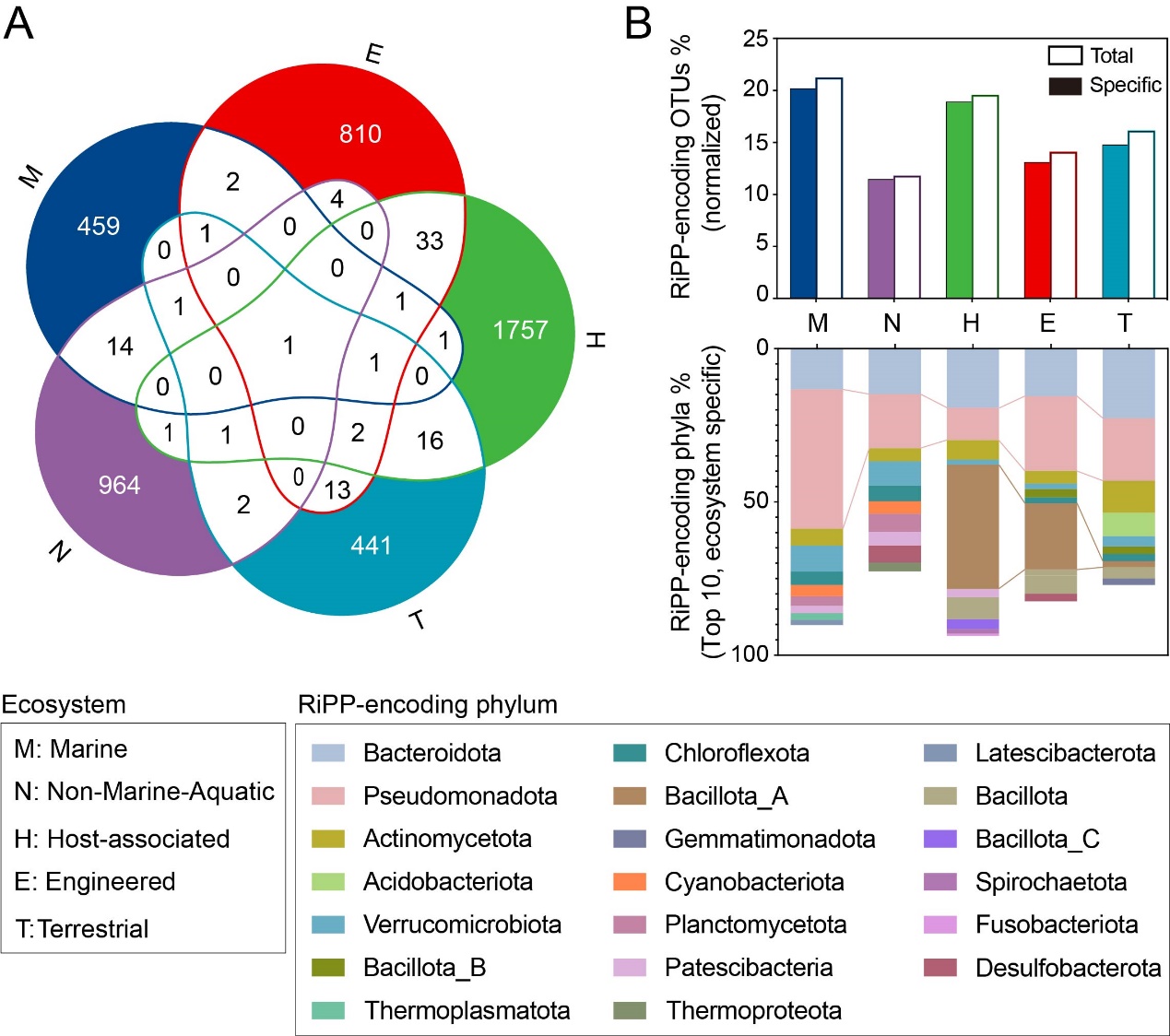


**Figure S21. Compare RiPP-encoding OTUs among five different ecosystems. A.** A five-set Venn figure visualizing the intersections of RiPP-encoding OTUs from five different ecosystems. The values in the figure represent the number of sharing or unique RiPP-encoding OTUs among ecosystems. The niche-specific parts were highlighted. **B.** The percentages of RiPP-encoding OTUs (top) and phyla (bottom). The normalized percentages (divide the average length of scaffolds from different ecosystems; see **Supplementary Information**) of RiPP-encoding OTUs among the whole detected OTUs were shown in solid bars and niche-specific ones were in hollow bars in the top figure. The percentage of niche-specific RiPP-encoding phyla among the detected OTUs is shown in the bottom figure. Only the top 10 phyla in five ecosystems were shown here.


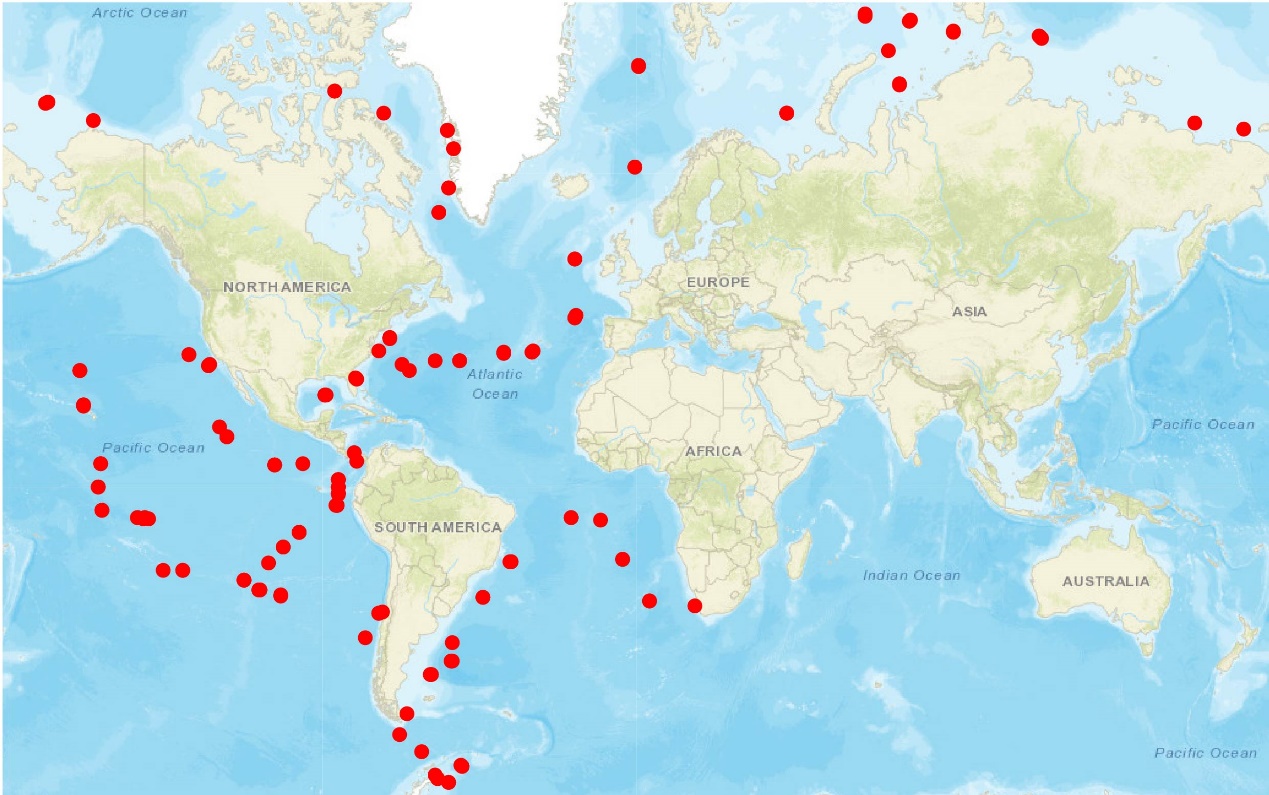


**Figure S22. Metatranscriptomic data collected from global ocean.** A total of 154 metatranscriptomic sequencing data were collected from 154 globally distributed sites (64S ~ 79N, 179W ~ 180E). Each red dot represents one sample. Map tiles © Esri.


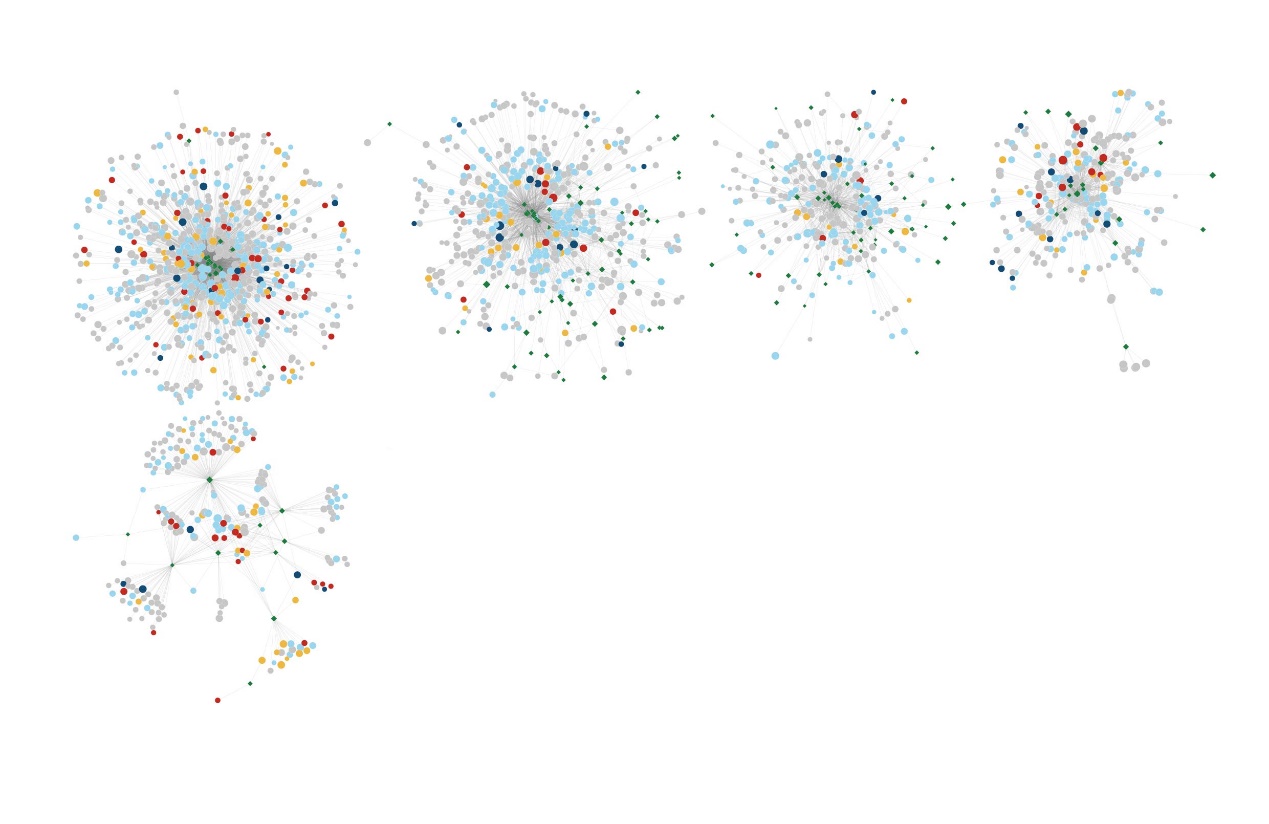


**Figure S23. The co-expression network of RiPP families and protein families (≤50 kbp away from RiPPs).** DefenseFinder and MobileOG-db were further applied to expand the MGEs, especially the “Defense-related” genes. Based on the expanded MGEs, the co-expression network of protein families (≤50 kbp away from RiPPs) and RiPP families were constructed and visualized using Cytoscape. For “Biosynthesis-related”, we focused on the region of 50kbp rather than the 10kbp in the vicinity of RiPPs proposed by antiSMASH. Because we considered the distantly and transcriptionally related biosynthetic proteins [14]. “Defense-related”, “Phage-related,” and “Other MGE-related” represent antiphage defense genes, integrated phage genes, and the Mobile genetic elements except for antiphage defense genes and integrated phage genes, respectively.


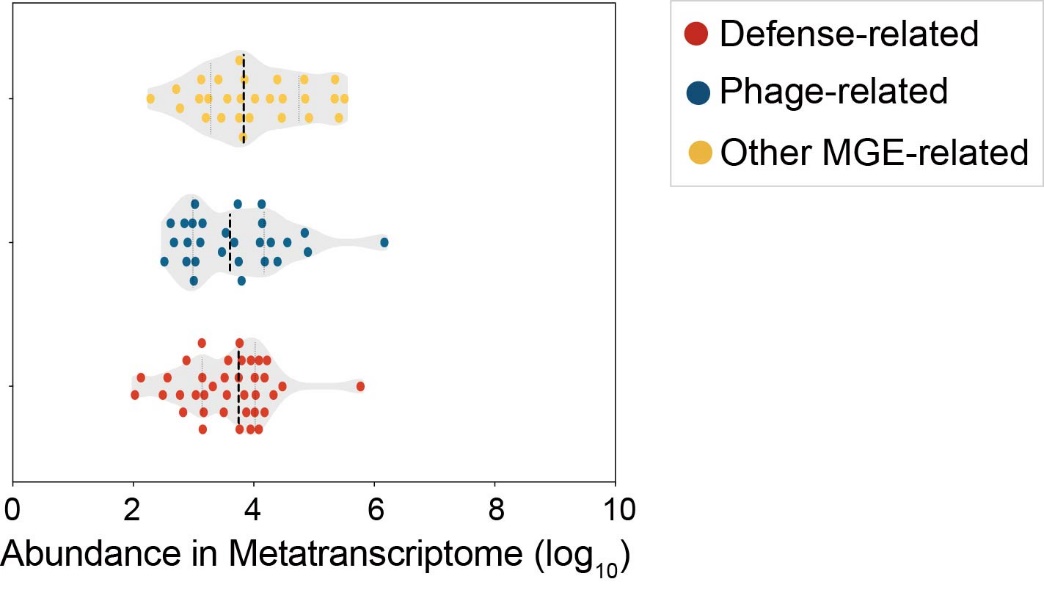


**Figure S24. The abundance of “Defense-related”, “Phage-related” and “Other MGE-related” genes correlated in modules with RiPP families in metatranscriptomes.** The truncated violin plot was used and the median and quartile values were shown as the dashed line in dark and grey respectively.


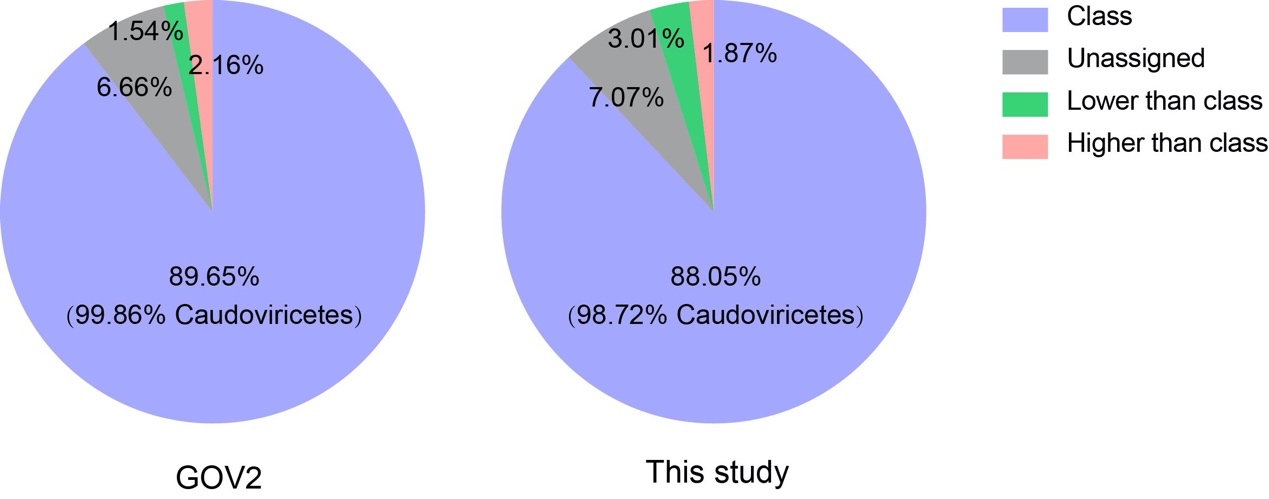


**Figure S25. The taxonomic distribution of GOV2 (left) and newly identified phage in this study (right) were shown separately.** To simplify the figure, only the class level (“Class”), the level lower than class (“Lower than class”), the level higher than class (“Higher than class”) and the unassigned phages (‘Unassigned’) were shown.


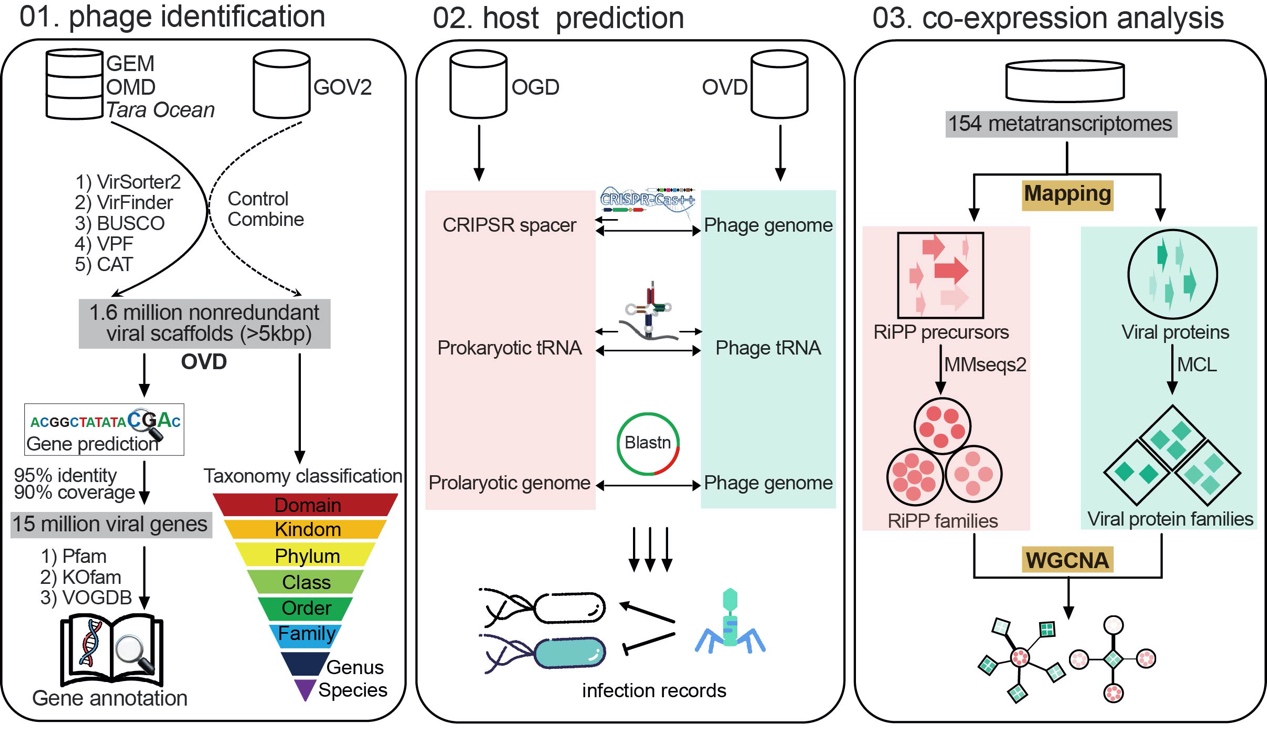


**Figure S26. The workflow of constructing the host-phage interaction network.** Step01. The identification of phage sequences starts from collecting scaffolds from public ocean genomic databases. The scaffolds are then discriminated by integrating results from VirSorter2 and VirFinder and detecting whether the sequence contains BUSCO-related host single-copy genes and viral family proteins (VPF; **Methods**). The Global Oceans Viromes (GOV2) is used as a control for the validation of this method. We combine the identified phage sequences in this study and GOV2 as Ocean Virus Database (OVD) with 1.6 million viral scaffolds (99% identity). The details of gene annotation and taxonomy classification can be found in Methods. Step02. We leverage the constructed databases OGD and OVD, containing 21171 prokaryotic genomes/scaffolds and 1,647,134 viral scaffolds, respectively, as the host-phage pairwise searching pool. We build the host-phage connectivities based on the host-encoded CRISPR spacers, shared tRNA and host-integrated prophage (**Methods**). Step03. The RiPP precursors and viral proteins are clustered into families. Then, transcript profiling of RiPP families and viral protein families is based on the aggregation of reads mapping to the corresponding genes. Then, WGCNA is used for the co-expression analysis of RiPP families and viral protein families (**Methods**).


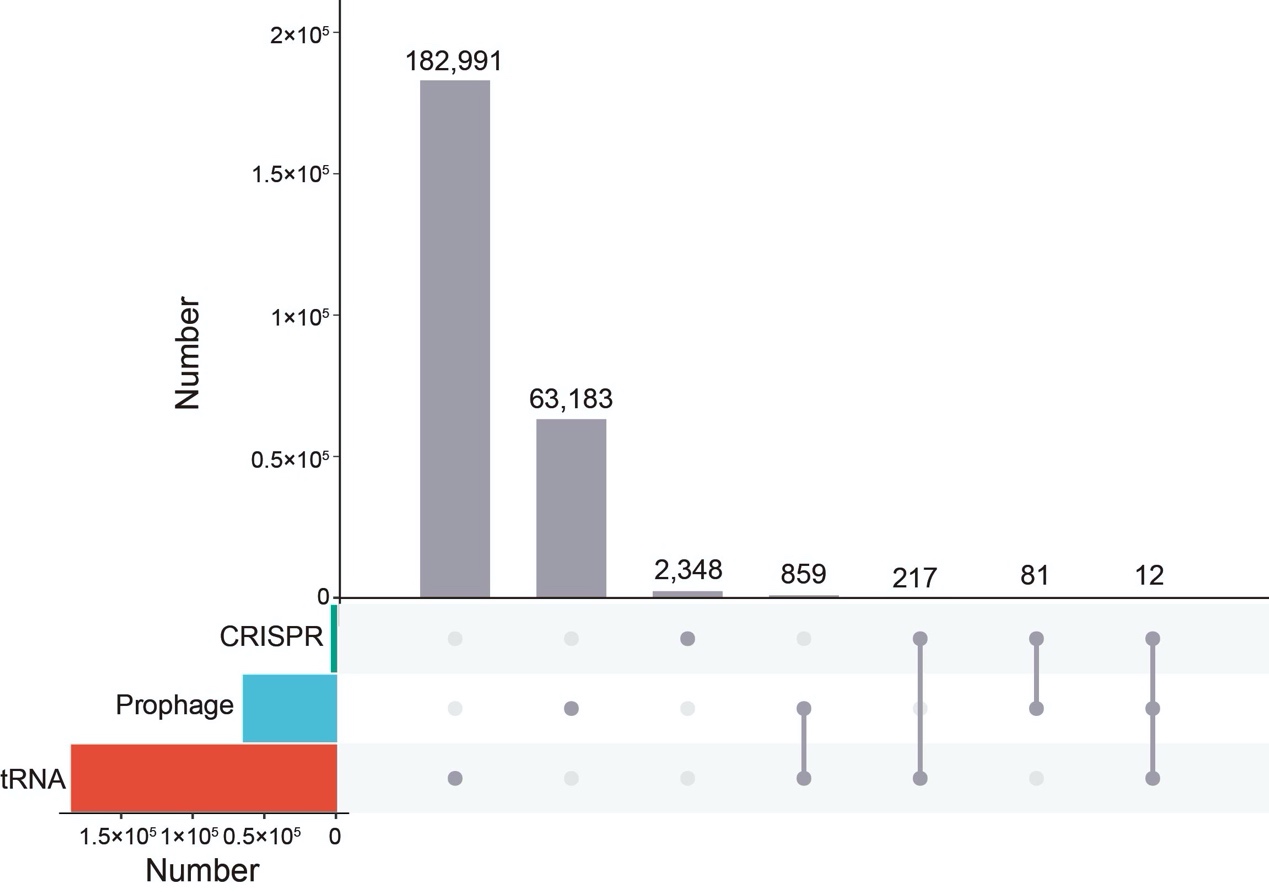


**Figure S27. The phage-host relationship linked by different methods.** The workflow of identifying phage-host relationships can be found in **Figure S26** and **Methods**. This figure shows the number of phage-host relationships detected based on different methods and the overlaps among them.


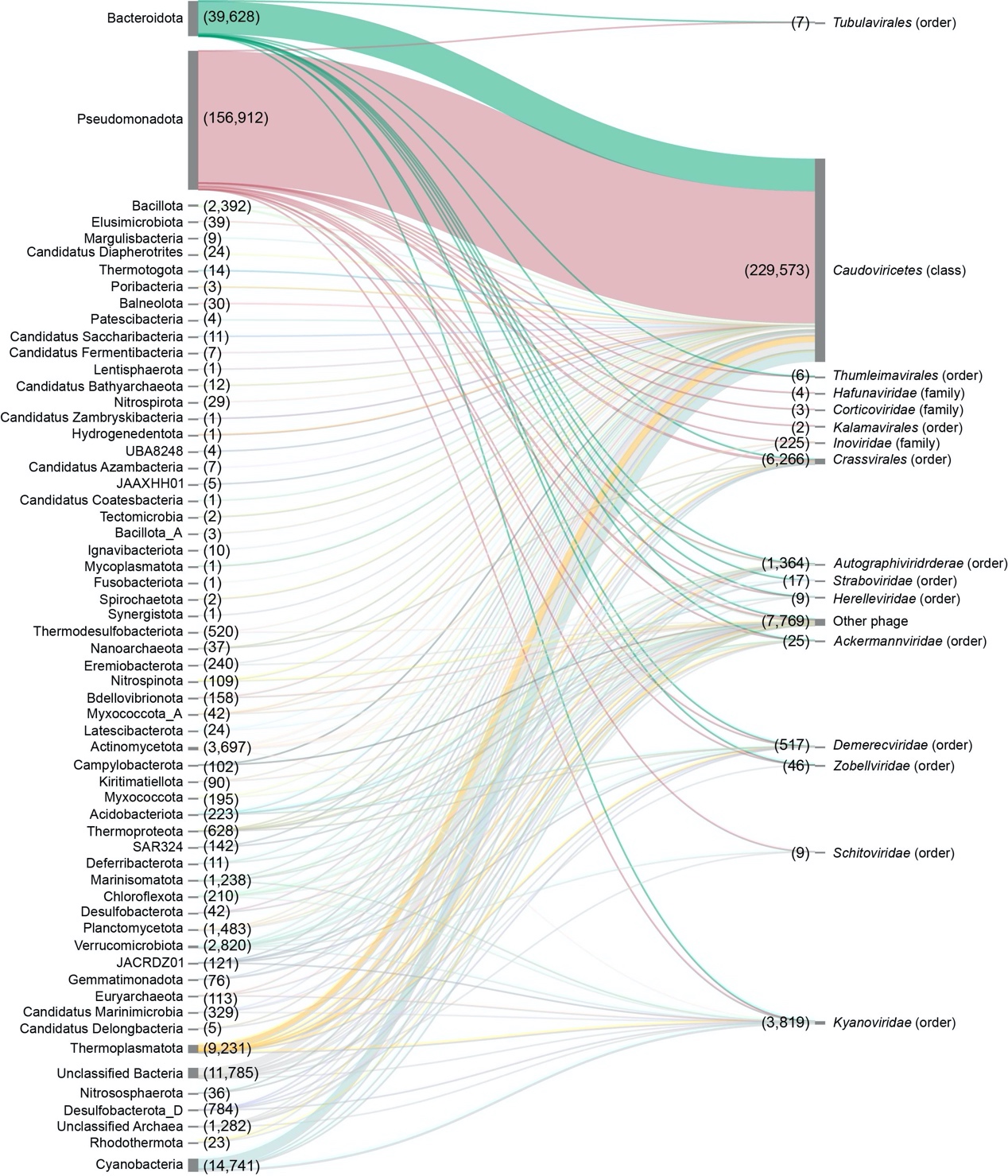


**Figure S28. The network of host-phage relationships containing 249,692 nonredundant host-phage links.** The node column denoted the prokaryote phyla (left) and phage taxon (right). The numbers in the brackets represented the flows between nodes. For example, Pseudomonadota was the source of 156,912 flows and Caudoviricetes was the target of 229,573 flows. The string ‘class’, ‘order’ and ‘family’ in the bracket represented the phage class, order, and family.


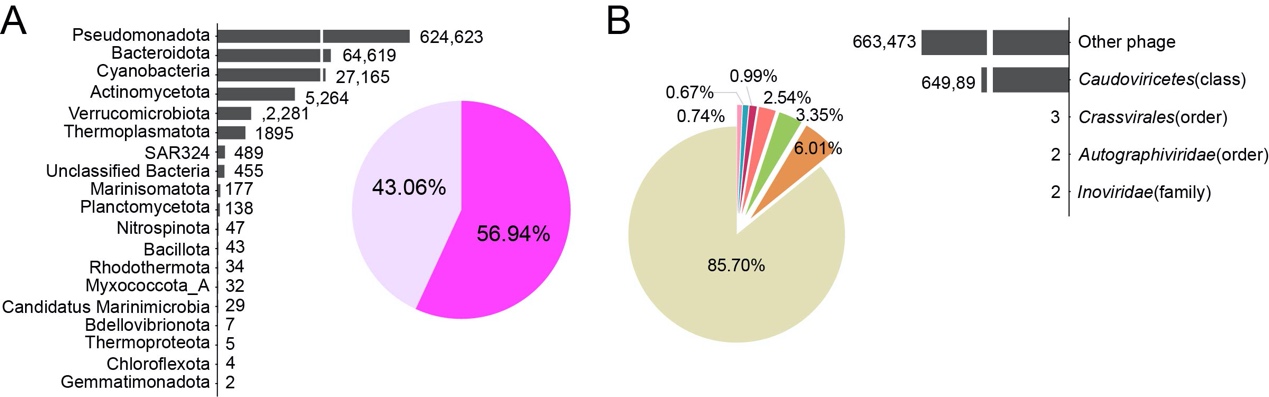


**Figure S29.** **The summary of the whole RiPP-involving prokaryote-phage interaction network.** **A**. The bar plot showed the distribution of flows from prokaryotic phyla. The y-axis showed the prokaryotic phyla and the values of flows were on the straight right side of the bars. The pie chart showed the distribution of flows from RiPP families, which were separated based on confidence (at least one member of the RiPP family having flanking PTMs within 10 genes in the vicinity is defined as high confidence, and vice versa). **B**. The bar plot showed the distribution of flows to phage taxon. The y-axis showed the phage taxon and the values of flows were on the straight left side of the bars. The pie chart shows the distribution of flows to the functional categories of viral protein families. The phages involved in the network include 2,572 Caudoviricetes, 775 unassigned phages, 22 Heunggongvirae and 2 Inoviridae.


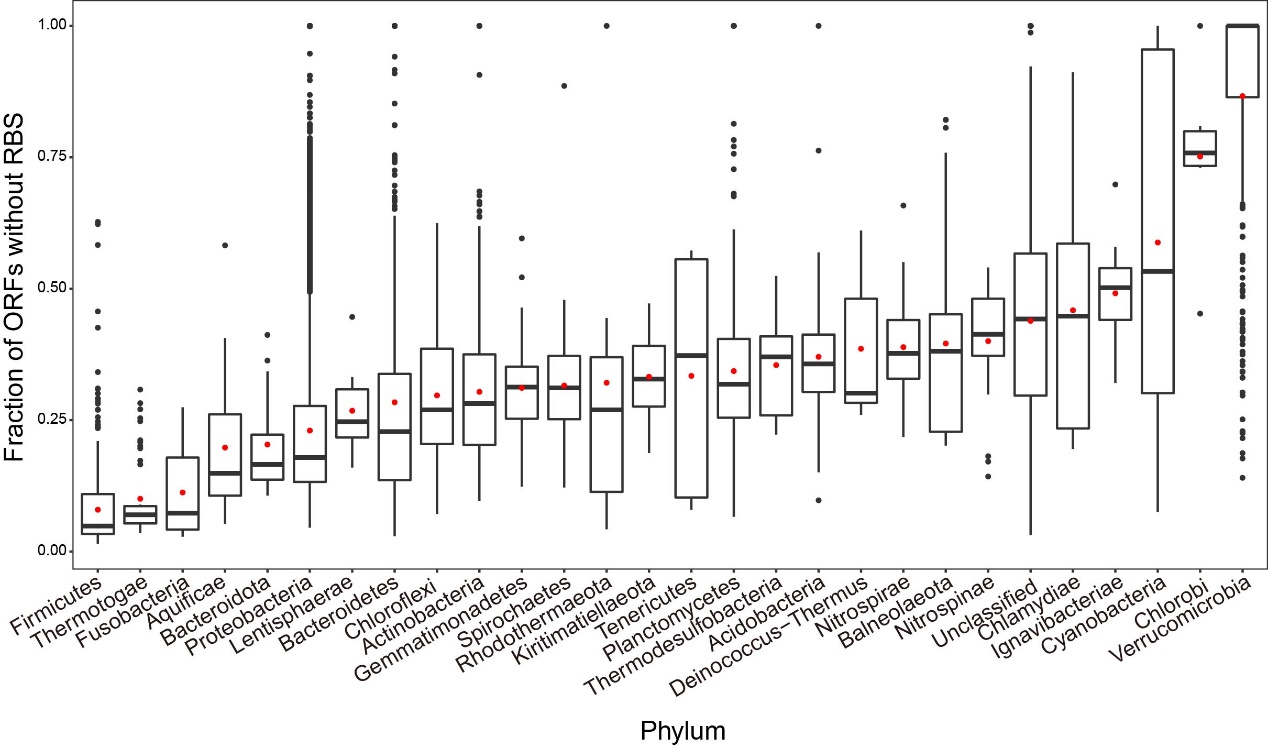


**Figure S30. The proportion of Prodigal-short predicted ORFs without RBS in isolated marine bacterial genomes.** Phyla with less than 5 genomes and Candidatus phyla were removed. In each box, the center bar represents the median, the red dot represents the mean, the box represents the upper and lower quartiles, and the whiskers represent 1.5 times the interquartile range (IQR); black dots are outliers outside the 1.5 x IQR range. For the top 5 phyla: Cyanobacteria, Chlorobi, Chlamydiae, Ignavibacteriae, and Verrucomicrobia, their ORFs were not filtered by RBS.


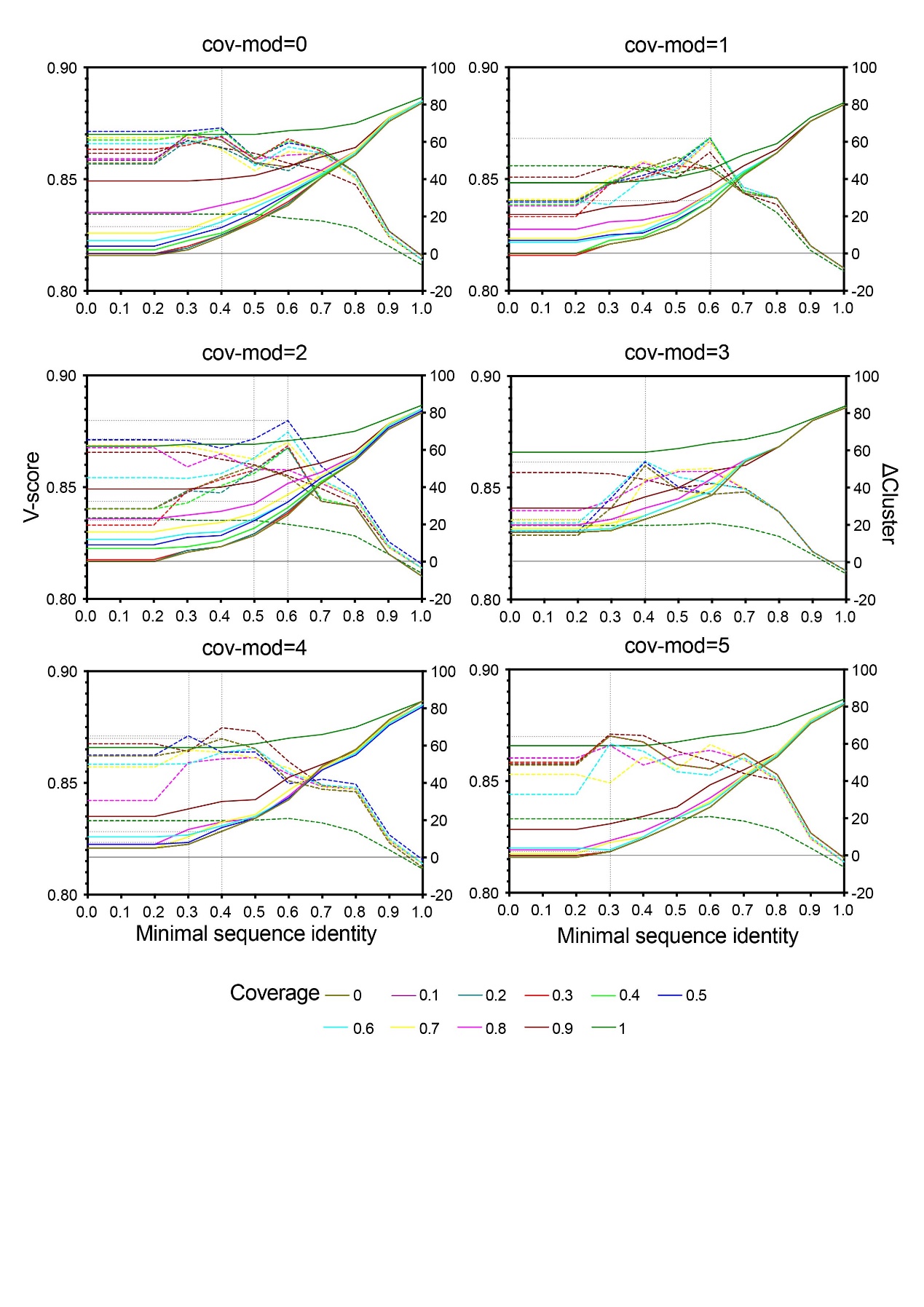


**Figure S31. The optimization of MMSeqs2 clustering parameters by mapping the grouping of 193 known RiPP precursors with links to NPAtlas compounds.** The optimized parameters were coverage (-c), coverage mode (-cov-mod), and minimal sequence identity (--min-seq-id). Clustering agreements between NPAtlas families of the compounds and MMSeqs-derived RiPP families are compared across different thresholds, as measured by the v-score (dashed line) and differences in the total number of RiPP families (solid line). In the end, the minimal sequence identity of 0.5, 0.6, 0.7 and coverage of 0.4, 0.5, and 0.6 showed better agreement with NPAtlas family agreement (high v-score and low difference in the total number of RiPP families), were retained for the second-round optimization.


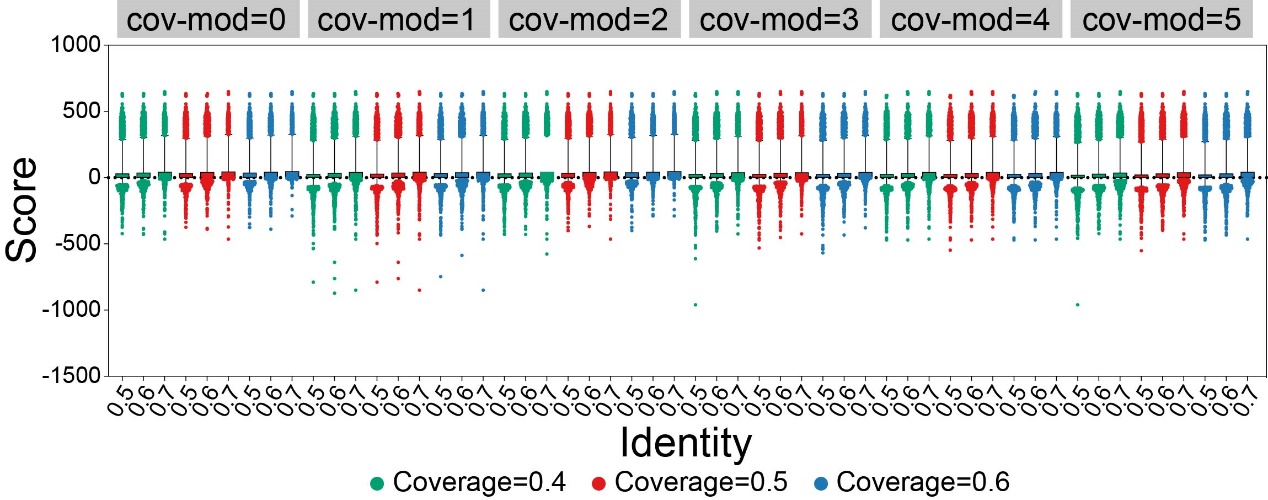


**Figure S32. The optimization of MMSeqs2 clustering parameters by minimizing the number of RiPP families with low sequence alignment scores.** The optimized parameters were coverage (-c), coverage mode (-cov-mod) and minimal sequence identity (--min-seq-id). GLAM2 was used to discover sequence motifs and calculate the sequence alignment scores of RiPP families. The alignment scores of RiPP families derived by different MMSeqs clustering parameters were shown in the box plot (5-95 percentile). In the end, “-c 0.6 --min-seq-id 0.6 -cov-mod 2” were picked as the optimized parameters to use for our analysis, which showed the balanced distribution of the alignment scores of RiPP families.


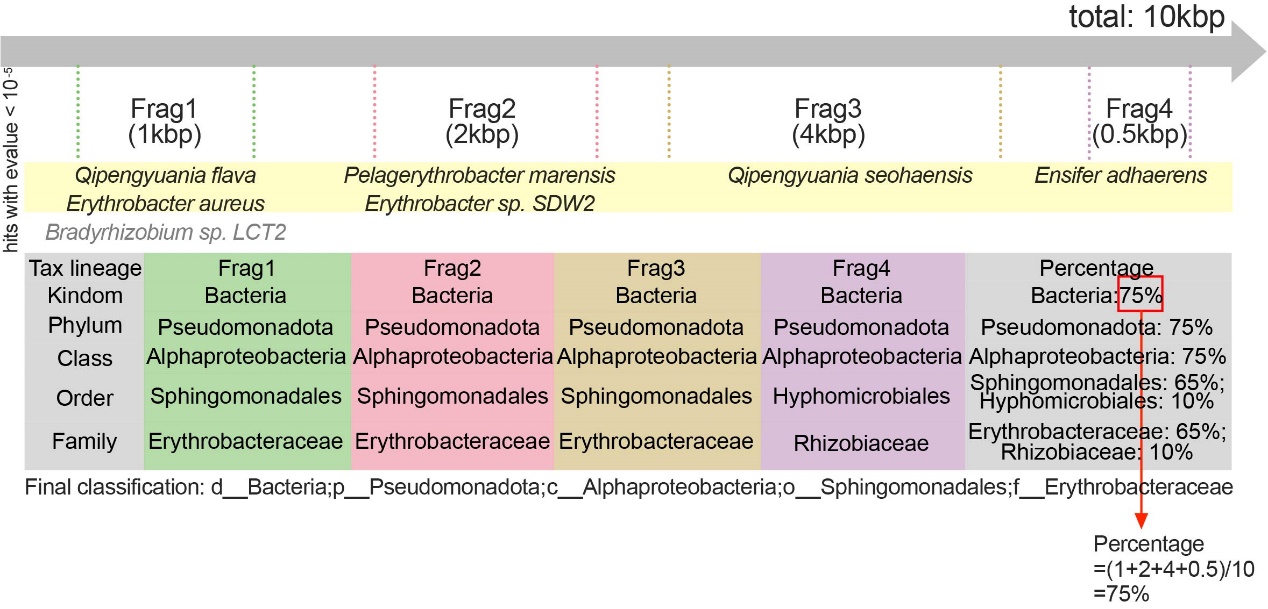


**Figure S33. Voting-based taxonomy classification.** The scaffolds and genomes are first queried against the NCBI non-redundant nucleotide database (NT) using MEGABLAST (v2.12.0+). The fragments are individually classified based on the LCA of all hits with evalue<0.00001. Hits in gray are not included in the final annotation of the fragment. The classification of scaffolds or genomes is based on a voting approach of all classified fragments, by summing all lengths of fragments supporting a certain classification. The scaffold or genome is classified as the classification with the highest percentage and at least 50% of the whole length of the scaffold or genome. The final classification is shown at the bottom.


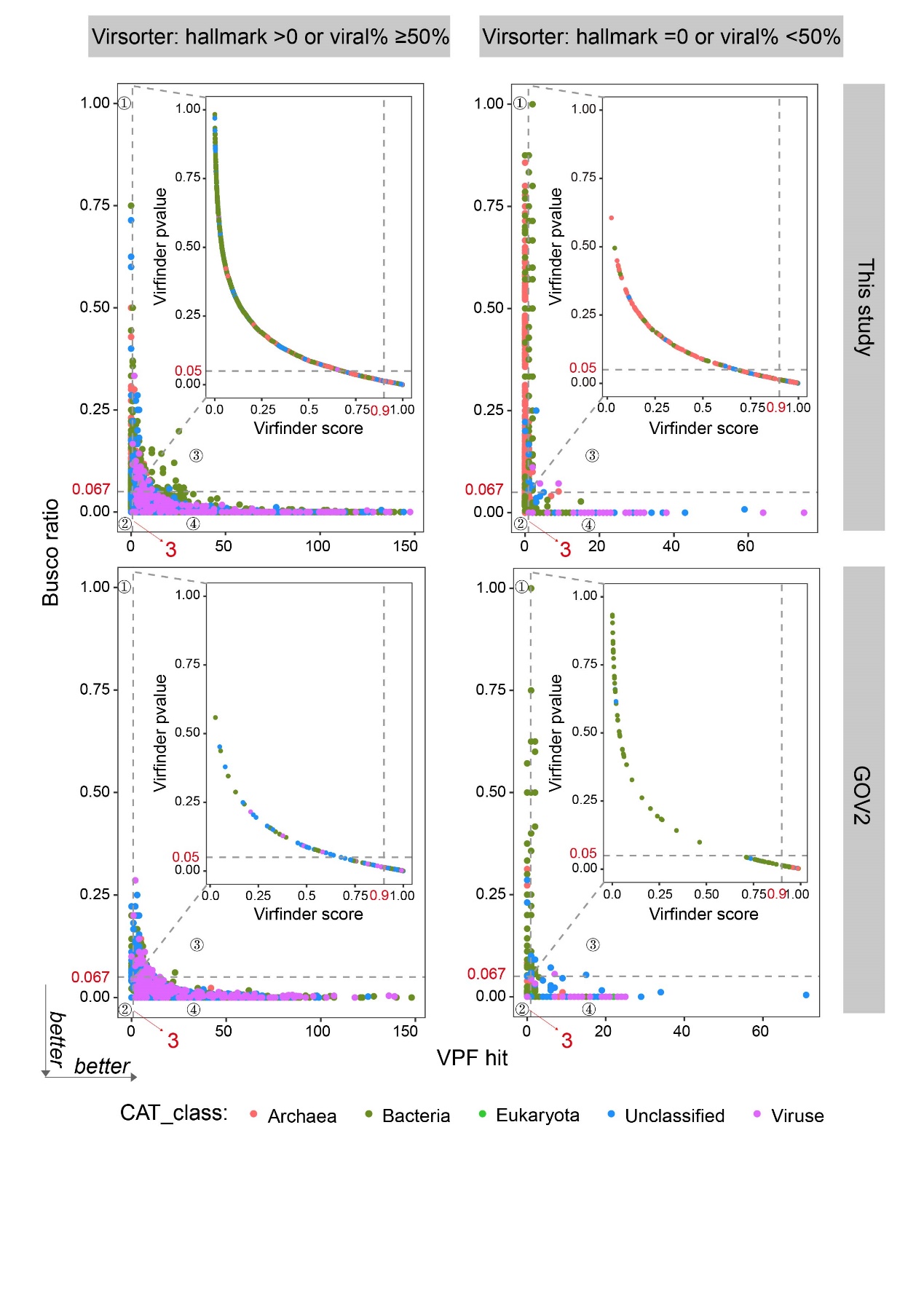


**Figure S34. The identified viral sequences from GOV2 and the Metagenomes in this study using defined principles.** The workflow of viral sequence identification can be found in **Figure S26** and **Methods.** We separate the VirSorter results into two parts: (1) hallmark >0 or viral% > 50%, representing high confidence in VirSorter2; (2) hallmark = 0 or viral% < 50%, representing low confidence in VirSorter2. The points from different phyla are rendered with different colors. The purple points (“Viruse”) tend to locate around the right bottom region, revealing the performance of our method. The x-axis represents the number of viral proteins hit to VPF. The y-axis represents the percentage of viral proteins hit to BUSCO-related host single-copy genes.


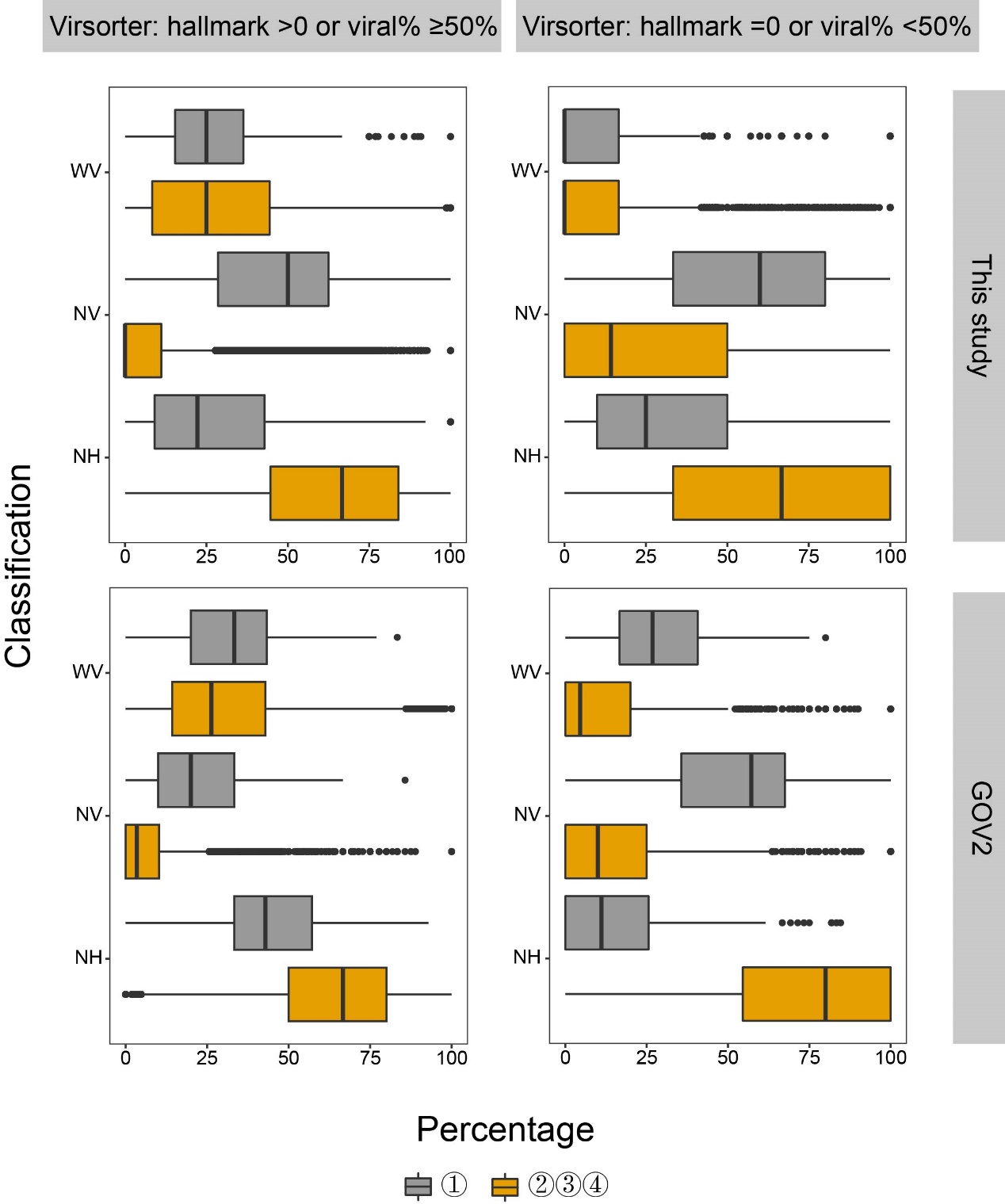


**Figure S35. Compare annotations of viral genes from GOV2 and Metagenomes in this study**. The Pfam-A, VOGDB and KOfam databases are used for the viral gene annotations. The classification, WV: significant hits against VOGDB; NV: no significant hits against VOGDB; NH: no significant hits against three databases. We separate the VirSorter results into two parts: (1) hallmark >0 or viral% > 50%, representing high confidence in virSorter2; (2) hallmark = 0 or viral% < 50%, representing low confidence in VirSorter2. The legend represents the four regions in **Figure. S34**. Region 2,3 and 4 (yellow boxes) meet the thresholds of BUSCO and VPF (see Method Details), representing the high confidence of the candidate viral sequence. In these regions (yellow boxes), more sequences contain uncharacterized proteins (could not find any homologies in the three databases), and fewer sequences contain proteins that could not find in VOGDB.


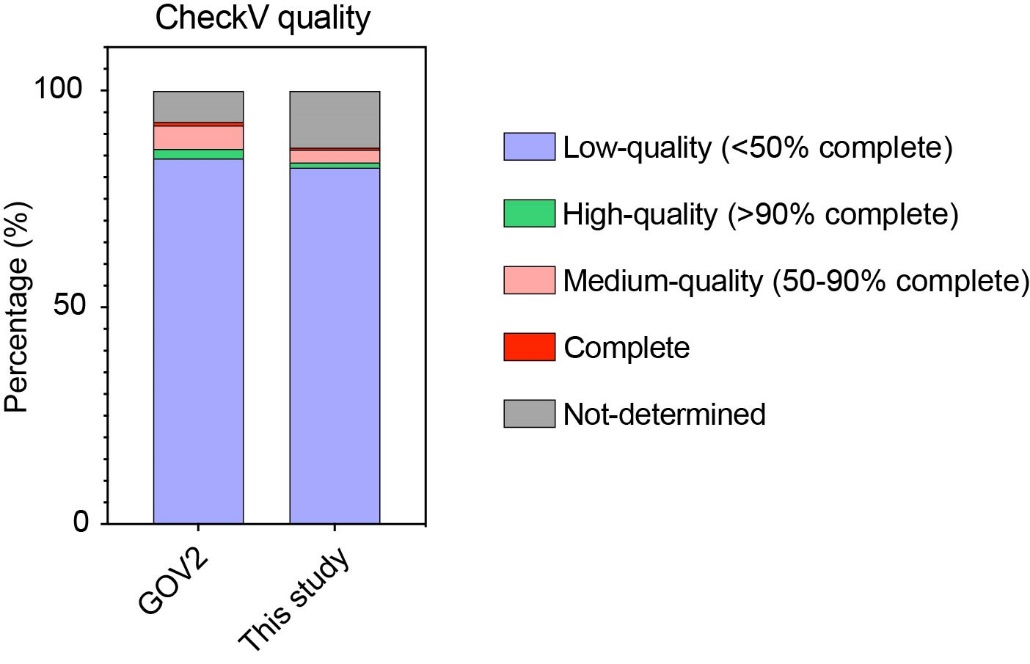


**Figure S36. The quality of GOV2 and newly identified viral sequences in this study evaluated by CheckV.**


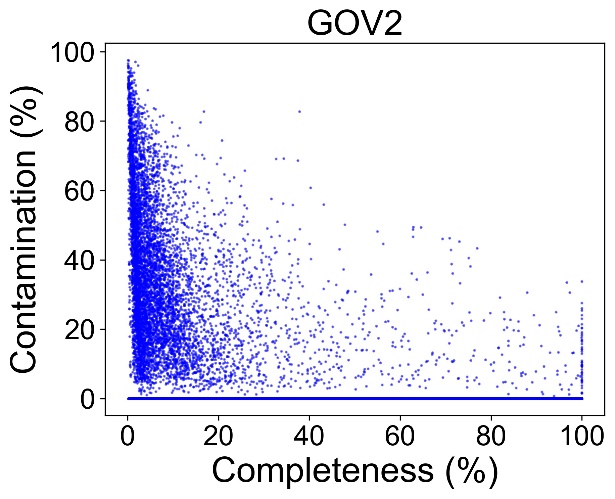

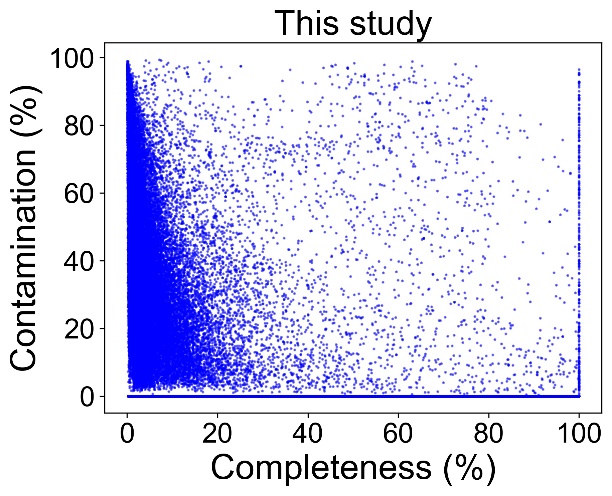


**Figure S37. The completeness and contamination of GOV2 and newly identified viral sequences in this study evaluated by CheckV.**


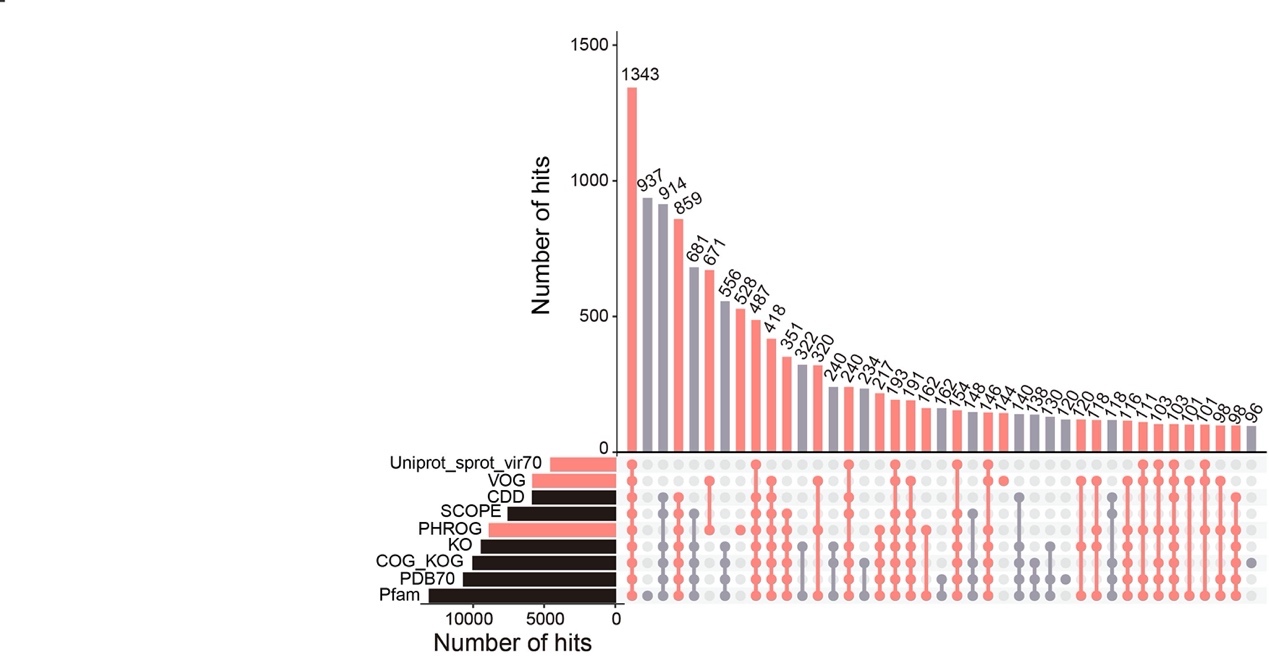


**Figure S38. Viral gene families annotated against different protein databases.** The details of annotations can be found in Methods. This figure shows the number of viral protein families annotated against different databases and the overlaps among them. The viral protein databases and the related intersections are highlighted in red.


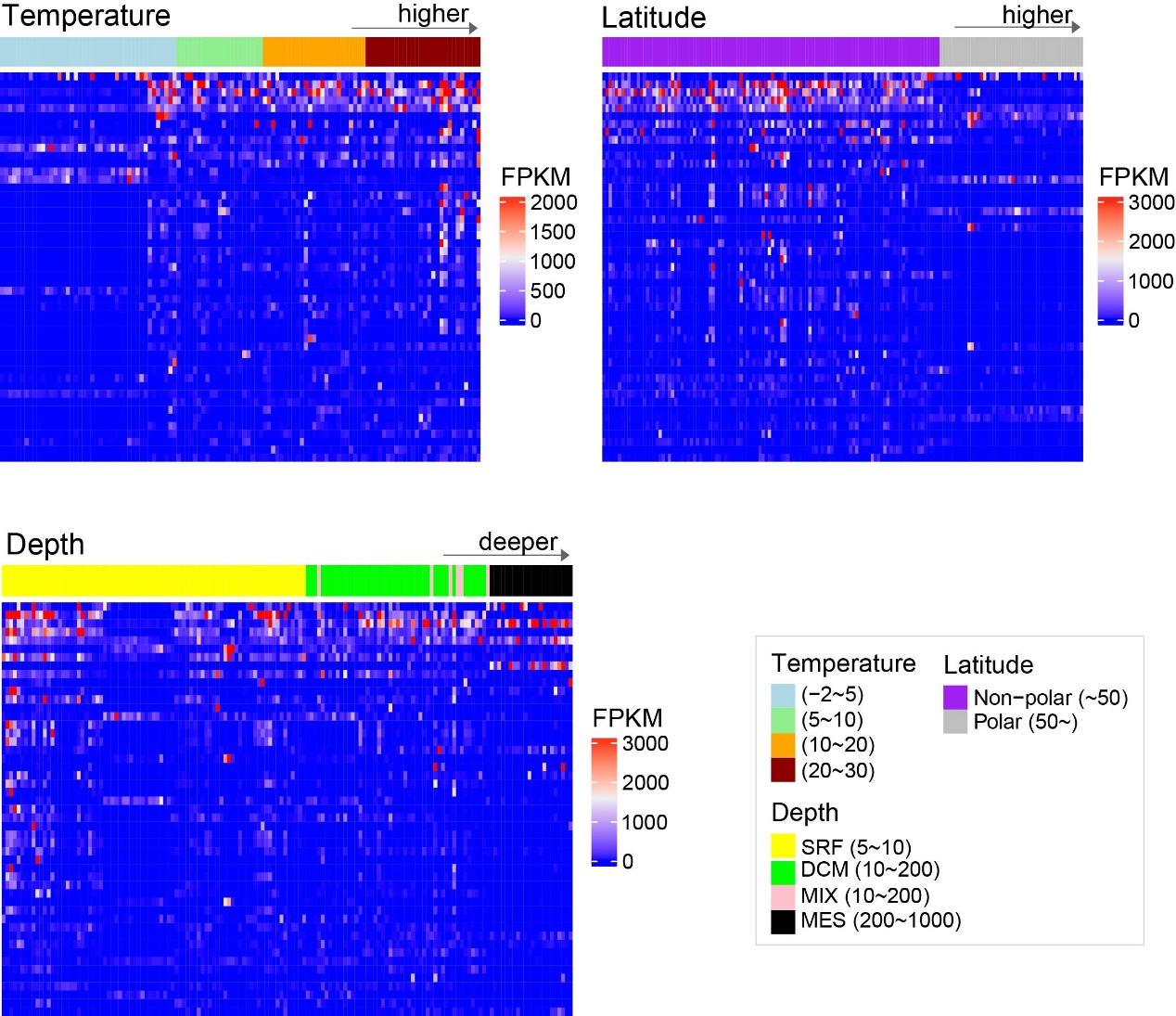


**Figure S39. The influence of different environmental factors on the transcriptions of RiPPs.** The explored environmental factors are temperature, depth and latitude. The figures show only the top 50 RiPP families with the highest abundance of transcripts for visualization. The higher temperature and lower latitude show higher transcription levels of RiPP families.


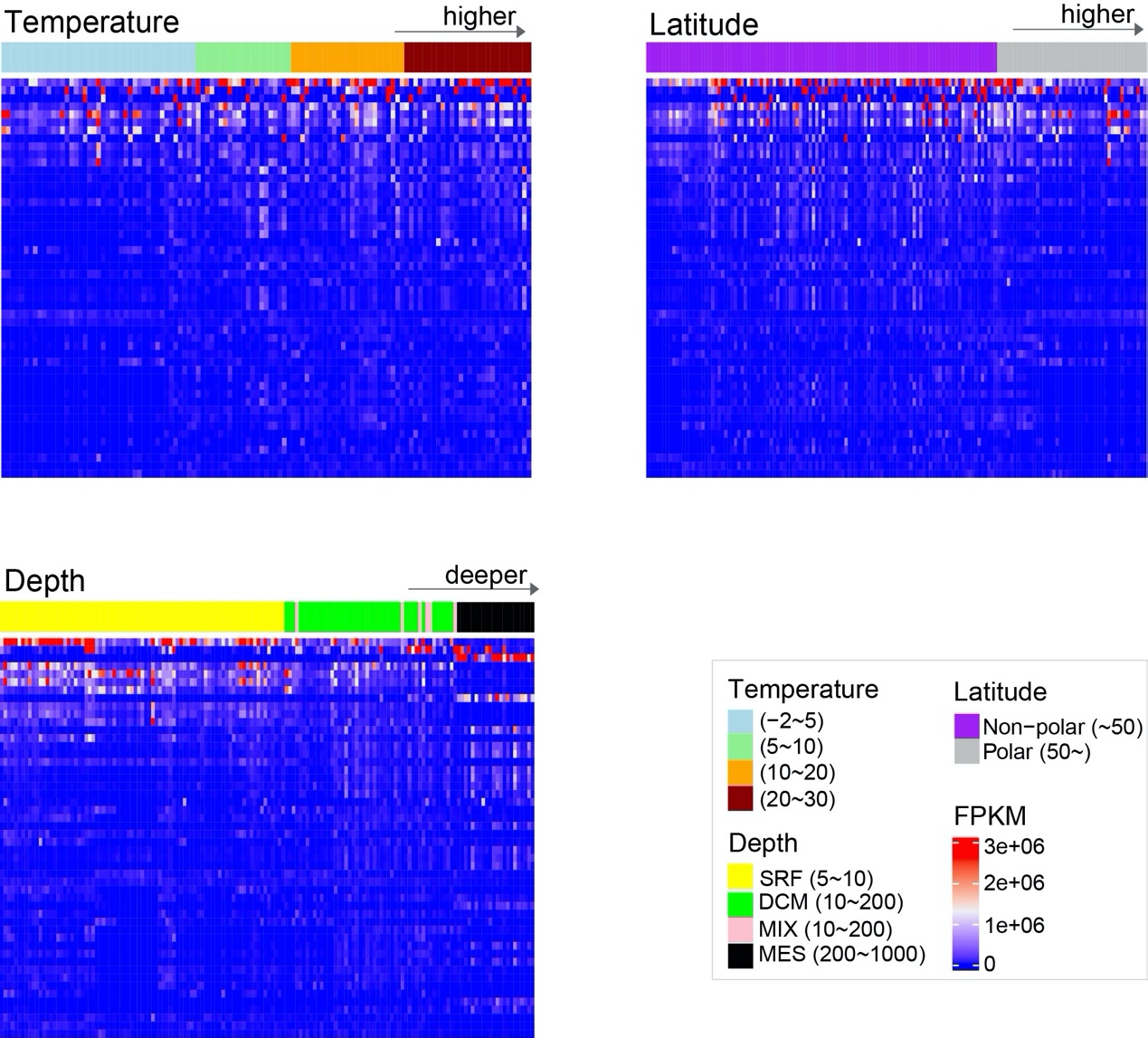


**Figure S40. The influence of different environmental factors on the transcriptions of viral proteins.** The explored environmental factors are temperature, depth and latitude. The figures show only the top 50 viral protein families with the highest abundance of transcripts for visualization. The higher temperature and lower latitude show higher transcription levels of RiPP families. Some layer-specific activate viral protein families also exist.


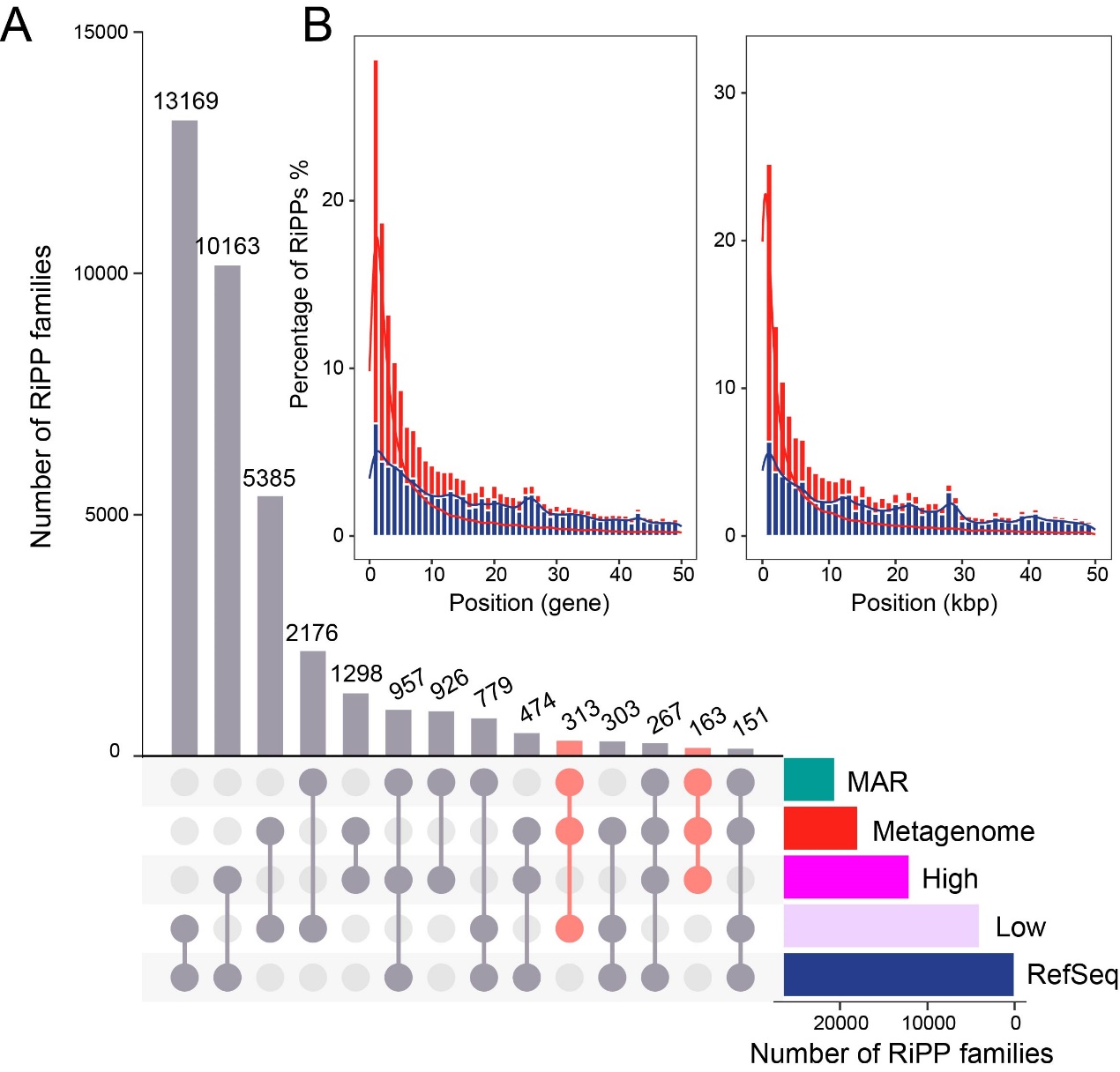


**Figure S41. A. The distribution of RiPP families with high or low confidence from MAR, Metagenome and RefSeq.** The intersections between MAR and Metagenome were highlighted in red. **B. The length distribution of RiPPs-encoding genes flanking regions.** The number of genes (left) and kilobase pairs (right) of flanking regions including both upstream and downstream in metagenome and RefSeq were shown in two figures.

**Supplementary Table**

**Supplementary Table 1. Model performance comparison of DeepRiPP, NeuRiPP and TrRiPP on binary prediction task (RiPP vs. non-RiPP).** Values represent the average of the results of a 10-fold cross validation. The highest values in each column are highlighted in bold. For NeuRiPP, all 5 models were evaluated**.**

| **Model** | **Accuracy** | **Precision** | **Recall** | **F1** | **MCC** | **AUROC** |
| --- | --- | --- | --- | --- | --- | --- |
| NeuRiPP-cnn-parallel-lstm | 0.990 | 0.973 | 0.912 | 0.942 | 0.937 | 0.989 |
| NeuRiPP-cnn-parallel | 0.987 | 0.963 | 0.889 | 0.925 | 0.919 | 0.982 |
| NeuRiPP-cnn-linear | 0.987 | 0.948 | 0.898 | 0.922 | 0.916 | 0.983 |
| NeuRiPP-cnn-linear-lstm | 0.990 | 0.960 | 0.921 | 0.940 | 0.935 | 0.988 |
| NeuRiPP-lstm | 0.988 | 0.932 | 0.939 | 0.935 | 0.929 | 0.991 |
| DeepRiPP | 0.970 | **0.987** | 0.660 | 0.790 | 0.793 | 0.947 |
| **TrRiPP** | **0.994** | 0.981 | 0.953 | **0.967** | **0.964** | **0.995** |

**Supplementary Table 2. Model performance comparison of DeepRiPP and TrRiPP on multiclass prediction task (RiPP classification).** Values represent the average of the results of a 10-fold cross validation. The highest value in each column are highlighted in bold.

| **Model** | **Accuracy** | **Weighted F1** | **Cohen’s kappa** |
| --- | --- | --- | --- |
| DeepRiPP | 0.969 | 0.960 | 0.776 |
| **TrRiPP** | **0.994** | **0.993** | **0.960** |

**Supplementary Table 3. Comparison of RiPPs predicted by antiSMASH and TrRiPP.** RiPP precursors annotated by antiSMASH were grouped to the classes used in TrRiPP. N/A: no precursor was identified by antiSAMSH for this RiPP class.

| **Class** | **AntiSMASH annotated class** | **AntiSMASH annotated precursor** | **TrRiPP predicted precursor** | **AntiSMASH annotated precursor (deduplicated)** | **TrRiPP predicted precursor (deduplicated)** |
| --- | --- | --- | --- | --- | --- |
| Autoinducing peptide | cyclic-lactone-autoinducer | N/A | 4833 | N/A | 1328 |
| Bacterial head-to-tail cyclized peptide | sactipeptide | N/A | 1096 | N/A | 273 |
| Cyanobactin | cyanobactin | N/A | 559 | N/A | 164 |
| Lanthipeptide | lanthipeptide | 4956 | 7866 | 2371 | 3374 |
| Lasso peptide | lassopeptide | 1069 | 5248 | 560 | 2076 |
| rSAM modified peptide | RaS-RiPP, ranthipeptide, sactipeptide | 228 | 7216 | 12 | 2817 |
| Thiopeptide | thiopeptide | 117 | 763 | 70 | 320 |
| Graspetide | microviridin | N/A | 489 | N/A | 323 |
| Other | linaridin, thioamitides, proteusin, bottromycin, epipeptide, glycocin, guanidinotides, LAP, lipolanthine, spliceotide | N/A | 5404 | N/A | 1746 |

**Supplementary Table 4. Amino acid and PTM enzyme requirements of RiPPs.** Neighboring ORFs are defined as ORFs within 10kb genetic context of the precursor gene.

| **Class** | **Precursor AA requirement** | **PTM enzyme requirement** |
| --- | --- | --- |
| Autoinducing peptide | Cys or Ser | Neighboring ORFs have PF04647 |
| Bacterial head-to-tail cyclized peptide | - | Neighboring ORFs have PF01944 |
| Cyanobactin | - | Neighboring ORFs have PF00082 or PF18047 or PF18065 |
| Lanthipeptide | Cys, and Ser or Thr | Neighboring ORFs have PF05147 |
| Lasso peptide | Asp or Glu | Neighboring ORFs have PF00733 |
| rSAM modified peptide | Cys or Trp or Tyr or His | Neighboring ORFs have PF04055 |
| Thiopeptide | Cys, and Ser or Thr | Neighboring ORFs have PF02624 |
| Graspetide | Asp or Glu,  and Ser or Thr or Lys | Neighboring ORFs have ‘ATP-grasp’^#^ |

# Searched by InterProScan instead of hmmsearch.

**Supplementary Table 5**. **The enrichment analysis (hypergeometric distribution) of the MGE-related genes in the co-expression modules among the entire set of MGE-related genes in metatranscriptomes.** P-values were corrected for multiple testing using the method by Benjamini & Hochberg [15] with an excepted false discovery rate (FDR) of FDR = 0.05. The absent values of module 4, 6 and 8 represent that no MGE-related genes were detected in these modules.

| **Module** | **Hypergeometric distribution (*P*)** | **FDR** |
| --- | --- | --- |
| 1 | 0.4986707 | 0.5485370 |
| 2 | 1.523631e-08 | 4.189985e-08 |
| 3 | 3.074958e-06 | 6.764908e-06 |
| 4 | / | / |
| 5 | 0.7540217 | 0.7540217 |
| 6 | / | / |
| 7 | 0.02530543 | 0.03479497 |
| 8 | / | / |

**Supplementary Table 6**. **The enrichment analysis (hypergeometric distribution) of the biosynthesis-related genes in the co-expression modules among the entire set of biosynthesis-related genes in metatranscriptomes.** P-values were corrected for multiple testing using the method by Benjamini & Hochberg [15] with an excepted false discovery rate (FDR) of FDR = 0.05. The absent values of module 6 and 8 represent that no biosynthesis-related genes were detected in these modules.

| **Module** | **Hypergeometric distribution (*P*)** | **FDR** |
| --- | --- | --- |
| 1 | 1.690323e-11 | 9.296777e-11 |
| 2 | 5.31312e-12 | 5.844432e-11 |
| 3 | 0.003070269 | 0.004824708e |
| 4 | 0.2642225 | 0.3229386 |
| 5 | 1.751864e-09 | 6.423501e-09 |
| 6 | / | / |
| 7 | 1.13372e-4 | 2.078488e-4 |
| 8 | / | / |

**Supplementary Table 7. The principles of host predictions using different tools.**

| **Tools** | **Principles** |
| --- | --- |
| CRISPRCasFinder | (1) blastn-short (-evalue 0.00001 -word_size 7);  (2) $\leq$2 mismatch/gap; |
| tRNA-scan SE | (1) blastn (-evalue 0.00001);  (2) filter promiscuous tRNA;  (3) $\leq$2 mismatch/gap; |
| MEGABLAST | (1) identity $\geq$ 70%;  (2) match $\geq$ 2500bp;  (3) match $\geq$ 30% viral coverage;  (4) $\geq$ 30% of the host contig to be outside the prophage region alignment; |

**References**

1. Blin K, Shaw S, Kloosterman AM, Charlop-Powers Z, van Wezel GP, Medema MH*, et al.* antiSMASH 6.0: improving cluster detection and comparison capabilities. *Nucleic Acids Res.* 2021;**49:** W29-W35.

2. O'Leary NA, Wright MW, Brister JR, Ciufo S, McVeigh DHR, Rajput B*, et al.* Reference sequence (RefSeq) database at NCBI: current status, taxonomic expansion, and functional annotation. *Nucleic Acids Res.* 2016;**44:** D733-D745.

3. Paoli L, Ruscheweyh HJ, Forneris CC, Hubrich F, Kautsar S, Bhushan A*, et al.* Biosynthetic potential of the global ocean microbiome. *Nature.* 2022;**607:** 111-118.

4. Kautsar SA, Blin K, Shaw S, Weber T, Medema MH. BiG-FAM: the biosynthetic gene cluster families database. *Nucleic Acids Res.* 2021;**49:** D490-D497.

5. Terlouw BR, Blin K, Navarro-Munoz JC, Avalon NE, Chevrette MG, Egbert S*, et al.* MIBiG 3.0: a community-driven effort to annotate experimentally validated biosynthetic gene clusters. *Nucleic Acids Res.* 2023;**51:** D603-D610.

6. Navarro-Munoz JC, Selem-Mojica N, Mullowney MW, Kautsar SA, Tryon JH, Parkinson EI*, et al.* A computational framework to explore large-scale biosynthetic diversity. *Nat Chem Biol.* 2020;**16:** 60-68.

7. Montalban-Lopez M, Scott TA, Ramesh S, Rahman IR, van Heel AJ, Viel JH*, et al.* New developments in RiPP discovery, enzymology and engineering. *Nat Prod Rep.* 2021;**38:** 130-239.

8. Zyubko T, Serebryakova M, Andreeva J, Metelev M, Lippens G, Dubiley S*, et al.* Efficient in vivo synthesis of lasso peptide pseudomycoidin proceeds in the absence of both the leader and the leader peptidase. *Chem Sci.* 2019;**10:** 9699-9707.

9. Duan YW, Niu WJ, Pang LL, Bian XY, Zhang YM, Zhong GN. Unusual Post-Translational Modifications in the Biosynthesis of Lasso Peptides. *Int J Mol Sci.* 2022;**23:** 7231.

10. Clark KA, Bushin LB, Seyedsayamdost MR. RaS-RiPPs in Streptococci and the Human Microbiome. *ACS Bio Med Chem Au.* 2022;**2:** 328-339.

11. Schmidt EW, Nelson JT, Rasko DA, Sudek S, Eisen JA, Haygood MG*, et al.* Patellamide A and C biosynthesis by a microcin-like pathway in Prochloron didemni, the cyanobacterial symbiont of Lissoclinum patella. *Proc Natl Acad Sci U S A.* 2005;**102:** 7315-7320.

12. Agarwal V, Pierce E, McIntosh J, Schmidt EW, Nair SK. Structures of Cyanobactin Maturation Enzymes Define a Family of Transamidating Proteases. *Chem Biol.* 2012;**19:** 1411-1422.

13. Shannon P, Markiel A, Ozier O, Baliga NS, Wang JT, Ramage D*, et al.* Cytoscape: A software environment for integrated models of biomolecular interaction networks. *Genome Res.* 2003;**13:** 2498-2504.

14. Crits-Christoph A, Diamond S, Butterfield CN, Thomas BC, Banfield JF. Novel soil bacteria possess diverse genes for secondary metabolite biosynthesis. *Nature.* 2018;**558:** 440-444.

15. Benjamini Y, Hochberg Y. Controlling the False Discovery Rate - a Practical and Powerful Approach to Multiple Testing. *J R Stat Soc B.* 1995;**57:** 289-300.
